# *Yarrowia lipolytica *vesicle-mediated protein transport pathways

**DOI:** 10.1186/1471-2148-7-219

**Published:** 2007-11-12

**Authors:** Dominique Swennen, Jean-Marie Beckerich

**Affiliations:** 1 Laboratoire de Microbiologie et Génétique Moléculaire INRA-CNRS-AgroParisTech UMR 1238 CBAI BP01 F-78850 Thiverval Grignon, France

## Abstract

**Background:**

Protein secretion is a universal cellular process involving vesicles which bud and fuse between organelles to bring proteins to their final destination. Vesicle budding is mediated by protein coats; vesicle targeting and fusion depend on Rab GTPase, tethering factors and SNARE complexes. The Génolevures II sequencing project made available entire genome sequences of four hemiascomycetous yeasts, *Yarrowia lipolytica*, *Debaryomyces hansenii*, *Kluyveromyces lactis *and *Candida glabrata*. *Y. lipolytica *is a dimorphic yeast and has good capacities to secrete proteins. The translocation of nascent protein through the endoplasmic reticulum membrane was well studied in *Y. lipolytica *and is largely co-translational as in the mammalian protein secretion pathway.

**Results:**

We identified *S. cerevisiae *proteins involved in vesicular secretion and these protein sequences were used for the BLAST searches against Génolevures protein database (*Y. lipolytica*, *C. glabrata*, *K. lactis *and *D. hansenii*). These proteins are well conserved between these yeasts and *Saccharomyces cerevisiae*. We note several specificities of *Y. lipolytica *which may be related to its good protein secretion capacities and to its dimorphic aspect. An expansion of the *Y. lipolytica *Rab protein family was observed with autoBLAST and the Rab2- and Rab4-related members were identified with BLAST against NCBI protein database. An expansion of this family is also found in filamentous fungi and may reflect the greater complexity of the *Y. lipolytica *secretion pathway. The Rab4p-related protein may play a role in membrane recycling as *rab4 *deleted strain shows a modification of colony morphology, dimorphic transition and permeability. Similarly, we find three copies of the gene (*SSO*) encoding the plasma membrane SNARE protein. Quantification of the percentages of proteins with the greatest homology between *S. cerevisiae, Y. lipolytica *and animal homologues involved in vesicular transport shows that 40% of *Y. lipolytica *proteins are closer to animal ones, whereas they are only 13% in the case of *S. cerevisiae*.

**Conclusion:**

These results provide further support for the idea, previously noted about the endoplasmic reticulum translocation pathway, that *Y. lipolytica *is more representative of vesicular secretion of animals and other fungi than is *S. cerevisiae*.

## Background

*Yarrowia lipolytica *is a hemiascomycetous dimorphic yeast, generally regarded as safe (GRAS), which has been used for biotechnological applications. It is able to produce large amounts of several metabolites such as citric acid and to secrete a variety of extracellular proteins (alkaline or acid proteases, RNase, lipases *etc*.) [1]. Its good protein secretion capacities have allowed the engineering of powerful heterologous protein expression systems [reviewed in [2]]. *Y. lipolytica *is also a conveniently tractable model organism, of which the secretion pathway was studied for several years in our laboratory [3]. We focused on the early steps of protein translocation in the endoplasmic reticulum [4-8], on the quality control of protein folding [9] and on the glycosylation pathway [10]. Several genes involved in these steps were cloned and analysed.

The results of the Génolevures II sequencing project of four hemiascomycetous yeasts [11] allowed us to search for proteins involved in the secretion pathway of *Y. lipolytica *and we compared them to the proteins of the three other yeasts, *Candida glabrata*, *Kluyveromyces lactis *and *Debaryomyces hansenii*. *C. glabrata *has become the second most common cause of candidiasis after *Candida albicans*. *C. glabrata *is not dimorphic, in contrast to other *Candida *species, and is phylogenetically closer to *Saccharomyces cerevisiae *[12]. *K. lactis *is less closely related to *S. cerevisiae *and has the capacity to grow on lactose as a sole carbon source, it has been used for industrial applications [13,14]. *D. hansenii *is a cryotolerant marine yeast which grows at salinities up to 24%. *D. hansenii *is the most common yeast found in cheese and provides proteolytic and lipolytic activities during cheese ripening [15]. In this work, we first established the list of proteins, predicted from whole genome analysis, which are potentially involved in vesicular transport in *Y. lipolytica*. Candidates were identified through BLAST searches against *S. cerevisiae *protein sequences. We then search for homologues of these proteins in the predicted protein set encoded by the three other genomes. Among the differences observed, we noticed a number of plasma membrane SNARE proteins (three Ssop) in *Y. lipolytica *compared to the four other yeasts. *S. cerevisiae *and *C. glabrata *have two *SSO *genes whilst in *K. lactis *and *D. hansenii*, we detected only one gene. We finally focused on one specific feature of the *Y. lipolytica *secretory pathway, namely the existence of a Rab4-related protein. In mammalian cells, the GTP binding protein Rab4p is involved in the regulation of plasma membrane protein recycling [16]. A Rab4-related protein is also found in *Schizosaccharomyces pombe *and in filamentous fungi such as *Neurospora crassa*, *Aspergillus fumigatus *or *Phaenerochaete chrysosporium *but is absent from *S. cerevisiae*, *Candida albicans *[17] and the three other hemiascomycetous yeasts. We constructed a strain of *Y. lipolytica *deleted for the *RAB4 *gene and analysed its phenotypic pattern.

## Results and discussion

### Vesicle-mediated protein transport pathways

The only membrane that a secretory protein must traverse is the membrane of the endoplasmic reticulum, the transport of the protein to its final destination continues through vesicles which bud and fuse between organelles [for reviews: [18,19]]. Vesicle budding is mediated by protein coats; vesicle targeting and fusion depend on Rab GTPase, tethering factors and SNARE complexes.

#### Vesicle budding

##### Protein coats (see Additional file 1)

###### Endoplasmic reticulum to Golgi transport

Endoplasmic reticulum to Golgi transport is mediated by the action respectively of the COPII and COPI coat complexes [[20-23] for reviews]. The COPII coat is assembled on the endoplasmic reticulum membrane and allows cargo selection and membrane budding [24]. The COPI complex is involved in retrieval of recycled proteins back to the endoplasmic reticulum [25]. COP I subunits could also have a role in vacuolar sorting [26].

####### COPII coat vesicles (see Additional file 2-1)

Vesicle budding is initiated by the activation of the GTPase Sar1p by the endoplasmic reticulum integral membrane guanine exchange factor Sec12p [27,28]. Sar1p initiates membrane curvature [[29], for mammalian Sar1p see [30]]. The membrane-bound Sar1p-GTP recruits the heterodimer complex Sec23p-Sec24p. These pre-budding complexes are gathered by the Sec13p-Sec31p complex into nascent vesicles [see [31]: the mammalian Sec13p-Sec31p structure]. The Sec23p subunit activates the hydrolysis of GTP by Sar1p and reverses the assembly process. Sec16p stabilizes the coat against premature disassembly after Sar1p hydrolyses GTP [32]. Using fluorescence resonance energy transfer to monitor the assembly and disassembly of COPII coat, it was suggested that a kinetically stable prebudding complex was maintained during multiple Sar1p GTPase cycles [33]. In *S. cerevisiae*, there are one Sec23p, one Sec23p-related protein, one Sec24p, two Sec24p-related proteins (Sfb2p and Sfb3p) and one Sec12p homologue (Sed4p). In *Y. lipolytica *and *D. hansenii*, all the COP II coat components are well conserved and we find two Sec23p-homologues, two Sec24p-homologues but no Sed4p proteins. In *C. glabrata*, there are two Sec23p-homologues, three Sec24p-homologues and two Sec13p-homologues and in *K. lactis *we found the same proteins as in *S. cerevisiae *with the exception of Sfb2p and Sed4p.

####### COP I coat vesicles (see Additional file 2-2)

The COP I coat assembles by the same process as COP II complex involving an Arfp-GTPase [for a review about Arf1p: [34]; mammalian Arf1p: [35]; mammalian COPI assembly review: [36]]. All the *S. cerevisiae *components are conserved in *Y. lipolytica*, though the *Y. lopolytica *Sec28p is only weakly related to the *S. cerevisiae *protein. In *K. lactis*, the Arf1 protein homologue was not identified but another Arf protein could play the role of Arf1p (see Additional file 2-12).

###### Post-Golgi transport

In the trans-Golgi network, the proteins are sorted to the plasma membrane, the endosomal/vacuolar system or recycled back from the endosome. Coated vesicle adaptors facilitate cargo selection [for reviews: [19,37]].

####### Adaptor protein complex (see Additional file 2-3)

In *S. cerevisiae*, by homology to the mammalian adaptor protein (AP) subunit sequences, three potential heterotetrameric adaptor protein complexes have been identified [38]. Each complex is composed of two large (Aplp), one medium (Apmp) and one small (Apsp) subunits. The AP-1 complex is associated with clathrin-coated vesicles and is involved in retention of late Golgi membrane proteins [39] and trafficking to the vacuole [40]. This complex is alone able to associate with clathrin [38,41]. Unlike the mammalian AP-2 complex which associates with endocytic clathrin-coated vesicles, the AP-2 complex of *S. cerevisiae *is apparently not involved in endocytosis. The AP-3 complex is involved in independent clathrin-coated vesicle transport of membrane proteins from Golgi to vacuoles [42]. In *Y. lipolytica*, we also identified three potential AP complexes. As in *S. cerevisiae*, two AP-1 medium subunits were found, but only two small subunits could be identified which could correspond to the AP-2 and AP-3 small subunits. The three other yeasts have the same set of proteins as *S. cerevisiae *for their adaptor protein complexes.

####### GGA proteins (Golgi-localized, γ ear-containing, ARF-binding proteins) (see Additional file 2-3)

GGA proteins are implicated in Golgi to endosomes clathrin-coated vesicle transport and bind to ubiquitin to facilitate this sorting [43,44]. In *S. cerevisiae*, the *GGA *gene is duplicated but in the four yeasts studied, as in *C. elegans *and *D. melanogater*, there is only one gene: *GGA2 *which, evolutionary, is closer to the hypothetical common ancestor [45].

####### Synaptojanin-like protein (see Additional file 2-3)

The *S. cerevisiae *Inp53p, a synaptojanin-like protein acts together with the AP-1 complex in the Golgi to endosome clathrin-dependant pathway which is distinct from the direct Golgi to prevacuolar compartment mediated by GGA proteins [46].

####### Retromer complex (see Additional file 2-4) and sorting nexins (see Additional file 2-5)

Sorting nexins play a role in retrieval of proteins from the prevacuolar compartment or post-Golgi endosomes and different nexins operate in different classes of endosomes [reviewed in [47-49]]. The sorting nexins Snx4p, Snx41p and Snx42p are required for the retrieval of the SNARE Snc1p from the post-Golgi endosome; Grd19p and the retromer complex are involved in the retrieval of endosomal SNARE Pep12p from the prevacuolar compartment [50]. The retromer complex consists of five proteins: Vps5p, Vps17p, two sorting nexins which form a dimer and associate with the complex formed by Vps26p, Vps29p and Vps35p [see [51,52] for mammalian retromer complex structure].

#### Vesicle targeting and fusion

##### RabGTPase (see Additional file 3 and Additional file 2-6)

**Figure 1:**
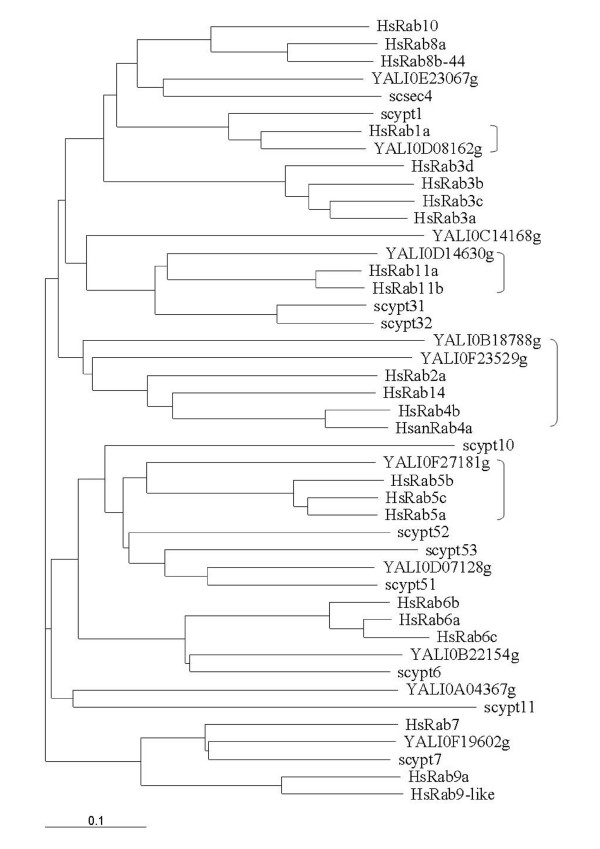
**Phylogenetic tree of some human Rabp, *S. cerevisiae *and *Y. lipolytica *Yptp**. "]" indicates when *Y. lipolytica *protein sequences are closer to human ones. The tree was obtained with ClustalX program, 1.81 version [177] and presented with Treeview program, 1.6.6 version [178].

**Figure 2:**
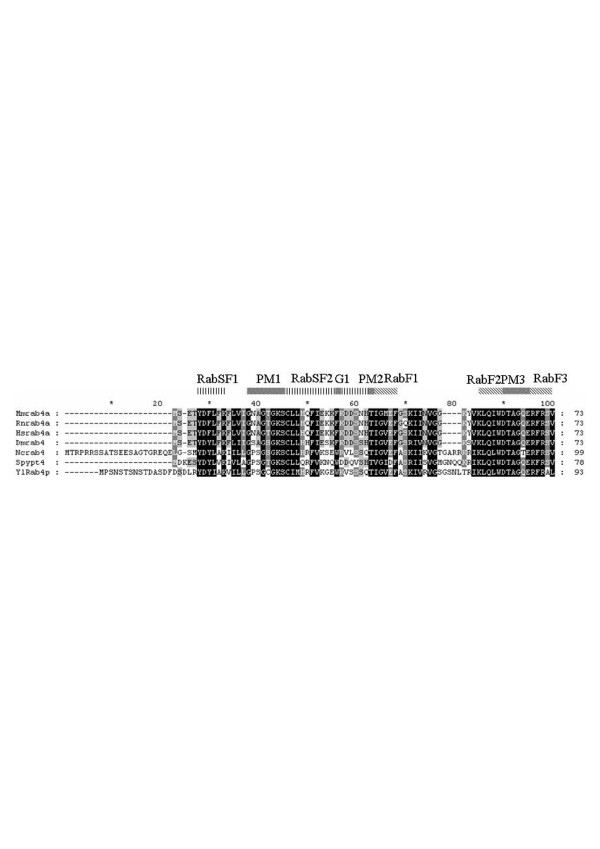
**Ypt4p/Rab4p protein sequences alignment**. The figure shows the upper quartile, for the full image, see Additional file 4. *Mus musculus *(Mm), *Rattus norvegicus *(Rn), *Homo sapiens *(Hs), *Drosophila melanogaster *(Dm), *Neurospora crassa *(Nc), *Schizosaccharomyces pombe *(Sp) and *Yarrowia lipolityca *(Yl) Ypt4/Rab4 protein sequences alignment was obtained with ClustalX program, 1.81 version [177] and presented with GeneDoc program, 2.6.002 version [179].

**Figure 3:**
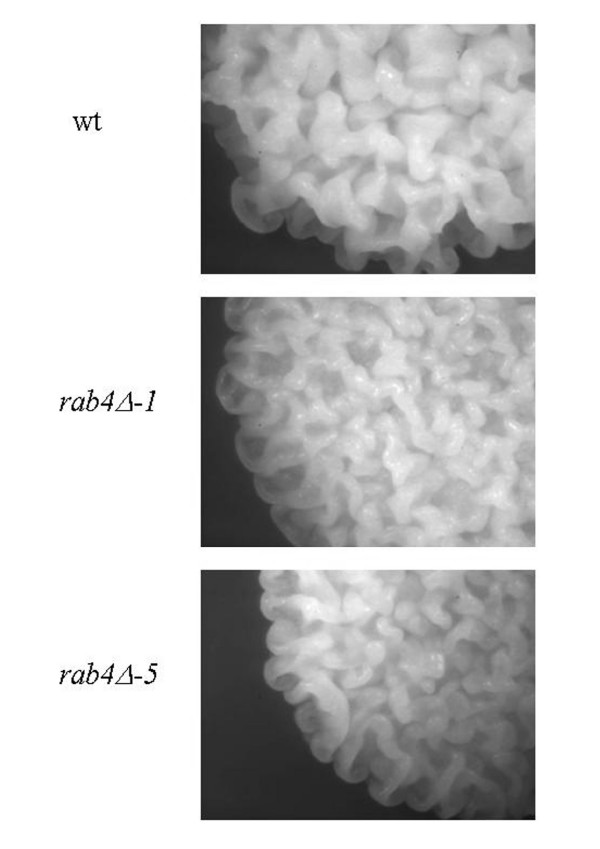
**Colony morphology of *Y. lipolytica *strains**. Wild type (wt) and two independent clones of *rab4Δ *(*-1,-5*) strains were grown as isolated colonies on solid YPD rich medium. Observation (64×) of a five days culture by binocular microscopy.

**Figure 4:**
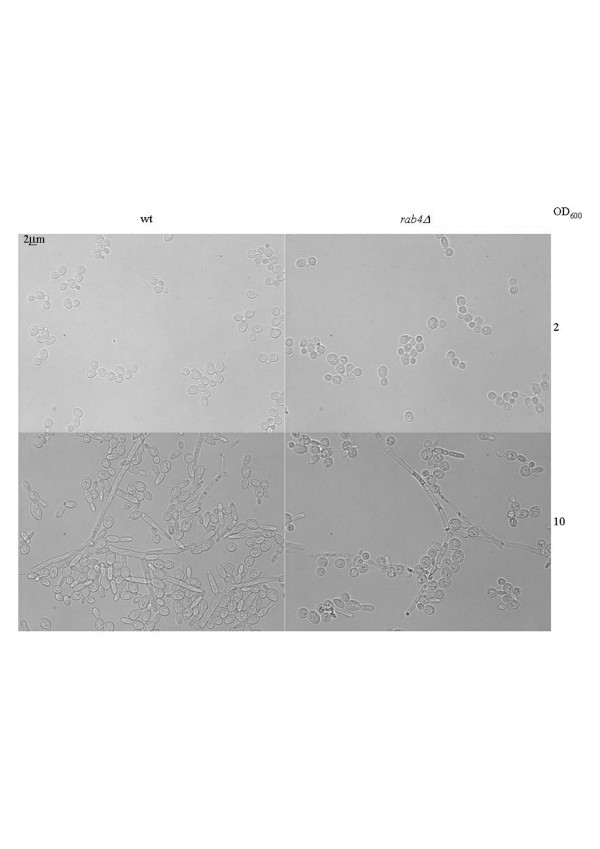
**Disruption of *Y. lipolytica RAB4 *gene does not impair hyphal growth but affects dimorphic transition**. Microscope observation of the wild type (wt) and the mutant (*rab4Δ*) strains in liquid rich YPD medium exponential growth (OD_600_:2) and stationary phase (OD_600_:10).

**Figure 5:**
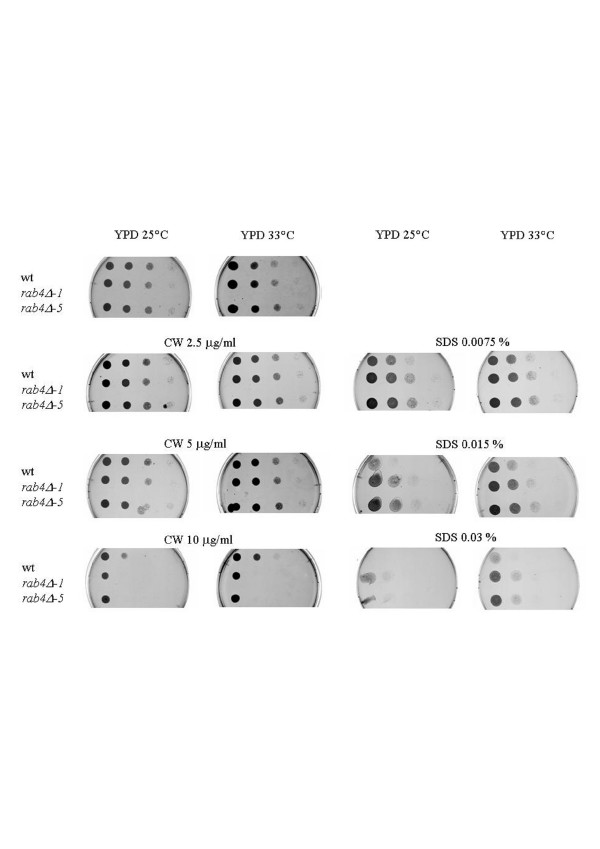
**SDS and Calcofluor white sensitivity**. The *rab4Δ *(*-1,-5*) mutant strains are more sensitive to calcofluor white (CW) and more resistant to SDS than the wild type (wt) strain.

**Figure 6:**
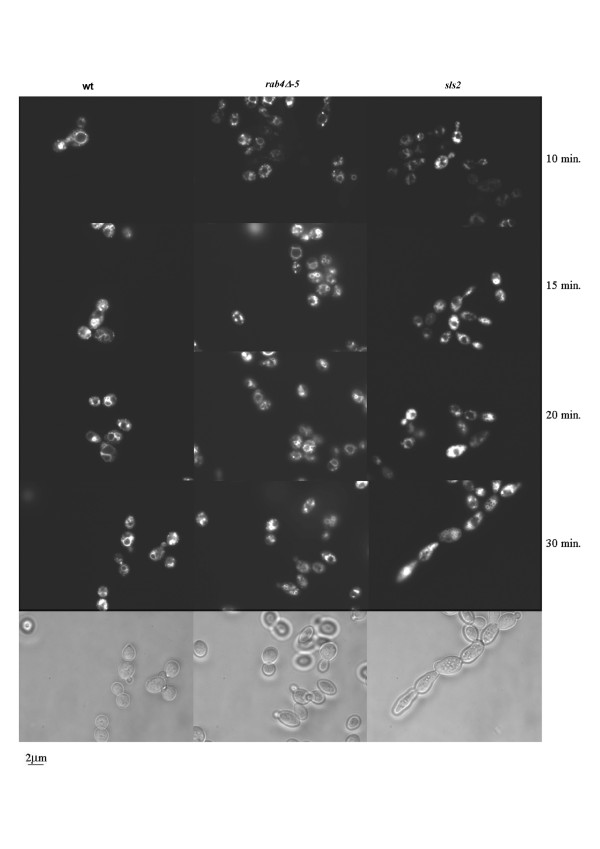
**Endocytosis in the *rab4Δ *mutant strain is not impaired**. The incorporation of FM4-64 in the *rab4Δ-5 *mutant strain is the same as in the wild type (wt) strain compared to a *sls2 *mutant strain in which the FM4-64 incorporation is delayed. *Y. lipolytica *Sls2p is homologous to the *S. cerevisiae *Rcy1p which plays a role in the recycling pathway (see "The vesicle-SNARE Snc1p recycling" section). Low panel: Nomarski.

Rab proteins are small monomeric guanosine triphosphatase (GTPase) which are membrane-associated and cycle between an active GTP-bound state and an inactive GDP-bound protein. These switches regulate all the steps in the secretion pathway. Mammalian Rab proteins belong to the Ras superfamily of GTPase. All the members of this superfamily have conserved nucleotide, phosphate and magnesium binding sequences but the Rab sequences can be distinguish by their C-terminal prenylation site and five Rab-specific regions (RabF) [53]. Pereira-Leal and Seabra [53] have also identified Rab subfamily specific regions (RabSF). They studied the evolution of the Rab family [54] and by their analysis, they observed that Rab proteins co-segregating in the phylogenetic trees showed a pattern of similar cellular localisation and/or function. In *S. cerevisiae*, Ypt1p, Ypt31p/32p and Sec4p are the essential Rab GTPases which regulate the exocytic pathway and Ypt6p, Ypt7p and Ypt51p/52p/53p are involved in the endocytic pathway. *S. cerevisiae *also has two other Rab GTPase, Ypt10p and Ypt11p which are also present in *C. glabrata *and we can find Ypt11p in *K. lactis*. Ypt10p seems to be involved in endocytic function and Ypt11p is required for endoplasmic reticulum inheritance [55]. *In C. glabrata*, a Ypt53p homologue could not be identified and in *K. lactis *it is Ypt32p which was not found. In *Y. lipolytica*, we can find homologues of the nine *S. cerevisiae *proteins necessary for the secretion pathway. Ypt10p and Ypt11p are absent but we can find two other Rab-related proteins, Rab2p and Rab4p as in the filamentous fungi [17]. The analysis of the phylogenic tree (Fig. 1) obtained after alignment of several human Rabp, *S. cerevisiae *and *Y. lipolytica *Yptp sequences revealed that human Rab1p, Rab2p, Rab4p, Rab5p and Rab11p cosegregate with *Y. lipolytica *proteins. As *N. crassa *[56] and other filamentous fungi, *Y. lipolytica *has a large protein secretion capacity and is able to switch from a yeast life cycle to a filamentous form in response to environmental conditions; in this latter form it needs a better capacity of secretion and of recycling plasma membrane material. In mammalian cells, Rab2p has been proposed to regulate the retrograde transport between the Golgi and the endoplasmic reticulum [57] and Rab4p is involved in the recycling of plasma membrane proteins [see [16,58] for a review about recycling pathways]. The comparison of the mouse, rat, human, *N. crassa*, *Schizosaccharomyces pombe *and *Y. lipolytica *sequences (Fig. 2 and Additional file 4) shows that the nucleotide, phosphate and magnesium binding regions, the RabF and RabSF sequences are well conserved. We also compared these Rab4p sequences with other proteins of the mammalian Ras superfamily (data not shown) and we identified a sequence GIQYG next to the RabSF4 region, and particularly the tyrosine residue only present in the Rab4p sequences. The analysis with the NetPhos program (CBS prediction server) identified this tyrosine as a potential phosphorylation site. We suggest that this tyrosine could be important in the regulation of Rab4p activity. In order to get more information about this filamentous fungi specificity, we analysed the effects of a deletion of the gene coding for the *Y. lipolytica *Rab4-related protein. This deletion showed only slight phenotypic changes. The aspect of the colony on rich medium plate was slightly different (Fig. 3). The ability to make the dimorphic transition was not impaired but at OD_600 _of 10, we quantified that the percentage of cells undergoing a dimorphic transition for the wild type strain was 74% and 38% for the mutant strain and the cells in the yeast form had a more spherical appearance in the mutant strain than in the wild type and aggregated more readily (Fig. 4). The round morphological aspect is also observed in the *Y. lipolytica rac *mutant, the Rac protein is another member of the Ras superfamily which is implicated in the induction of the hyphal growth [59]. The slight differences in the morphology of the mutant strains suggested a potential modification of the wall composition. This was confirmed by the increased sensitivity of the strain to calcofluor white (Fig. 5), implicating an increase in chitin composition of the wall. We also observed a decrease in the sensitivity to SDS (Fig. 5) suggesting a decrease in the porosity of the wall. These two events are also encountered when the genes coding for a heterotrimeric G-proteins of *Aspergillus nidulans *are mutated, these mutations in this filamentous fungus confer resistance to the antifungal plant PR-5 (Pathogenesis-Related) protein [60]. We suggest that the *Y. lipolytica *Rab4 protein could be important to recycle the receptor associated with a heterotrimeric G-protein. The mutant *Y. lipolytica rab4 *strains were able to produce diploids as well as the wild type strain (data not shown) indicating that the recycling of the pheromone receptor, associated with a G-protein, was not impaired. The *Y. lipolytica *Rab4p does not regulate endocytosis as the incorporation of FM4-64 was the same as the wild type strain compared to a *sls2 *mutant strain in which the FM4-64 incorporation is delayed (Fig. 6). *Y. lipolytica *Sls2p is homologous to the *S. cerevisiae *Rcy1p which plays a role in the recycling pathway (see "The vesicle-SNARE Snc1p recycling" section below).

###### Regulation of Rab-GTPase [for reviews see [61-64]]

Rab proteins cycle between cytosolic inactive GDP-bound form and active membrane associated GTP-bound form. The cytosolic form exists in a complex with a GDP dissociation inhibitor (GDI). Post-translational prenylation of the protein is important for its activity and prenylated Rabp is recruited to the appropriate membrane by a GDI displacement factor (GDF) which catalyses the dissociation step. The nucleotide exchange is favoured by the guanine nucleotide exchange factor (GEF). GTP-bound Rabp is then activated and can interact with its effectors. The recycling of the Rab protein is stimulated by the GTPase activating protein (GAP) and the GDP-bound Rab protein is released from the membrane by GDI.

####### Prenylation [[65], see [66] for mammalian prenylation] (see Additional file 2-7)

Most of Rab proteins contain two C-terminal cysteine residues which are isoprenylated with two geranylgeranyl moieties. This reaction is catalyzed by geranylgeranyl transferase II (GGTase II), this enzyme has two subunits, a third subunit, Rab escort protein (REP), is a chaperone.

####### GDP dissociation inhibitor (GDI) (see Additional file 2-7)

Gdi1p recycles the Yptp/Sec4p proteins from their target membranes back to the vesicular pool [67].

####### GDI displacement factor (GDF) (see Additional file 2-7)

*In vitro *experiments with mammalian proteins identified that Yip3p catalyses the dissociation of endosomal Rab proteins from GDI [68]. The Yip family (Yip1p, Yip2p, Yip3p, Yip4p, Yip5p and Yif1p) are membrane proteins which interact with prenylated Rab proteins [69]. In *S. cerevisiae*, Yip1p has been identified through a two-hybrid screen as a protein interacting with Ypt1p and Ypt31p in their GDP form [70]. With a similar screen, Yif1p has been identified as a Yip1p-interacting protein [71]. These two proteins form an integral membrane complex that bind Ypt1p and is required for Golgi membrane fusion by interaction with the Golgi SNARE proteins [72]. Yos1p (Yip One Suppressor 1) associates with Yip1p-Yif1p complex [73]. This protein was only identified in *D. hansenii*.

####### Guanine exchange factor (GEF) (see Additional file 2-7)

The activation and membrane stabilisation of the Rab protein are accompanied by exchange of the GDP for the GTP, this activity being catalysed by the guanine exchange factor. Each GEF is specific for a Rab protein and seems to be recruited by the activated Rabp playing a role immediately upstream in the secretion pathway [74,75]. The TRAPP I protein complex binds the COP II vesicles and activates Ypt1p by guanine exchange [76]. TRAPP II Trs120p-Trs130p subunits join the TRAPP I complex to switch the GEF activity from Ypt1p to Ypt31p-Ypt32p acting in late Golgi [77,78]. Sec2p [see [79] for the crystal structure and 80 for the crystal structure of the Sec2p/Sec4p complex] is a highly efficient guanine exchange factor of Sec4p [81], the Rabp essential for exocytosis [[82], see [83] for Sec4p regulation cascade, [84] for Sec2p association with exocyst]. Vps9p is the Ypt51p GEF [85]. The Ric1/Rgp1p is the Ypt6p GEF [86], the BLAST against the *S. cerevisiae *proteins showed only one subunit in *Y. lipolytica*, Rgp1p, but by comparison with the *D. hansenii *protein we could identify a potential *Y. lipolytica *Ric1p. And Vps39p is the Ypt7p GEF [87].

####### GTPase activating protein (GAP) (see Additional file 2-7)

The recycling of the Rab protein is favoured by the GTPase activating protein. In *S. cerevisiae*, eight GTPase activating proteins have been identified and are not specific for one Rab protein in *in vitro *experiments (Gyp1p, Gyp6p, Gyp7p:  [88]; Gyp2p, Gyp3p, Gyp4p:  [89]; Gyp5p, Gyp8p:  [90]). In the four yeasts, the Gyp3p homologue, Gyp4p was not identified. Gyl1p is Gyp-like protein interacting with Gyp5p involved in the control of polarized exocytosis [91], this protein has an homologue only in *C. glabrata *but not in the three other yeasts.

##### Tethering factors (see Additional file 5), [reviewed in [92-94]]

The secretory vesicles are tethered to their target membrane by two classes of molecules: coiled-coil proteins able to form homodimeric complex as long as several times the diameter of the vesicle and large multisubunit complexes.

###### Endoplasmic reticulum-cis-Golgi-network

Several factors are involved in the tethering of vesicles to the Golgi, TRAPP complex (see Additional file 2-8), COG complex (see Additional file 2-9) and Uso1p (see Additional file 2-10). TRAPP is associated with the Golgi and two forms of the complex exist: TRAPP I (7 subunits) acts in the endoplasmic reticulum to Golgi transport and TRAPP II which contains the TRAPP I subunits together with three other proteins acts in Golgi traffic. In *Y. lipolytica *only two TRAPP II specific subunits were identified by comparison with the *S. cerevisiae *protein sequences but for the Trs65p we used the protein identified in *D. hansenii *to detect a potential *Y. lipolytica *protein. Both complexes are able to interchange guanine nucleotide on Ypt1p. *In vitro*, TRAPP I can bind COPII vesicles by binding the coat Sec23p subunit [95] and this could be the first event before interaction of the vesicle with its target [76]. The crystal structure of the mammalian Bet3p, the most conserved TRAPP protein, reveals a dimeric structure with hydrophobic channels and a covalent modification with a palmitate [96,97], the crystallographic study of the complex Bet3p-Trs33p reveals specific interactions between these subunits [98]. This subunit could be responsible for the targeting and the anchoring in the Golgi membrane and could direct the other TRAPP components to the Golgi [96,99]. Trs120p, a TRAPP II subunit, is required for vesicle traffic from the early endosome to the late Golgi [100]. Trs120p and Trs130p TRAPP II subunits are conserved from yeast to mammals; the Trs65p subunit is conserved only in some fungi and unicellular eukaryotes [101]. The other tethering factors, Uso1p, a long coiled-coil protein and the COG complex composed of eight subunits in *S. cerevisiae *are recruited before the last step of membrane fusion. Uso1p and the COG complex also have a function in sorting of endoplasmic reticulum-vesicles containing GPI-anchored proteins [see [102] for a review about differential ER exit] and in retrograde vesicular trafficking within the Golgi [reviewed in [103]]. The Uso1 protein consists of an N-terminal globular head region, a coiled-coil tail which mediates dimerisation and a C-terminal acidic region. The NCBI Conserved Domain Architecture Retrieval Tool has identified in the *Y. lipolytica *Uso1 protein the first two domains but not the C-terminal acidic region. When we compare the consensus sequence of this region with the *Y. lipolytica *sequence, only the last 50 residues of this domain are well conserved. The *Saccharomyces cerecisiae *and the mammalian COG complex are composed of eight subunits, a multiple of four subunits, as one the GARP and the exocyst complexes (see below), which could reflect an interaction with a four-component complex such as the trans SNARE complex [92]. In *Y. lipolytica*, only five subunits were identified as probable COG proteins by comparison with the *S. cerevisiae *proteins. Cog1p, Cog2p and Cog7p were not detected by BLAST searches but in mammals these proteins were identified by their function as their sequence similarity with the *S. cerevisiae *proteins is low [104]. Cog2p was found in *D. hansenii *and its sequence used to make the BLAST search with the *Y. lipolytica *proteins. This allowed the identification of a potential *Y. lipolytica *Cog2p homologue, but Cog1p and Cog7p were not found in *Y. lipolytica*, and in *D. hansenii*, Cog1p was not found. These proteins probably exist but should be identified by another means.

###### Cis-Golgi-network-Endoplasmic reticulum (see Additional file 2-11)

Dsl1p complex is a large complex composed of the peripheral endoplasmic reticulum membrane proteins Dsl1p, Dsl3p (Sec39p) and Tip20p [105,106]. Dsl1p contains three domains, an N-terminal coiled-coil region of 200 aminoacids which interacts with Tip20p, a central highly acidic region of interaction with Ret2p and Ret1p (two COP I subunits) and a conserved C-terminal sequence which could recruit cytoskeletal elements [105]. The 200 amino acid N-terminus from the Dsl1p protein identified in *Y. lipolytica *does not align with the *S. cerevisiae *sequence. Nevertheless the *Y. lipolytica *sequence also contains potential coiled-coil regions (as determined by the coiled-coil prediction program, NPS@:Network Protein Sequence Analysis, [107]). The Tip20p sequence of *Y. lipolytica *has only 19 % identities with the *S. cerevisiae *sequence. This could explain the divergent N-terminal sequence of Dsl1p which is involved in the interaction of the two proteins. Dsl1p, Tip20p and Dsl3p are required for the stability of the SNARE complex at the endoplasmic reticulum [106].

###### Golgi

*S. cerevisiae *TRAPP II complex (see Additional file 2-8) is composed of ten subunits and could have a role in retrograde transport of Golgi vesicles [108]. The trs130 mutant (coding for a TRAPP II subunit) displays synthetic interaction with mutation in a COPI subunit (Ret2p) and a deletion of ARF1 (see Additional file 2-12) is implicated in COPI formation [108].

A role of the COG complex (see above) has also been found in retrograde transport to early Golgi vesicles [109].

The VFT/GARP complex (see Additional file 2-13) localizes to the trans-Golgi network and is required for retrograde traffic from early endosomes to the Golgi [110,111]. The *S. cerevisiae *complex is composed of four subunits [112,113], it is the effector of Ypt6p and interacts with the SNARE Tlg1p. Only three subunits were identified in *Y. lipolytica *and *D. hansenii *but the undetected Vps51p unit also has no homologue in mammalian [114] and seems to be a regulatory subunit [97] which could be replaced by another protein as suggested by Liewen, *et al*. [114]. The structural analysis of the interaction between *S. cerevisiae *Tlg1p and Vps51p has determined an N-terminal peptide of Vps51p which deletion does not block transport to the late Golgi from endosomes [115].

Golgins (see Additional file 2-10, -12) [reviewed in [116,117]] are coiled-coil proteins which organize the structure and the trafficking pathways in the Golgi. These proteins have mainly been studied in mammalian cells but in *Sacharomyces cerevisiae *several homologues have been identified: Uso1p is the homologue of the mammalian p115 required for endoplasmic reticulum-to-Golgi transport; Grh1p, the GRASP65-homologue, a Golgi localized protein component of the spindle assembly checkpoint [118]; Imh1p, involved in transport between an endosomal compartment and the Golgi, Imh1p contains a Golgi-localization (GRIP) domain that interacts with activated Arl1p-GTP to be localized to the Golgi, this is regulated by Arl3p [[119,120]] and Arl3p requires the N-terminal acetyltransferase NatC complex and the protein Sys1p to be targeted to the Golgi [121] [for a review about Arl proteins see [122]]; Coy1p, the CASP homologue, a Golgi membrane protein related to Giantin, its deletion in *S. cerevisiae *restores normal growth to cells lacking the SNARE Gos1p [123] and Rud3p, a golgin-160-related protein, is a Golgi matrix protein that is involved in the structural organization of the cis-Golgi [124].

###### Golgi-Endosome, Endosome-Vacuole [37]

The TRAPP II subunit Trs120p is required for vesicle traffic from early endosome to the late Golgi [100].

The Vps Class C/HOPS complex [reviewed in: [92,125]) (see Additional file 2-14):

The HOPS complex composed of Vps11p, Vps18p, Vps16p, Vps41p with the protein Vps19p are the effectors of Ypt51p in endosomal traffic. In vacuolar transport, the complex seems to recruit the Rab Ypt7p GEF Vps39p which activates Ypt7p. Activated Ypt7p acts on the HOPS complex to promote tethering and binding to the SNARE Vam3p through the interaction with the SNARE-binding protein, Vps33p. Vps33p together with other HOPS complex subunits is found in complex with Vps8p, a hydrophilic membrane-associated protein [126]. HOPS complex binds phosphoinositides and SNARE Vam7p [127].

###### Golgi-Plasma Membrane

####### The Exocyst (see Additional file 2-15, -16)

The *S. cerevisiae *exocyst complex is composed of eight subunits (Sec3p, Sec5p, Sec6p, Sec8p, Sec10p, Sec15p, Exo70p and Exo84p), a quatrefoil complex as in the COG and the GARP complexes [92]. The activated Rab protein, Sec4p, present on the secretory vesicles, binds the exocyst subunit Sec15p in subcomplex with Sec10p resulting in the association with the other subunits and Sec3p [128]. Sec3p is the spatial landmark defining the sites of polarized exocytosis [129]. The localization of Sec3p is mediated by Rho GTPases, Rho1p [130] and Cdc42p [131]. Rho3p plays a role in exocytosis through its interaction with Exo70p ([132-134]). These Rho proteins also have a role in actin polymerisation. Assembly of the exocyst occurs when the subcomplex associated with the vesicles joins Sec3p and Exo70p on the plasma membrane [135]. The Sec6p subunit dimerizes and interacts with the SNARE Sec9p, playing a role in SNARE complex regulation [136]. A cyclical regulatory network contributes to the establishment and maintenance of polarized cell growth [137]. Bem1p interacts with Sec15p and is involved in the Cdc42p-mediated polarity [138].

##### SNARE (Soluble N-ethylmaleimide-sensitive factor Attachment protein REceptor) proteins [see reviews: [125,17,139-141] for mammalian SNAREs] (see Additional file 6 and Additional file 2-17)

After the tethering of the vesicle close to its target membrane, the fusion of the membranes is initiated through the action of SM (Sec1/Munc18, see below) and SNARE proteins. SNARE proteins share one conserved sequence called the SNARE motif which contains 60–70 amino acids that include heptads repeat typical of coiled coils. They contain a C-terminal transmembrane domain or a hydrophobic post-translational modification motif. SNARE proteins associate to form complex undergoing conformational changes. Free SNARE motifs are unstructured and when they are associated in a complex they assemble into elongated four-helical bundles. SNAREs are present on the vesicle and the target membranes and the formation of the complex pulls the membrane close together. SNARE proteins are classified in to subfamilies based on a highly conserved layer of interacting amino acids (three glutamines: Qa-, Qb-, Qc-, one arginine: R-) in the centre of the helix bundle. All complexes contain one copy of each SNARE motif. In *S. cerevisiae*, twenty-four SNARE-encoding genes have been identified [reviewed in [139]]. Twenty-two of these genes could be found in *Y. lipolytica *by sequence homology. As in other fungi [17], Vam3p and Spo20p were not detected. Spo20p, which seems specific to *S. cerevisiae *(also not identified in the three other yeasts), contains both Qb and Qc SNARE motifs and is required during sporulation for the prospore membrane formation [142,143]. Vam3p SNARE is required for homotypic vacuole fusion in *S. cerevisiae *and there is no homologue outside the Saccharomycetes, the Saccharomycetaceae, *K. lactis*, has a Vam3p SNARE, but in *D. hansenii*, another Saccharomycetaceae as in *Y. lipolytica *(Dipodascaceae) the Vam3 protein was not detected. The mitosporic Saccharomycetales, *C. glabrata*, possess a Vam3 protein. Pep12p is a late endosomal Qa SNARE with sequence similarity with Vam3p, that can complement *vam3 *mutants [144]. In *Y. lipolytica *and in *D. hansenii*, the BLAST searches allowed us to identify the Pep12p SNARE and another SNARE we propose to name Pep12p-like because its sequence is closer to Pep12p than to Vamp3p. This Pep12p-like SNARE could play the role of the *S. cerevisiae *Vam3p in both yeasts. A specificity of *Y. lipolytica *is the presence of three *SSO *genes resulting probably from gene triplication, and this seems unique to *Y. lipolytica*. The Sso proteins are implicated in the fusion of the secretory vesicles to the plasma membrane and *S. cerevisiae *Sso1p also has sporulation-specific functions [145]. The multiplicity of *SSO *genes in *Y. lipolytica *could reflect its good secretion capacity and its capacity to induce hyphal growth which needs better recycling of plasma membrane material. On the contrary, only one Sso protein (Sso2p) and one Snc protein (Snc2p) were found in *K. lactis *and *D. hansenii*. The *SSO *and *SNC *genes of *K. lactis *have been cloned by complementation in *S. cerevisiae *of *sso2-1 *and *snc1Δ snc2Δ sem1Δ *mutant strains. The *K. lactis *Ssop can perform both of the *S. cerevisiae *Ssop functions and the *K. lactis *Sncp seems to perform only the *S. cerevisiae *Sncp functions [146]. Sft1p was not detected in *C. glabrata*, Syn8p was not detected in *C. glabrata *and *K. lactis *and Nyv1p was not detected in *D. hansenii*.

###### Regulation of fusion

####### The Sec1/Munc18 (SM) proteins (see Additional file 2-18) [reviewed in [147]]

The SM proteins confer specificity to membrane fusion through their binding to the N-terminal domain of the Qa SNARE protein. Four SM proteins have been found in *S. cerevisiae*, Sly1p acting between the endoplasmic reticulum and Golgi [148] and Sec1 at the final step of exocytosis, Vps45p and Vps33p playing a role between Golgi and endosomes and between endosomes and vacuole. Each SM protein can bind several Qa SNARE but also other SNARE implicated in the same complex, as has been shown for Sly1p [149]. In addition one SNARE can bind two SM proteins at the same organelle: Vps45p and Vps33p with Pep12p [126]. Mso1p, a Sec1p-interacting protein, binds to SNARE complex and plays an essential role for vesicle fusion during prospore membrane formation [150,151].

####### Vsm1p (see Additional file 2-19)

The phosphorylation of SNAREs by the cAMP-dependent protein kinase (PKA) regulates their ability to assemble into functional complexes [152,153]. Phosphorylation of the Sed5p t-SNARE regulates endoplasmic reticulum-Golgi transport as well as Golgi morphology [154]. The Ssop phosphorylation allows the binding of Vsm1p, a negative regulator of secretion which prevents the formation of the SNARE complex [155]. Dephosphorylation of Ssop by ceramide activated protein phosphatase (CAPP) increases its ability to form a complex with Sec9p [152]. Three *S. cerevisiae *genes code for a PKA but only one gene could be identified in *Y. lipolytica *and *D. hansenii *and two were identified in *C. glabrata *and *K. lactis*. The CAPP is composed of two regulatory subunits (Tpd3p and Cdc55p) and one catalytic subunit (Sit4p) which were found by sequence homology in the four yeasts in addition to further one Tpd3p-like in *C. glabrata*.

####### SNARE recycling

SNARE complex dissociation (see Additional file 2-20):

After the membrane fusion step, the trans-SNARE complex becomes a cis-SNARE complex whose dissociation requires the ATPase Sec18p and the soluble NSF-attachment protein (Sec17p) as cofactor [156,157]. In vacuole fusion, Sec17p may displace HOPS from SNAREs to permit subsequent rounds of fusion [158]. Two Sec18 proteins were detected in *C. glabrata*.

####### The vesicle-SNARE Snc1p recycling (see Additional file 2-20)

The *S. cerevisiae RCY1 *gene has been identified with a screen for mutants affected in membrane traficking along the endocytic pathway [159]. Rcy1p contains an amino-terminal F box and a CAAX box motif in its carboxyl-terminal sequence. The F box region of the protein is required for the recycling of the vesicle-SNARE Snc1p. The CAAX box is required for its localization in polar growth regions. Rcy1p interacts with Skp1p through the F box motif and both proteins form a complex necessary for the recycling function [160]. Rcy1p is a positive regulator of Ypt6p [161]. Gyp1p, the Ypt1p GTPase activator is also involved in the recycling of Snc1p [162], as well as Snx4p, Snx41p and Snx42p [43]. The ARF-GAP Gcs1p facilitates the incorporation of the Snc1p into COPI recycling vesicles [163]. Rcy1p is a downstream effector of Ypt31, 32p [164].

In *Schizosaccharomyces pombe*, Pof6p, the Rcy1p-homologue, has been identified through a two-hybrid interaction with Skp1. Both proteins are required for normal septum processing and cell separation [165], a function which may also require the exocyst function [166].

Previous to these works, a mutation *sls2-1*, was isolated in *Y. lipolytica *that causes synthetic lethality when combined with the conditional lethal mutation in the 7S RNA of the signal recognition particle [167]. Rcy1p and Pof6p are the homologues of Sls2p.

## Conclusion

**Figure 7:**
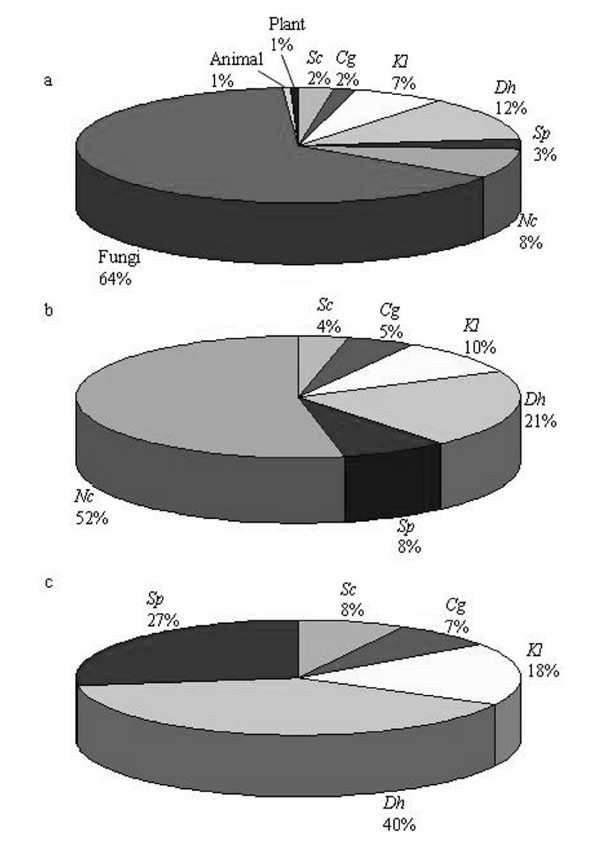
**The percentage of *Y. lipolytica *proteins with the greatest homology: **a: to *Saccharomyces cerevisiae*, *Candida glabrata*, *Kluyveromyces lactis*, *Debaryomyces hansenii*, *Schizosaccharomyces pombe*, *Neurospora crassa*, other fungi, animals, plants proteins; b: to *Saccharomyces cerevisiae*, *Candida glabrata*, *Kluyveromyces lactis*, *Debaryomyces hansenii*, *Schizosaccharomyces pombe*, *Neurospora crassa *proteins; c: to *Saccharomyces cerevisiae*, *Candida glabrata*, *Kluyveromyces lactis*, *Debaryomyces hansenii*, *Schizosaccharomyces pombe *proteins. See Additional file 7 for the list of E-values obtained with BLAST of *Y. lipolytica *proteins against NCBI eukaryotic sequences.

**Figure 8:**
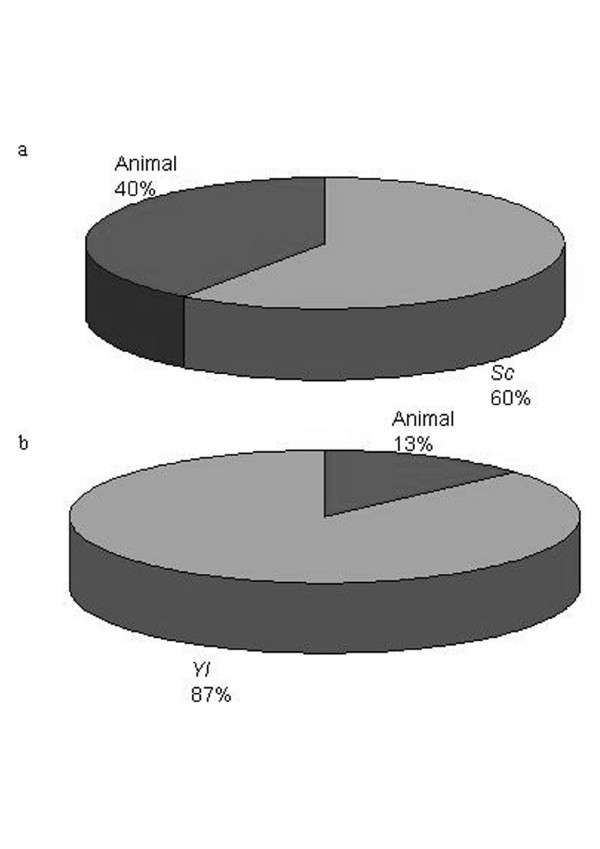
**Percentages of greatest homology**. a: The percentage of *Y. lipolytica *proteins with the greatest homology to *S. cerevisiae *and animal proteins; b: The percentage of *S. cerevisiae *proteins with the greatest homology to *Y. lipolytica *and animal proteins. See Additional file 8 for the list of E-values obtained with BLAST of the *S. cerevisiae *proteins against NCBI eukaryotic protein sequences.

The sequencing of four hemiascomycetous yeasts has allowed us to search for proteins involved in vesicular transport by comparison with proteins identified in *S. cerevisiae*. The method used does not allow the identification of a protein which does not exist in *S. cerevisiae *or does not belong to a protein family. To identify new candidates, a list from other organism should be established or experimental approach should be performed. The proteins identified are highly conserved between the five yeasts but we have brought to light several specificities of *Y. lipolytica *in keeping with its good protein secretion capacities and its dimorphic aspect. In Table 1, we summarize these differences. Some of the proteins for which we did not find homologues have probably a too divergent sequence to be identified through the BLAST searches and may be identified in the future by functional screens. But, the presence of Rab2p- and Rab4p-related proteins as is found in fungi, a potential role of Rab2-related protein and Ypt1p in vesicular transport between endoplasmic reticulum and Golgi and of Rab4-related protein in addition to Sls2p/Rcy1p in membrane recycling reflect the greater complexity of the *Y. lipolytica *secretion pathway, which is probably dictated by the necessity to secrete and recycle membrane material needed for its filamentous growth. In this work, we have shown that the Rab4p-related protein could have a role in this membrane recycling since a modification of the aspect of the colony, the decrease of the number of cells undergoing dimorphic transition and the change of the wall permeability of the *rab4 *deleted strain were observed. The three Sso proteins are also indicative of a large secretion capacity and a study of this specificity would be interesting. As previously shown through the diverse studies of *Y. lipolytica*, this dimorphic yeast has a secretion pathway closer to the mammalian one than has *S. cerevisiae*. Its translocation apparatus is largely devoted to the co-translational translocation of nascent peptides through the endoplasmic reticulum membrane as is seen in mammalian cells. *C. glabrata *and *K. lactis *are the closest to *S. cerevisiae *for the proteins listed. We found among the *C. glabrata *proteins, 2 COPII coat protein Sec13p-homologues and 2 Sec18p-homologues implicated in the SNARE recycling and this seems to be specific to this yeast. For *K. lactis *and *D. hansenii*, only one Ssop and one Sncp were detected. Though *D. hansenii *is classified in Saccharomycetaceae, as is *S. cerevisiae*, and *Y. lipolytica *is a Dipodascacae, these two yeasts seem closer at least for the proteins involved in vesicular transport. These results are in agreement with the phylogenetic tree presented by Dujon *et al *[11]. In Fig. 7a, we have shown the proportion of the 165 *Y. lipolytica *protein sequences listed (see Additionnal file 2: *Y. lipolytica *column) with the greatest homology to *S. cerevisiae*, *C. glabrata*, *K. lactis*, *D. hansenii*, *Schizosaccharomyces pombe*, *N. crassa*, other fungi, animals and plants sequences (see Additional file 7). 72% of these proteins are closest to fungi and *N. crassa*. If we exclude animals, plants and fungi (keeping only *N. crassa*) from this search (Fig. 7b), we observed that 52% of the *Y. lipolytica *proteins listed are closer to *N. crassa*. The last comparison is performed without *N. crassa *(Fig. 7c): in this case, 40% of the proteins are closer to *D. hansenii*. This could reflect their common physiology, the filamentous growth and good secretion ability for *Y. lipolytica *and *N. crassa *and their proteolytic and lipolytic activities and high salt tolerance [168] for *Y. lipolytica *and *D. hansenii*. These results confirm that considering the identified proteins playing a role in vesicular transport, *Y. lipolytica *is closer to the fungi than to *S. cerevisiae*. The proteins that are the best conserved between *S. cerevisiae *and *Y. lipolytica*, are the SNARE proteins (see Additional file 7). In Fig. 8a, we have presented the percentage of the *Y. lipolytica *proteins with the greatest homology to the *S. cerevisiae *and animal: 40% of these proteins are closer to the animal ones, particularly, proteins of the AP complexes, Ypt and Arf proteins, TRAPP and HOPS complexes (see Additional file 7), whereas for *S. cerevisiae*, only 13% of these proteins are closer to animals than to *Y. lipolytica *(Fig. 8b, see Additional file 8). Koumandou and coworkers [169] have analysed the protein sequences of tethering complexes and SM proteins from five eukaryotic supergroups. They conclude that the most recent common eukaryotic ancestor had a complex endomembrane system with COG, Exocyst, Dsl1, GARP tethering complexes which could have originated from one common ancestral complex, TRAPP and HOPS complexes which are independently derived and all four SM protein families represented. The phylogenetic tree presented by Dujon *et al *[11] indicates that *Y. lipolytica *may be less distant from the last common eukaryotic ancestor and the observation of a good sequence conservation between *Y. lipolytica *and animal TRAPP and HOPS subunits for example, suggests that these complexes are required for the evolution of multicellular organisms. Similarly, Hall *et al. *[170] showed that Rab4p was an ancient component of the endomembrane trafficking system since it exists, and its recycling function is conserved, in *Trypanosoma brucei *which belongs to an eukaryotic supergroup separated from that of yeast, fungi and animals. In *Y. lipolytica *also, the presence and the possible recycling role of a Rab4-like protein was observed while in *S. cerevisiae *and in the three other hemiascomycetous yeasts, this protein has been lost. These observations indicate that *S. cerevisiae *has diverged further from the last common eukaryotic ancestor than has *Y. lipolytica*, as far as vesicle-mediated protein transport pathways are concerned and that *Y. lipolytica *has retained the complexity of the trafficking system allowing evolution to a multicellular organization. So as has been said for fungi [171], we can say that «*Yarrowia lipolytica *and humans are closer than you think» and that this yeast constitutes an interesting model to study the secretion pathway.

**Table 1:** Differences observed for the five hemiascomycetous yeasts.

	*S. cerevisiae*	*Y. lipolytica*	*C. glabrata*	*K. lactis*	*D. hansenii*
COPII	Sec24p, Sfb2,3p (Sec24p-related)	2 proteins	3 proteins	2 proteins	2 proteins
	Sec13p	1 protein	2 proteins	1 protein	1 protein
	Sed4p	no hits	1 protein	no hits	no hits
Adaptor	Aps1,2,3p	2 proteins	3 proteins	3 proteins	3 proteins
	Gga1,2p	1 protein (Gga2p)	1 protein (Gga2p)	1 protein (Gga2p)	1 protein (Gga2p)
Sorting Nexin	Snx41,42p	1 protein	2 proteins	2 proteins	2 proteins
Yptp	Ypt7p	1 protein	1 protein	1 protein	2 proteins
	Ypt10p	no hits	1 protein	no hits	no hits
	Ypt11p	no hits	1 protein	1 protein	no hits
	Ypt31-32p	2 proteins	2 proteins	1 protein (Ypt31p)	1 protein (Ypt32p)
	Ypt51,52,53p	3 proteins	2 proteins	3 proteins	3 proteins
	no hits	Rab2,4p-related	no hits	no hits	no hits
Yptp regulation	Yos1p	no hits	no hits	no hits	1 protein
	Gyp4p	no hits	no hits	no hits	no hits
	Gyl1p	no hits	1 protein	no hits	no hits
COG complex	Cog1p	no hits	1 protein	1 protein	no hits
	Cog2p	1 protein	1 protein	1 protein	1 protein
	Cog7p	no hits	1 protein	1 protein	1 protein
Arfp	Arf1,2,3p	2 proteins(Arf1,3p)	2 proteins(Arf1,2p)	2 proteins (Arf2,3p)	3 proteins
	Arl1p	2 proteins	1 protein	1 protein	1 protein
Arl3p localization	NatC complex (3 proteins)	2 proteins	3 proteins	3 proteins	3 proteins
GARP complex	Vps51p	no hits	1 protein	1 protein	no hits
SNARE-Qa	Vam3p	Pep12p-like	1 protein	1 protein	Pep12p-like
	Sso1,2p	3 proteins	2 proteins	1 protein (Sso2p)	1 protein (Sso2p)
SNARE-Qb,Qc	Spo20p	no hits	no hits	no hits	no hits
SNARE-Qc	Sft1p	1 protein	no hits	1 protein	1 protein
	Syn8p	1 protein	no hits	no hits	1 protein
SNARE-R	Nyv1p	1 protein	1 protein	1 protein	no hits
	Snc1,2p	2 proteins	2 proteins	1 protein (Snc2p)	1 protein (Snc2p)
Exocytosis SNARE regulation proteins	Tpd3p (CAPP regulatory subunit)	1 protein	2 proteins	1 protein	1 protein
	Tpk1,2,3p (PKA)	1 protein	2 proteins	2 proteins	1 protein
SNARE recycling	Sec18p	1 protein	2 proteins	1 protein	1 protein

See Additional file 2 for the list of proteins potentially implicated in vesicular transport.

## Methods

### Strains and growth conditions

*Escherichia coli *strains DH5alpha (F'/*end*A1 *hsd*R17 (r_K_^- ^m_K_^+^) *sup*E44 *thi*-1 *rec*A1 *gyr*A (Nal^r^) *rel*A1 Δ (*lac*IZYA-*arg*F) *U*169*deo*R (φ 80*dlac*Δ (*lac*Z)M15) was used as host strain for bacterial transformations and plasmid propagation.

The *Yarrowia lipolytica *strain INAG136463 (*MatB*, *scr1 *: : *ADE1*, *SCR2*, *his-1*, *leu-2*, *ura3*) was used for the inactivation of *RAB4*-related gene.

*Escherichia coli *cells were grown in LB medium (1% bactotryptone, 1% yeast extract, 0.5% NaCl), 100 μg/ml ampicillin, 37°C. *Yarrowia lipolytica *cells were cultivated either on rich YPD medium (1% yeast extract, 1% bactopeptone, 1% glucose), 28°C, or on minimal medium: 0.67% yeast nitrogen base without amino acids (Difco laboratories), 2% glucose as carbon source, 50 mM phosphate buffer pH 6.8, 28°C with amino acids required and 1,25 mg/ml 5'-fluoroorotic acid for *ura3*^- ^strain selection.

### Gene inactivation

Disruption was performed using the two-step «pop-in/pop-out» method [172]. The disrupted gene was obtained by the deletion of the *Bst*EII-*Cla*I fragment of the *RAB4*-related gene cloned between the *Hind*III-*Kpn*I sites of the p0 vector [173].

### DNA techniques

Standard techniques were used according to Sambrook *et al*. [174]. Enzymes were supplied by New England Biolabs. All vectors inserts were checked by sequencing by Genome express (France).

### Transformation procedures

The *E. coli *strains were transformed by the method of Chung and Miller [175].*Y. lipolytica *strain transformations were carried out according to Xuan *et al*. [176].

### Sensitivity to SDS and Calcofluor White

Cells of the INAG136463 (wt) and two clones (1, 5) of the deleted *rab4*-related strains were grown in YPD medium. 5 μl droplets of serial dilutions of exponential growing cultures of each strain were inoculated on the surface of YPD plates containing 2.5 μg/ml, 5 μg/ml, 10 μg/ml Calcofluor White (CW) or 0.0075 %, 0.015%, 0.03% sodium dodecyl sulfate (SDS).

### FM4-64 staining

For the strains of yeast cells, 3 OD_600 _units of exponential growth in YPD medium (OD_600 _0.5–1) were resuspended in 150 μl of YPD containing 40 μM FM4-64. Cells were incubated 10 min. at 28°C and washed three times in ice-cold medium. Cells were resuspended in YPD and incubated at 28°C. Aliquots were taken at various times and internalization was stopped with 10 mM NaN_3 _and 10 mM NaF. Stained cells were visualized using fluorescence optics [adapted from [159]].

### Informatic analyses

Hemiascomycetous yeast genome sequences, BLAST searches of vesicular secretion proteins and BLAST results (performed Apr 25, 2003 with 1,093,702 sequences) were obtained from the Génolevures web site [180]. *S. cerevisiae *sequences were collected from *Saccharomyces *Genome Database [181]. BLASTs against protein databases were obtained from NCBI (BLAST with 4,554,902 sequences) [182] and Infobiogen web site [183]. Protein analyses were done with NCBI Conserved Domain Architecture Retrieval Tool [184], ExPASy Proteomics tools [185] and CBS Prediction Servers [186].

A list of proteins implicated in *S. cerevisiae *vesicular secretion was made from literature. These *S. cerevisiae *protein sequences were used for BLAST searches with the Génolevures web site. For protein families such as Rab protein, autoBLAST, which means BLAST of a sequence against its own genome, were made to identify all the members of the family. The protein sequences of the new members were identified by BLAST searches against the NCBI eukaryotic protein sequences.

The percentages of proteins with the greatest homology (Fig. 7 and 8) were determined by quantification of the best E-values obtained with the BLAST searches against the NCBI eukaryotic protein sequences.

## Abbreviations

*Y. lipolytica*,*Yl*: *Yarrowia lipolytica*; *C. glabrata*, *Cg*: *Candida glabrata*; *K. lactis*, *Kl*: *Kluyveromyces lactis*; *D. hansenii*, *Dl*: *Debaryomyces hansenii*; *S. cerevisiae*, *Sc*: *Saccharomyces cerevisiae*; *N. crassa*, *Nc*: *Neurospora crassa*; SNARE: Soluble N-ethylmaleimide-sensitive factor Attachment protein Receptor.

## Authors' contributions

DS conceived the study, carried out the molecular genetic studies, the sequence analyses and drafted the manuscript. JMB participated in the sequence analyses. All authors read and approved the final manuscript.

## Supplementary Material

Additional file 1 **Drawing of *Yarrowia lipolytica *identified proteins coats**. PM: plasma membrane, ER: endoplasmic reticulum, RE: recycling endosome, EE: early endosome, LE: late endosome, MVB: multi-vesicular bodies, SV: secretory vesicle. Click here for file

Additional file 3 **Drawing of *Yarrowia lipolytica *identified Ypt/Rab GTPases**. PM: plasma membrane, ER: endoplasmic reticulum, RE: recycling endosome, EE: early endosome, LE: late endosome, MVB: multi-vesicular bodies, SV: secretory vesicle. Click here for file

Additional file 4 Full image of Figure 2 Click here for file

Additional file 5 **Drawing of *Yarrowia lipolytica *identified tethering factors**. PM: plasma membrane, ER: endoplasmic reticulum, RE: recycling endosome, EE: early endosome, LE: late endosome, MVB: multi-vesicular bodies, SV: secretory vesicle. Click here for file

Additional file 6 **Drawing of Yarrowia lipolytica identified SNARE and SM proteins**. PM: plasma membrane, ER: endoplasmic reticulum, RE: recycling endosome, EE: early endosome, LE: late endosome, MVB: multi-vesicular bodies, SV: secretory vesicle. Click here for file

Additional file 7 **E-values**. E-values found for BLAST of *Yarrowia lipolytica *proteins against *Saccharomyces cerevisiae*, *Candida glabrata*, *Kluyveromyces lactis*, *Debaryomyces hansenii*, *Schizosaccharomyces pombe *(*Sp*),*Neurospora crassa*, other fungi, animals, plants, obtained with NCBI web site. Numbers between brackets indicate the order of best BLAST hits. Fungi: *Ashbya gossypii *(Ag), *Aspergillus clavatus *(Ac), *Aspergillus fumigatus *(Af), *Aspergillus nidulans *(Asn), *Aspergillus niger *(An), *Aspergillus orizae *(Ao), *Aspergillus parasiticus *(Ap), *Aspergillus terreus *(Ast), *Chaetomium globosum *(Chg), *Coccidioides immitis *(Ci), *Coprinopsis cinerea *(Cc), *Cryptococus neoformans *(Cn), *Gibberzlla zeae *(Gz), *Hypocrea lixii *(Hl), *Magnaporthe grisea *(Mg), *Neosartorya fischeri *(Nf), *Neurospora crassa *(Nc), *Paracoccidioides brasiliensis *(Pb), *Phaeosphaeria nodorum *(Pn), *Ustilago maydis *(Um). Animals: *Aedes aegypti *(Aa), *Aiptasia pulchella *(Ap), *Anopheles gambiae *(Ang), *Apis mellifera *(Am), *Bombyx mori *(Bm), *Bos taurus *(Bt), *Caenorhabditis briggsae *(Cb), *Caenorhabditis elegans *(Ce), *Canis familiaris *(Cf), *Danio rerio *(Dr), *Drosophila grimshawi *(Dg), *Drosophila melanogaster *(Dm), *Drosophila pseudoobscura *(Dp), *Gallus gallus *(Gg), *Homo sapiens *(Hs), *Macaca mulatta *(Mam), *Mus musculus *(Mm), *Oryzias latipes *(Ol), *Pan troglodytes *(Pt), *Pongo pygmaeus *(Pp), *Rattus norvegicus *(Rn), *Strongylocentrus purpuratus *(Stp), *Xenopus laevis *(Xl), *Xenopustropicalis *(Xt). Plants: *Arabidopsis thaliana *(At), *Brassica oleracea *(Bo), *Brassica rapa *(Br), *Hyacinthus orientalis *(Ho), *Lotus japonicus *(Lj), *Medicago truncatula *(Mt), *Nicotiana tabacum *(Nt), *Oenothera odorata *(Oo), *Oriza sativa *(Os), *Pisum sativum *(Ps), *Solanum chacoense *(Soc), *Solanum tuberosum *(St), *Zea mays *(Zm). (As *Debaryomyces hansenii *Vps35p, Snx3p, Gyp2p, Sec20p, Sec18p sequences were absent from the NCBI database when the comparison was done, the e-values were obtained with the NCBI BLAST of the *Debaryomyces hansenii *protein sequence against *Yarrowia lipolytica *sequences). Click here for file

Additional file 8 **E-values**. E-values found for NCBI BLAST of *Saccharomyces cerevisiae *proteins against *Yarrowia lipolytica *and animal proteins (see Additional file 7 legend for list of abbreviations). Click here for file

Additional file 2 **List of *Yarrowia lipolytica *genes coding for the proteins potentially implicated in vesicular transport**. They were obtained by comparison against *Saccharomyces cerevisiae *protein sequences, BLAST results come from Génolevures web site, if *Candida glabrata*, *Kluyveromyces lactis*, *Debaryomyces hansenii *protein was found is indicated (see Additional file 9 for the list of the *Candida glabrata*, *Kluyveromyces lactis*, *Debaryomyces hansenii *genes). Click here for file

Additional file 9 List of *Candida. glabrata, Kluyveromyces lactis, Debaryomyces hansenii *genes coding for the proteins potentially implicated in vesicular transport. Click here for file
