# Supplementary figures and images for: Yarrowia lipolytica vesicle-mediated protein transport pathways
Source: BMC Evol Biol. 2007 Nov 12;7:219. doi: 10.1186/1471-2148-7-219 (PMC2241642; doi:10.1186/1471-2148-7-219)

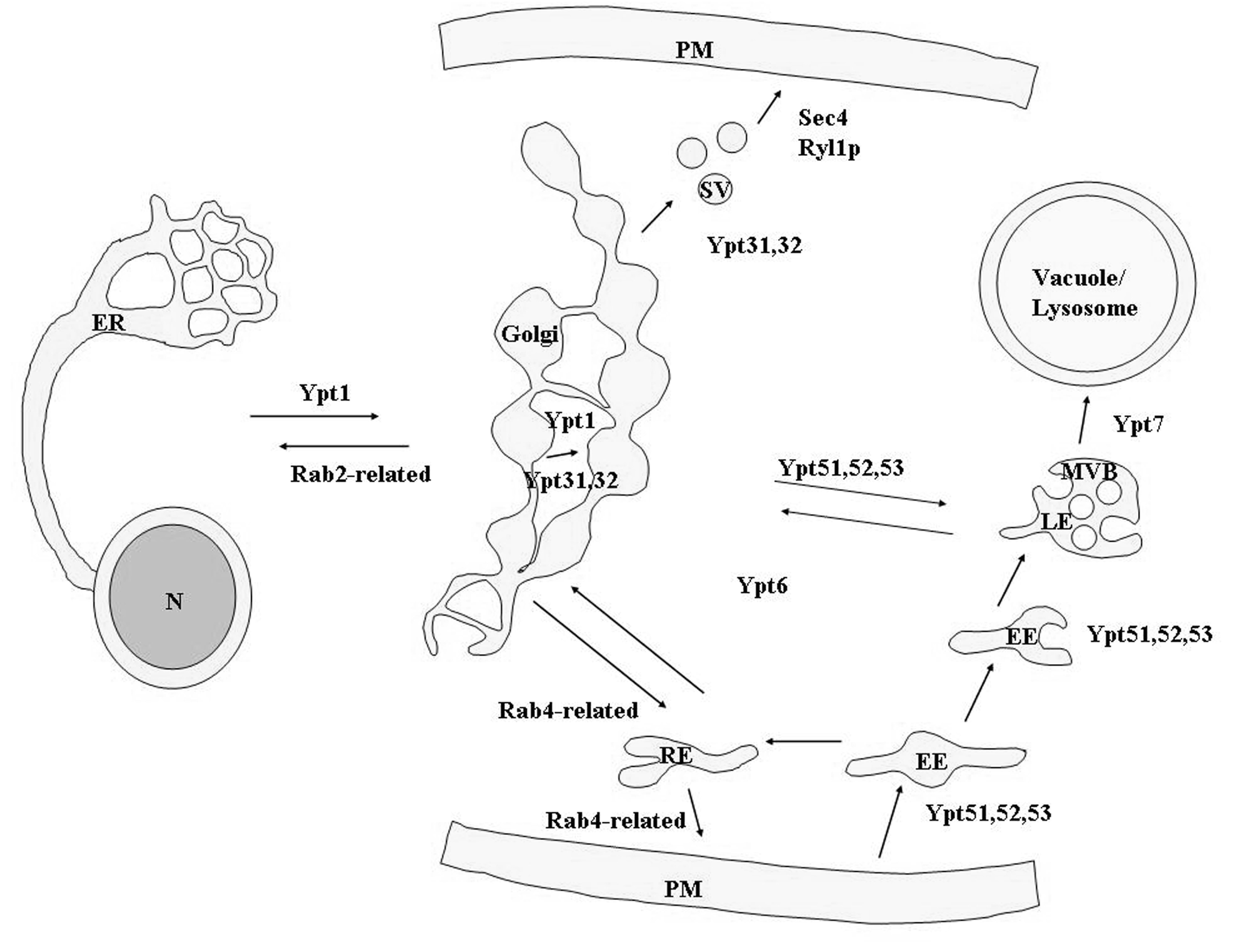

Supplement: Additional file 3 — Drawing of Yarrowia lipolytica identified Ypt/Rab GTPases. PM: plasma membrane, ER: endoplasmic reticulum, RE: recycling endosome, EE: early endosome, LE: late endosome, MVB: multi-vesicular bodies, SV: secretory vesicle. [file 1471-2148-7-219-S3.jpeg]

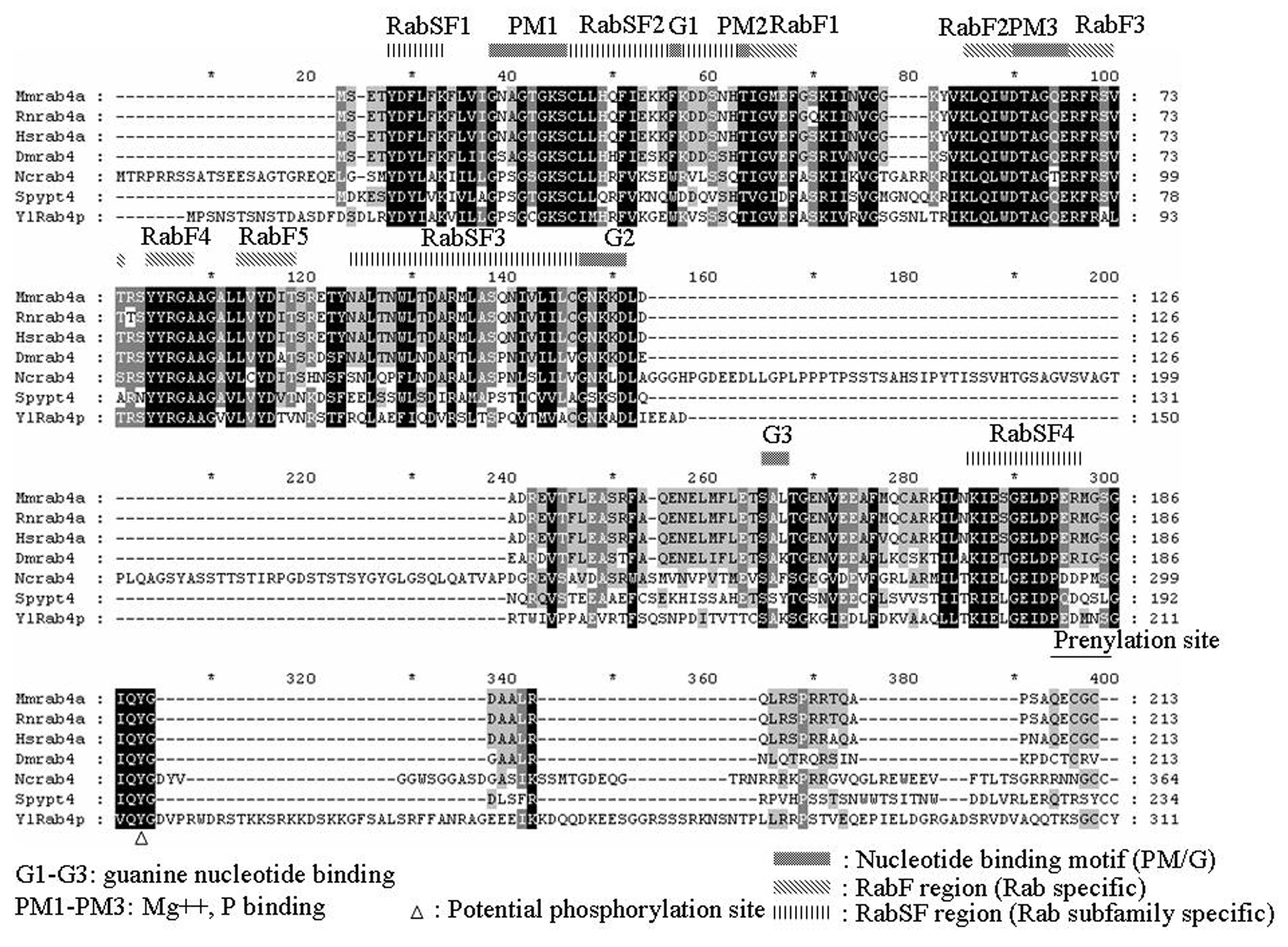

Supplement: Additional file 4 — Full image of Figure 2 [file 1471-2148-7-219-S4.jpeg]

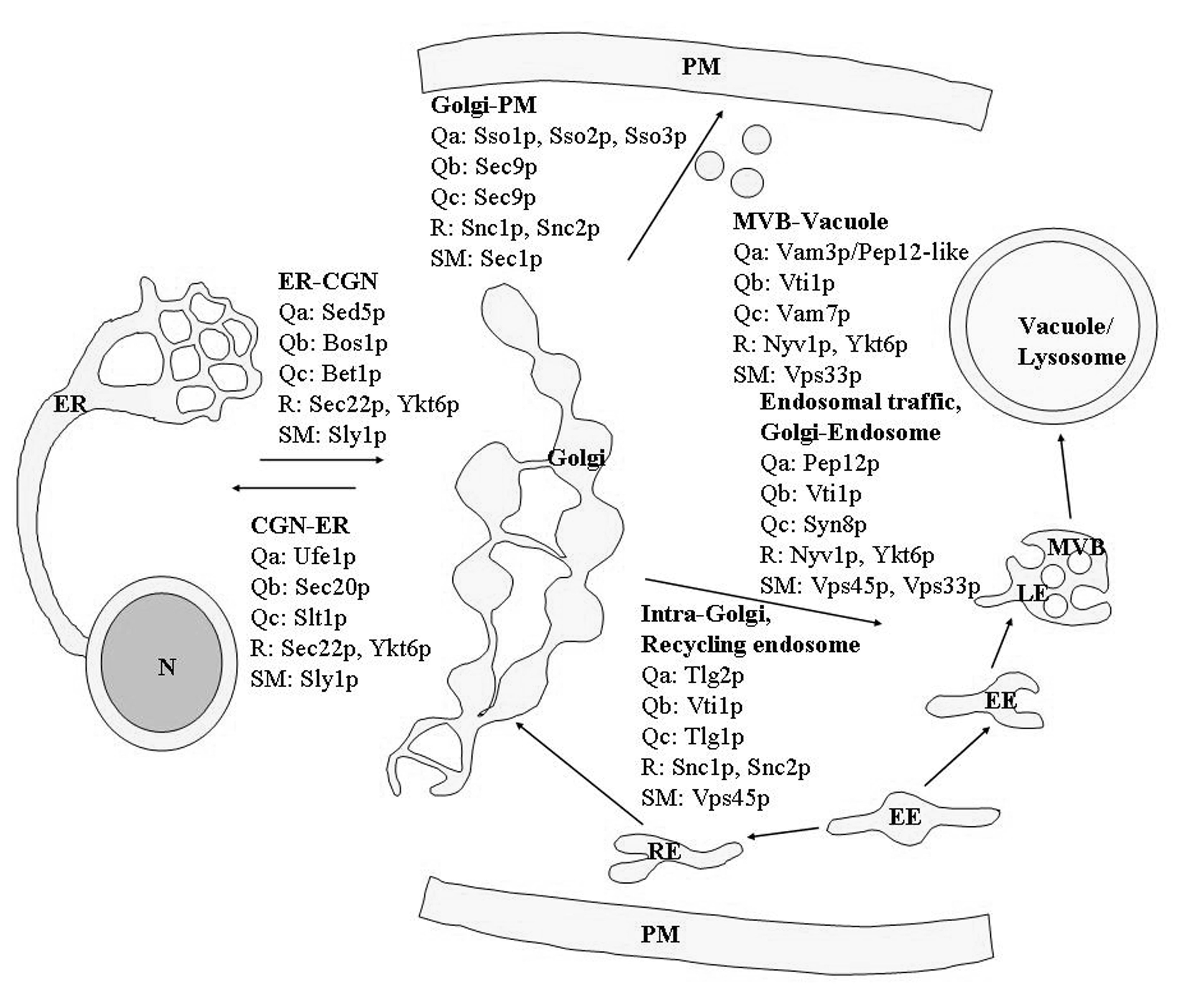

Supplement: Additional file 6 — Drawing of Yarrowia lipolytica identified SNARE and SM proteins. PM: plasma membrane, ER: endoplasmic reticulum, RE: recycling endosome, EE: early endosome, LE: late endosome, MVB: multi-vesicular bodies, SV: secretory vesicle. [file 1471-2148-7-219-S6.jpeg]
