# Supplementary material for: Yarrowia lipolytica vesicle-mediated protein transport pathways
Source: BMC Evol Biol. 2007 Nov 12;7:219. doi: 10.1186/1471-2148-7-219 (PMC2241642; doi:10.1186/1471-2148-7-219)
Supplement: Additional file 7 — E-values. E-values found for BLAST of Yarrowia lipolytica proteins against Saccharomyces cerevisiae, Candida glabrata, Kluyveromyces lactis, Debaryomyces hansenii, Schizosaccharomyces pombe (Sp),Neurospora crassa, other fungi, animals, plants, obtained with NCBI web site. Numbers between brackets indicate the order of best BLAST hits. Fungi: Ashbya gossypii (Ag), Aspergillus clavatus (Ac), Aspergillus fumigatus (Af), Aspergillus nidulans (Asn), Aspergillus niger (An), Aspergillus orizae (Ao), Aspergillus parasiticus (Ap), Aspergillus terreus (Ast), Chaetomium globosum (Chg), Coccidioides immitis (Ci), Coprinopsis cinerea (Cc), Cryptococus neoformans (Cn), Gibberzlla zeae (Gz), Hypocrea lixii (Hl), Magnaporthe grisea (Mg), Neosartorya fischeri (Nf), Neurospora crassa (Nc), Paracoccidioides brasiliensis (Pb), Phaeosphaeria nodorum (Pn), Ustilago maydis (Um). Animals: Aedes aegypti (Aa), Aiptasia pulchella (Ap), Anopheles gambiae (Ang), Apis mellifera (Am), Bombyx mori (Bm), Bos taurus (Bt), Caenorhabditis briggsae (Cb), Caenorhabditis elegans (Ce), Canis familiaris (Cf), Danio rerio (Dr), Drosophila grimshawi (Dg), Drosophila melanogaster (Dm), Drosophila pseudoobscura (Dp), Gallus gallus (Gg), Homo sapiens (Hs), Macaca mulatta (Mam), Mus musculus (Mm), Oryzias latipes (Ol), Pan troglodytes (Pt), Pongo pygmaeus (Pp), Rattus norvegicus (Rn), Strongylocentrus purpuratus (Stp), Xenopus laevis (Xl), Xenopustropicalis (Xt). Plants: Arabidopsis thaliana (At), Brassica oleracea (Bo), Brassica rapa (Br), Hyacinthus orientalis (Ho), Lotus japonicus (Lj), Medicago truncatula (Mt), Nicotiana tabacum (Nt), Oenothera odorata (Oo), Oriza sativa (Os), Pisum sativum (Ps), Solanum chacoense (Soc), Solanum tuberosum (St), Zea mays (Zm). (As Debaryomyces hansenii Vps35p, Snx3p, Gyp2p, Sec20p, Sec18p sequences were absent from the NCBI database when the comparison was done, the e-values were obtained with the NCBI BLAST of the Debaryomyces hansenii protein sequence against Yarrowia lipol [file 1471-2148-7-219-S7.doc]

Additional file 7: E-values found for blast of *Yarrowia lipolytica* proteins

| *Yl* Protein | *Sc* | *Cg* | *Kl* | *Dh* | *Sp* | *Nc* | *Fungi* | Animal | Plant |
| --- | --- | --- | --- | --- | --- | --- | --- | --- | --- |
| **1. CopII** |  |  |  |  |  |  |  |  |  |
| Sar1p/*YALI0C21824g* | 1 e-74 | 4 e-73 | 2 e-73 | 1 e-80 | 1 e-78 | 3 e-81 | 1 e-86 (Mg) | 3 e-68 (Dm) | 1 e-68 (Os) |
| Sec23p/*YALI0E16995g* | 0 [7] | 0 [8] | 0 [6] | 0 [4] | 0 [3] | 0 [2] | 0 (Cc) [1] | 0 (Dr) [5] | 0 (Os) [9] |
| Sec23p/*YALI0D23705g* | 1 e-156 | 2 e-165 | 7 e-163 | 2 e-169 | 0 [2] | 1 e-180 | 0 (Chg) [1] | 4 e-159 (Dm) | 5 e-149 (Os) |
| Sec24p/*YALI0F05324g* | 0 [7] | 0 [5] | 0 [6] | 0 [4] | 0 [3] | 0 [2] | 0 (Ci) [1] | 2 e-132 (Hs) | 3 e-126 (Pn) |
| Sfb3p/*YALI0E14036g* | 1 e-95 | 5 e-90 | 2 e-95 | 9 e-131 | 6 e-120 | 4 e-152 | 6 e-162 (Gz) | 5 e-93 (Dr) | 6 e-85 (Os) |
| Sec13p/*YALI0F30151g* | 6 e-118 | 9 e-117 | 2 e-116 | 9 e-112 | 3 e-103 | 2 e-98 | 6 e-119 (Ag) | 5 e-86 (Hs) | 6 e-78 (Os) |
| Sec31p/*YALI0E30635g* | 8 e-117 | 5 e-103 | 2 e-109 | 1 e-123 | 9 e-87 | 4 e-147 | 3 e-139 (Chg) | 8 -88 (Dr) | 2 e-73 (At) |
| Sec16p/*YALI0B12694g* | 3 e-28 | 8 e-22 | 5 e-27 | 6 e-30 | 7 e-19 | 4 e-38 | 5 e-46 (Pn) | 3 e-19 (Mam) | 2 e-11 (At) |
| Sec12p/*YALI0A08646g* | 1 e-17 | 3 e-17 | 3 e-15 | 2 e-11 | - | 3 e-04 | 5 e-16 (Ag) | 3.2 (Dm) | 3.2 (Os) |
| **2. CopI** |  |  |  |  |  |  |  |  |  |
| Arf1p/*YALI0F02167g* | 2 e-80 | 7 e-80 | 2 e-81 | 1 e-88 | 4 e-86 | 1 e-83 | 1 e-85 (Cc) | 8 e-82 (Bm) | 1 e-81 (Ho) |
| Sec33p/*YALI0E19767g* | 0 [6] | 0 [5] | 0 [4] | 0 [2] | 0 [7] | 0 [3] | 0 (Gz) [1] | 0 (Gg) [8] | 0 (At) [9] |
| Ret2p/*YALI0E32542g* | 6 e-38 | 3 e-40 | 3 e-40 | 4 e-51 | 7 e-50 | 4 e-53 | 7 e-98 (Gz) | 5 e-42 (Dm) | 2 e-35 (Os) |
| Ret3p/*YALI0F13255g* | 4 e-27 | 1 e-27 | 2 e-30 | 8 e-27 | 5 e-29 | 2 e-29 | 6 e-33 (Ac) | 1 e-21 (Stp) | 2 e-16 (Os) |
| Sec21p/*YALI0F03454g* | 2 e-180 | 2 e-180 | 1 e-180 | 5 e-173 | 1 e-172 | 0 [2] | 0 (Ci) [1] | 2 e-159 (Stp) | 6 e-162 (Os) |
| Sec26p/*YALI0F19074g* | 0 [5] | 0 [4] | 0 [6] | 0 [3] | 0 [7] | 0 [1] | 0 (Mg) [2] | 0 (Pp) [8] | 0 (At) [9] |
| Sec27p/*YALI0C21802g* | 0 [3] | 0 [7] | 0 [4] | 0 [9] | 0 [8] | 0 [2] | 0 (Af) [1] | 0 (Gg) [5] | 0 (At) [6] |
| Sec28p/*YALI0E15994g* | 7 e-7 | 3 e-7 | 3 e-9 | 3 e-8 | 1 e-10 | 1 e-28 | 8 e-32 (Chg) | 8 e-17 (Bt) | 3 e-10 (At) |
| **3. AP complex** |  |  |  |  |  |  |  |  |  |
| **AP-1** |  |  |  |  |  |  |  |  |  |
| Apl2p/*YALI0F21769g* | 5 e-128 | 3 e-132 | 3 e-121 | 1 e-168 | 5 e-157 | 0 [1] | 0 (Mg) [2] | 4 e-173 (Cf) | 4 e-170 (At) |
| Apl4p/*YALI0B21340g* | 1 e-104 | 2 e-91 | 2 e-104 | 2 e-161 | 9 e-151 | 0 [2] | 0 (Ci) [1] | 2 e-157 (Dr) | 6 e-148 (At) |
| Apm1p/*YALI0B11682g* | 2 e-152 | 1 e-153 | 2 e-151 | 3 e-153 | 2 e-159 | 0 [1] | 0 (Gz) [2] | 1 e-157 (Cf) | 2 e-136 (Os) |
| Apspa/*YALI0B04246g* | 1 e-37 | 1 e-36 | 6 e-36 | 2 e-41 | 5 e-46 | 7 e-62 | 2 e-61 (Chg) | 7 e-46 (Stp) | 5 e-46 (At) |
| Apspb/*YALI0E29733g* | 4 e-46 | 2 e-46 | 2 e-45 | 1 e-53 | 3 e-39 | 4 e-34 | 2 e-45 (Ag) | 2 e-39 (Hs) | 1 e-30 (At) |
| Apm2p/*YALI0F19976g* | 5 e-103 | 2 e-103 | 1 e-98 | 5 e-94 | 1 e-100 | 9 e-114 | 2 e-115 (Ci) | 3 e-105 (Cf) | 2 e-96 (Os) |
| **AP-2** |  |  |  |  |  |  |  |  |  |
| Apl1p/*YALI0E15598g* | 1 e-100 | 2 e-87 | 2 e-100 | 4 e-151 | 4 e-121 | 5 e-170 | 8 e-180 (Ast) | 1 e-139 (Ce) | 2 e-132 (At) |
| Apl3p/*YALI0C18623g* | 2 e-87 | 1 e-78 | 3 e-76 | 5 e-105 | 6 e-120 | 0 [2] | 0 (Pn) [1] | 1 e-155 (Hs) | 1 e-122 (At) |
| Apm4p/*YALI0C12474g* | 3 e-83 | 4 e-94 | 4 e-95 | 8 e-100 | 4 e-100 | 3 e-138 | 8 e-140 (Ao) | 3 e-111 (Stp) | 6 e-101 (Os) |
| **AP-3** |  |  |  |  |  |  |  |  |  |
| Apl6p/*YALI0B20680g* | 2 e-72 | 1 e-60 | 2 e-69 | 2 e-87 | 3 e-81 | 2 e-98 | 3 e-95 (Ci) | 1 e-75 (Bt) | 4 e-36 (At) |
| Apl5p/*YALI0F00198g* | 4 e-125 | 2 e-115 | 3 e-116 | 3 e-103 | 3 e-112 | 2 e-86 | 7 e-119 (Ag) | 7 e-101 (Mm) | 3 e-70 (At) |
| Apm3p/*YALI0D17776g* | 4 e-22 | 7 e-15 | 3 e-22 | 1 e-43 | 3 e-12 | 2 e-20 | 4 e-29 (Ag) | 6 e-22 (Stp) | 1 e-20 (Os) |
| **Clathrin** |  |  |  |  |  |  |  |  |  |
| Chc1p/*YALI0A17127g* | 0 [3] | 0 [5] | 0 [6] | 0 [1] | 0 [7] | 0 [4] | 0 (Nf) [2] | 0 (Aa) [8] | 0 (Os) [9] |
| Clc1p/*YALI0E33253g* | 2 e-22 | 1 e-21 | 3 e-24 | 5 e-27 | 1 e-20 | 1 e-21 | 3 e-25 (Pn) | 2 e-8 (Aa) | 3 e-4 (Oo) |
| **Other adaptors** |  |  |  |  |  |  |  |  |  |
| Ggap/*YALI0D27192g* | 7 e-84 | 2 e-82 | 8 e-85 | 2 e-82 | 4 e-72 | 6 e-99 | 4 e-98 (Ac) | 1 e-18 (Dp) | 1 e-12 (Os) |
| Inp53p/*YALI0D06413g* | 0 [4] | 0 [5] | 0 [2] | 0 [6] | 0 [3] | 5 e-166 | 0 (Pn) [1] | 8 e-108 (Dm) | 5 e-47 (At) |
| **4. Retromer complex** |  |  |  |  |  |  |  |  |  |
| Vps5p/*YALI0A16797g* | 6 e-52 | 6 e-60 | 5 e-59 | 2 e-72 | 2 e-52 | 4 e-85 | 3 e-88 (Ao) | 4 e-45 (Gg) | 2 e-28 (Os) |
| Vps17p/*YALI0D21362g* | 9 e-52 | 1 e-52 | 2 e-55 | 3 e-52 | 4 e-45 | 4 e-65 | 6 e-69 (Asn) | 1 e-5 (Am) | 2.7 (Mt) |
| Vps26p/*YALI0D23793g* | 6 e-70 | 3 e-81 | 7 e-85 | 2 e-111 | 5 e-99 | 2 e-124 | 3 e-125 (Ao) | 8 e-92 (Xl) | 9 e-77 (Os) |
| Vps29p/*YALI0E19987g* | 9 e-28 | 1 e-28 | 2 e-26 | 2 e-29 | 6 e-27 | 5 e-34 | 7 e-39 (Ci) | 9 e-29 (Aa) | 3 e-26 (Os) |
| Vps35p/*YALI0E17413g* | 5 e-95 | 1 e-93 | 3 e-93 | 4 e-137 | 5 e-76 | 0 [2] | 0 (Pn) [1] | 1 e-155 (Ang) | 6 e-77 (Os) |
| **5. Sorting nexin** |  |  |  |  |  |  |  |  |  |
| Snx4p/*YALI0E13904g* | 9 e-69 | 3 e-62 | 4 e-58 | 4 e-32 | 8 e-55 | 9 e-80 | 6 e-82 (Ast) | 9 e-22 (Mm) | 2 e-16 (Bo) |
| Snx42p/*YALI0D07678g* | 3 e-48 | 2 e-36 | 2 e-50 | 7 e-41 | 2 e-42 | 1 e-87 | 1 e-96 (Asn) | 4 e-10 (Dr) | 4 e-4 (Os) |
| Snx3p/*YALI0F05456g* | 1 e-34 | 8 e-37 | 1 e-38 | 6 e-23 | 6 e-47 | 1 e-60 | 5 e-51 (Ast) | 2 e-32 (Am) | 1 e-13 (Os) |
| **6. Ypt proteins** |  |  |  |  |  |  |  |  |  |
| Ypt1p/*YALI0D08162g* | 2 e-79 | 4 e-81 | 8 e-84 | 1 e-87 | 3 e-90 | 5 e-94 1er | 5 e-94 (Chg) | 9 e-89 (Gg) | 4 e-84 (Lj) |
| Ypt6p/*YALI0B22154g* | 2 e-61 | 1 e-58 | 8 e-60 | 3 e-56 | 2 e-67 | - | 8 e-66 (Ao) | 1 e-65 (Xt) | 4 e-66 (At) |
| Ypt7p/*YALI0F19602g* | 1 e-70 | 8 e-68 | 1 e-68 | 9 e-65 | 2 e-74 | 2 e-84 | 3 e-85 (Ap) | 3 e-71 (Dr) | 1 e-70 (Ps) |
| Ypt31p/*YALI0D14630g* | 3 e-65 | 7 e-68 | - | 7 e-69 | 4 e-67 | 3 e-70 | 2 e-73 (Mg) | 8 e-70 (Gg) | 2 e-65 (At) |
| Ypt32p/*YALI0C14168g* | 2 e-33 | 9 e-34 | - | 1 e-34 | 8 e-33 | 2 e-33 | 4 e-34 (Cc) | 3 e-34 (Mm) | 2 e-36 (At) |
| Ypt51p/*YALI0D07128g* | 1 e-70 | 6 e-70 | 7 e-73 | 3 e-72 | 7 e-49 | 2 e-72 | 2 e-72 (Gz) | 3 e-54 (Bm) | 3 e-48 (At) |
| Ypt52p/*YALI0F27181g* | 2 e-49 | 3 e-47 | 3 e-50 | 4 e-53 | 3 e-62 | 2 e-67 | 5 e-71 (Ci) | 2 e-57 (Ap) | 2 e-52 (Nt) |
| Ypt53p/*YALI0A04367g* | 1 e-30 | 7 e-31 | 8 e-29 | 2 e-31 | 3 e-30 | 4 e-41 | 8 e-50 (Ci) | 8 e-32 (Ap) | 9 e-31 (Os) |
| Sec4p/*YALI0E23067g* | 1 e-63 | 2 e-63 | 9 e-64 | 1 e-68 | 2 e-70 | 3 e-77 | 2 e-78 (Gz) | 6 e-63 (Bt) | 1 e-68 (Lj) |
| Rab2p/*YALI0F23529g* | - | - | - | - | 3 e-38 | 3 e-53 | 2 e-55 (Gz) | 1 e-45 (Stp) | 9 e-44 (Nt) |
| Rab4p/*YALI0B18788g* | - | - | - | - | 4 e-48 | 1 e-43 | 5 e-54 (Ci) | 4 e-49 (Stp) | 5 e-33 (At) |
| **7. Ypt regulation** |  |  |  |  |  |  |  |  |  |
| **Prenylation** |  |  |  |  |  |  |  |  |  |
| Bet2p/*YALI0E28248g* | 9 e-78 | 5 e-77 | 5 e-72 | 2 e-71 | 5 e-80 | 2 e-84 | 1 e-87 (Ao) | 2 e-81 (Dr) | 1 e-81 (Os) |
| Bet4p/*YALI0E13662g* | 7 e-47 | 2 e-30 | 6 e-33 | 2 e-52 | 6 e-59 | 1 e-38 | 4 e-49 (Cn) | 7 e-48 (Xl) | 6 e-35 (Os) |
| Mrs6p/*YALI0F06116g* | 2 e-133 | 1 e-134 | 2 e-126 | 2 e-120 | 7 e-46 | 2 e-46 | 4 e-125 (Ag) | 6 e-29 (Ang) | 2 e-24 (At) |
| **GDI** |  |  |  |  |  |  |  |  |  |
| Gdi1p/*YALI0E33649g* | 0 [2] | 0 [5] | 0 [4] | 0 [1] | 2 e-171 | 4 e-146 | 0 (Ag) [3] | 1 e-150 (Dr) | 3 e-135 (Os) |
| **GDF** |  |  |  |  |  |  |  |  |  |
| Yif1p/*YALI0E26323g* | 2 e-26 | 4 e-29 | 3 e-26 | 4 e-43 | 6 e-57 | 2 e-58 | 5 e-67 (Asn) | 7 e-34 (Stp) | 9 e-26 (At) |
| Yip1p/*YALI0D04829g* | 2 e-46 | 2 e-45 | 4 e-44 | 9e -57 | 2 e-53 | 4 e-52 | 1 e-56 (Pn) | 2 e-43 (Cf) | 5 e-21 (At) |
| Yip2p/*YALI0B19668g* | 3 e-32 | 7 e-33 | 4 e-34 | 4 e-37 | 2 e-23 | 2 e-28 | 2 e-31 (Af) | 2 e-20 (Ce) | 9 e-15 (Os) |
| Yip3p/*Yl*Yip3p | 4 e-31 | 1 e-31 | 6 e-34 | 6 e-32 | 7 e-24 | 1 e-14 | 4 e-37 (Ao) | 6 e-5 (Stp) | 0.86 (At) |
| Yip4p/*YALI0F15279g* | 2 e-15 | 2 e-10 | 1 e-14 | 2 e-36 | 8 e-39 | 3 e-31 | 8 e-36 (Ci) | 1 e-29 (Am) | 7 e-29 (Os) |
| Yip5p/*YALI0E24299g* | 3 e-8 | 4 e-5 | 2 e-5 | 8 e-17 | 4 e-12 | 1 e-25 | 5 e-29 (Asn) | 8 e-20 (Aa) | 5 e-15 |
| **GEF** |  |  |  |  |  |  |  |  |  |
| Bet5p/*YALI0B10318g* | 2 e-16 | 3 e-14 | 0.001 | 8 e-19 | 3 e-19 | 1 e-32 | 5 e-37 (Ao) | 1 e-22 (Xl) | 2 e-16 (At) |
| Trs20p/*YALI0E03520g* | 1 e-24 | 5 e-25 | 3 e-23 | 2 e-30 | 7 e-30 | 0.65 | 3 e-34 (Mg) | 1 e-28 (Xl) | 1 e-26 (Br) |
| Bet3p/*YALI0C08782g* | 1 e-61 | 2 e-62 | 9 e-63 | 5 e-62 | 3 e-58 | 2 e-44 | 3 e-72 (Ci) | 8 e-56 (Cf) | 1 e-45 (At) |
| Trs23p/*YALI0B22396g* | 1 e-16 | 3 e-11 | 4 e-21 | 9 e-27 | 1 e-33 | 2 e-37 | 8 e-39 (Ci) | 3 e-28 (Xt) | 9 e-32 (At) |
| Trs31p/*YALI0B05720g* | 7 e-18 | 2 e-25 | 2 e-29 | 8 e-45 | 3 e-43 | 3 e-35 | 4 e-41 (Cn) | 1 e-33 (Dr) | 1 e-35 (Soc) |
| Trs33p/*YALI0E22902g* | 6 e-15 | 7 e-14 | 1 e-16 | 1 e-24 | 6 e-15 | 1 e-29 | 8 e-28 (Ci) | 4 e-20 (Stp) | 9 e-22 (At) |
| Trs85p/*YALI0E12177g* | 2 e-20 | 7 e-17 | 1 e-13 | 2 e-31 | 2 e-35 | 1 e-60 | 6 e-64 (Pn) | 5 e-29 (Am) | 7 e-12 (Os) |
| Trs65p/*YALI0B12144g* | 2 e-4 | 3 e-7 | 6 e-6 | 1 e-31 | 1.3 | 2 e-7 | 7 e-26 (Mg) | 2.8 (Rn) | - |
| Trs120p/*YALI0D15004g* | 2 e-45 | 7 e-40 | 1 e-42 | 1 e-43 | 1 e-46 | 3 e-59 | 7 e-61 Gz) | 1 e-9 (Am) | 4 e-5 (Os) |
| Trs130p/*YALI0B22726g* | 1 e-24 | 3 e-16 | 2 e-19 | 4 e-68 | 7 e-14 | 2 e-34 | 5 e-57 (Cn) | 2 e-32 (Am) | 4 e-19 (Os) |
| Sec2p/*YALI0F27379g* | 3 e-10 | 2 e-8 | 3 e-9 | 2 e-6 | 2 e-8 | 5 e-23 | 2 e-20 (Mg) | 9 e-5 (Dr) | 0.02 (Os) |
| Rgp1p/*YALI0F29513g* | 5 e-6 | 0.11 | 8 e-5 | - | 9 e-6 | 1 e-22 | 3 e-27 (Ao) | 2 e-6 (Am) | 4.5 (Os) |
| Ric1p/*YALI0A01397g* | 0.19 | 0.001 | 0.002 | 4 e-6 | 5 e-39 | 2 e-93 | 4 e-127 (Gz) | 2 e-48 (Dm) | 1 e-24 (At) |
| Vps9p/*YALI0E32593g* | 1 e-59 | 9 e-54 | 9 e-59 | 4 e-70 | 7 e-75 | 2 e-67 | 5 e-89 (Gz) | 5 e-54 (Mm) | 6 e-34 (At) |
| Vps39p/*YALI0B11550g* | 6 e-31 | 2 e-17 | 2 e-23 | 3 e-47 | 2 e-63 | 5 e-73 | 7 e-100 (Ast) | 2 e-55 (Am) | 6 e-27 (At) |
| **GAP, GYP-like protein** |  |  |  |  |  |  |  |  |  |
| Gyp1p/*YALI0B12100g* | 2 e-77 | 1 e-99 | 1 e-100 | 2 e-103 | 5 e-115 | 2 e-135 | 5 e-135 (Af) | 1 e-97 (Dr) | 3 e-87 (Os) |
| Gyp5p/*YALI0F18106g* | 6 e-93 | 9 e-94 | 3 e-99 | 1 e-95 | 5 e-47 | 2 e-84 | 5 e-98 (Ag) | 8 e-58 (Gg) | 5 e-38 (At) |
| Gyp8p/*YALI0C24332g* | 5 e-22 | 7 e-24 | 8 e-26 | 3 e-26 | 5 e-20 | 2 e-34 | 1 e-39 (Ac) | 1 e-24 (Mm) | 1.2 (At) |
| Gyp3p/*YALI0B22792g* | 1 e-96 | 3 e-95 | 2 e-100 | 4 e-94 | 1 e-89 | 3 e-90 | 1 e-100 (Ag) | 2 e-43 (Stp) | - |
| Gyp2p/*YALI0B01628g* | 0 [4] | 0 [3] | 0 [1] | 0 | 2 e-135 | 1 e-167 | 0 (Af) [2] | 3 e-73 (Bt) | 1 e-38 (Os) |
| Gyp6p/*YALI0C22968g* | 4 e-9 | 2 e-11 | 1 e-9 | 4 e-11 | 3 e-14 | 2 e-41 | 2 e-43 (Gz) | 4 e-47 (Stp) | 8 e-24 (Os) |
| Gyp7p/*YALI0F31911g* | 2 e-120 | 2 e-134 | 4 e-131 | 3 e-173 | 2 e-124 | 4 e-170 | 2 e-175 (Ac) | 5 e-65 (Pt) | 2 e-59 (Os) |
| **8. COG complex** |  |  |  |  |  |  |  |  |  |
| Cog2p/*YALI0A20592g* | - | 0.07 | - | 2 e-5 [1] | 2 e-5 | 3 e-6 | 2 e-15 (Ao) | 3 e-6 (Dr) | 0.008 (Os) |
| Cog3p/*YALI0F17666g* | 5 e-71 | 8 e-53 | 2 e-69 | 1 e-79 | 2 e-40 | 1 e-17 | 1 e-68 (Ci) | 3 e-46 (Dp) | 8 e-43 (At) |
| Cog4p/*YALI0D03113g* | 8 e-47 | 1 e-41 | 5 e-41 | 2 e-59 | 2 e-46 | - | 2 e-78 (Asn) | 3 e-40 (Stp) | 1 e-22 (At) |
| Cog5p/*YALI0F05280g* | 9 e-19 | 5 e-17 | 2 e-22 | 2 e-17 | - | 9 e-19 | 1 e-19 (Pn) | 1 e-6 (Am) | 0.001 (Os) |
| Cog6p/*YALI0F16467g* | 2 e-20 | 2 e-16 | 2 e-18 | 3 e-43 | 1 e-22 | 4 e-56 | 1 e-58 (Pn) | 1 e-41 (Am) | 9 e-23 (Os) |
| Cog8p/*YALI0F16203g* | 5 e-14 | 1 e-08 | 7 e-16 | 5 e-15 | 1 e-14 | 6 e-18 | 7 e-24 (Cn) | 7 e-14 (Hs) | 1 e-16 (At) |
| **9.Uso1, Imh1, Rud3, Coy1 and Grh1 proteins** |  |  |  |  |  |  |  |  |  |
| Uso1p/*YALI0F02387g* | 6 e-170 | 2 e-171 | 7 e-160 | 0 | 7 e-81 | 5 e-122 | 3 e-148 (Ag) | 2 e-61 (Xl) | 1 e-41 (At) |
| Imh1p/*YALI0F30855g* | 1 e-20 | 9 e-17 | 2 e-21 | 3 e-43 | - | 1 e-24 | 8 e-40 (Pn) | 6 e-17 (Am) | 2 e-7 (Os) |
| Rud3p/*YALI0B18634g* | 4 e-44 | 9 e-46 | 1 e-44 | 6 e-42 | 9 e-44 | 8 e-48 | 3 e-48 (Gz) | 1 e-18 (Dr) | 2 e-14 (At) |
| Coy1p/*YALI0C18821g* | 4 e-81 | 4 e-79 | 2 e-72 | 1 e-98 | 6 e-51 | 3 e-92 | 2 e-93 (Pn) | 1 e-57 (Dr) | 6 e-54 (Os) |
| Grh1p/*YALI0E03014g* | 4 e-6 | 3 e-6 | 1 e-4 | 2 e-12 1er | 2 e-12 | 5 e-15 | 5 e-21 (Pn) | 2 e-18 (Dp) | - |
| **10. Dsl1p complex** |  |  |  |  |  |  |  |  |  |
| Dsl1p/*YALI0A07689g* | 0.005 | 0.052 | 0.15 | 0.002 | - | 2 e-26 | 1 e-46 (Ac) | 6 e-8 (Dg) | 1 e-4 (Os) |
| Tip20p/*YALI0C15334g* | 0.003 | 2 e-5 | 0.53 | 2 e-30 | 2 e-10 | 4 e-43 | 1 e-65 (Nf) | 1 e-28 (Stp) | 0.11 (Os) |
| Dsl3p/*YALI0B12914g* | 3 e-15 | 1 e-6 | 5 e-16 | 1 e-10 | 6 e-7 | 6 e-17 | 5 e-25 (Ast) | 6.9 (Ol) | 0.002 (At) |
| **11.Arf, Arf-like proteins and Arl3p localization** |  |  |  |  |  |  |  |  |  |
| Arf1p/*YALI0F02167g* | 1 e-80 | 7 e-80 | 2 e-81 | 1 e-88 | 4 e-86 | 1 e-83 | 1 e-85 (Cc) | 1 e-81 (Bt) | 1 e-81 (Ho) |
| Arf3p/*YALI0C14586g* | 1 e-61 | 6 e-60 | 7 e-61 | 4 e-60 | 5 e-68 | 9 e-67 | 1 e-75 (Ci) | 7 e-72 (Gg) | 8 e-63 (Zm) |
| Sar1p/*YALI0C21824g* | 1 e-74 | 4 e-73 | 2 e-73 | 1 e-80 | 1 e-78 | 3 e-81 | 1 e-86 (Mg) | 4 e-69 (Ang) | 1 e-69 (Br) |
| Arl1p/*YALI0F31009g* | 9 e-78 | 9 e-78 [1] | 4 e-75 | 3 e-75 | 2 e-52 | 3 e-65 | 2 e-76 (Ag) | 6 e-63 (Rn) | 3 e-62 (At) |
| Arl2p/*YALI0C14586g* | 1 e-61 | 6 e-60 | 7 e-61 | 4 e-60 | 5 e-68 | 9 e-67 | 1 e-75 (Ci) | 2 e-72 (Aa) | 8 e-63 (Zm) |
| Arl3p/*YALI0D02995g* | 8 e-62 | 1 e-58 | 7 e-28 | 1 e-39 | 6 e-28 | 1 e-48 | 3 e-57 (Ag) | 7 e-45 (Am) | 1 e-30 (At) |
| Cin4p/*Yl*Cin4p | 2 e-32 | 2 e-32 | 6 e-33 | 1 e-47 | 3 e-41 | 1 e-50 | 1 e-53 (Cn) | 5 e-51 (Bt) | 1 e-50 (At) |
| Arl1p-like/*YALI0E11803g* | 2 e-22 | 1 e-21 | 3 e-24 | 4 e-22 | - | 4 e-65 | 3 e-67 (Pn) | 8 e-55 (Bm) | 2 e-52 (Os) |
| Sys1p/*YALI0B18656g* | 2 e-26 [1] | 3 e-23 | 2 e-23 | 6 e-25 | - | 1 e-19 | 2 e-26 (Af) | 3 e-11 (Dm) | 6 e-7 (Os) |
| Mak3p/*YALI0C11539g* | 3 e-41 | 4 e-43 | 3 e-44 | 3 e-31 | 3 e-32 | 2 e-32 | 3 e-40 (Ag) | 3 e-32 (Mm) | 6 e-30 |
| Mak10p/*YALI0F08591g* | 9 e-22 | 2 e-26 | 3 e-29 | 3 e-36 | 2 e-14 | 1 e-34 | 3 e-42 (Gz) | 1 e-30 (Dr) | 7 e-11 (Os) |
| **12. GARP complex** |  |  |  |  |  |  |  |  |  |
| Vps52p/*YALI0F07381g* | 3 e-39 | 2 e-36 | 4 e-41 | 2 e-36 | 2 e-29 | 8 e-46 | 8 e-54 (Pn) | 2 e-20 (Mm) | 1 e-19 (At) |
| Vps53p/*YALI0D11198g* | 4 e-63 | 1 e-65 | 4 e-60 | 2 e-103 | 3 e-90 | 7 e-129 | 1 e-134 (Pn) | 3 e-79 (Gg) | 8 e-64 |
| Vps54p/*YALI0B16822g* | 9 e-46 | 8 e-37 | 3 e-48 | 7 e-67 | 2 e-42 | 6 e-72 | 1 e-84 (Af) | 1 e-15 (Ang) | 3 e-14 (At) |
| 13. HOPS complex |  |  |  |  |  |  |  |  |  |
| Vps11p/*YALI0E23408g* | 8 e-115 | 7 e-103 | 4 e-127 | 7 e-157 | 1 e-61 | 7 e-138 | 2 e-155 (Af) | 1 e-85 (Dr) | 3 e-39 (At) |
| Vps18p/*YALI0A19008g* | 3 e-64 | 1 e-77 | 8 e-90 | 6 e-45 | 2 e-87 | 7 e-142 | 2 e-166 (Mg) | 6 e-106 (Gg) | 2 e-85 (At) |
| Vps16p/*YALI0A03553g* | 7 e-53 | 2 e-32 | 1 e-50 | 1 e-59 | 2 e-98 | 2 e-128 | 7 e-144 (Af) | 1 e-83 (Gg) | 7 e-91 (At) |
| Vps41p/*YALI0F17710g* | 2 e-115 | 4 e-85 | 2 e-122 | 8 e-132 | 3 e-73 | 8 e-44 | 1 e-126 (Ag) | 6 e-73 (Pt) | 4 e-78 (Os) |
| Vps39p/*YALI0B11550g* | 6 e-31 | 2 e-17 | 2 e-23 | 3 e-47 | 2 e-63 | 5 e-63 | 7 e-100 (Ast) | 2 e-55 (Am) | 6 e-27 (At) |
| Vps33p/*YALI0F04125g* | 5 e-30 | 9 e-37 | 2 e-33 | 1 e-23 | 3 e-54 | 2 e-66 | 2 e-78 (Ci) | 4 e-57 (Rn) | 2 e-48 (At) |
| **14. Exocyst complex** |  |  |  |  |  |  |  |  |  |
| Sec3p/*YALI0F21681g* | 2 e-39 | 1 e-36 | 8 e-43 | 7 e-36 | - | 3 e-72 | 6 e-77 (Pn) | 2 e-23 (Stp) | 1 e-9 (Os) |
| Sec8p/*YALI0E33759g* | 4 e-85 | 4 e-71 | 7 e-72 | 3 e-86 | 2 e-26 | 3 e-111 | 2 e-112 (Mg) | 9 e-40 (Gg) | 9 e-17 (Os) |
| Sec5p/*YALI0A19052g* | 9 e-44 | 8 e-50 | 9 e-62 | 4 e-33 | 0.022 | 1 e-75 | 2 e-75 (Ac) | 1 e-10 (Pp) | 9 e-20 (Os) |
| Sec15p/*YALI0F12969g* | 4 e-63 | 3 e-63 | 2 e-65 | 2 e-76 | 3 e-68 | 2 e-92 | 4 e-107 (Nf) | 4 e-65 (Mm) | 4 e-48 (At) |
| Sec10p/*YALI0C01595g* | 2 e-48 | 6 e-89 | 2 e-84 | 5 e-106 | 2 e-44 | 2 e-75 | 2 e-102 (Ag) | 7 e-24 (Xl) | 5 e-25 (Os) |
| Sec6p/*YALI0D08492g* | 2 e-101 | 2 e-101 [1] | 1 e-99 | 3 e-60 | 2 e-77 | 1 e-86 | 1 e-110 (Cn) | 6 e-26 (Ce) | 6 e-25 (At) |
| Exo84p/*YALI0F11143g* | 5 e-40 | 2 e-49 | 2 e-40 | 1.8 | 1 e-9 | 5 e-45 | 2 e-75 (Ast) | 6 e-28 (Gg) | 0.007 (Os) |
| Exo70p/*YALI0C11946g* | 5 e-56 | 4 e-55 | 2 e-63 | 2 e-53 | 1 e-18 | 2 e-47 | 1 e-65 (Ag) | 3 e-19 (Xt) | 4 e-10 (Os) |
| **15.Exocyst regulation proteins** |  |  |  |  |  |  |  |  |  |
| Rho1p/*YALI0E23001g* | 1 e-86 | 1 e-83 | 6 e-84 | 4 e-86 | 4 e-90 | 3 e-61 | 5 e-89 (Pb) | 3 e-75 (Aa) | - |
| Rho3p/*YALI0F17270g* | 2 e-74 | 4 e-76 | 2 e-80 | 3 e-48 | 2 e-70 | 9 e-73 | 2 e-77 (Ag) | 8 e-47 (Hs) | - |
| Cdc42p/*YALI0B15752g* | 4 e-95 | 5 e-95 | 2 e-95 [1] | 6 e-92 | 7 e-93 | 5 e-83 | 2 e-95 (Um) | 4 e-86 (Dr) | - |
| **16. SNARE proteins** |  |  |  |  |  |  |  |  |  |
| **Qa** |  |  |  |  |  |  |  |  |  |
| Ufe1p/*YALI0C10846g* | 3 e-15 | 2 e-13 | 4 e-18 | 2 e-20 | 1 e-18 | 6 e-27 | 5 e-25 (Gz) | 2 e-7 (Dr) | 6 e-9 (Os) |
| Sed5p/*YALI0C16819g* | 2 e-46 | 2 e-42 | 8 e-42 | 1 e-55 | 3 e-53 | 9 e-56 | 3 e-58 (Mg) | 2 e-45 (Dr) | 3 e-34 |
| Tlg2p/*YALI0E33165g* | 3 e-40 | 4 e-43 | 3 e-41 | 7 e-40 | 5 e-36 | 2 e-38 | 3 e-47 (Mg) | 3 e-27 (Mm) | 3 e-25 (Os) |
| Pep12p/*YALI0E11825g* | 2 e-19 | 1 e-16 | 2 e-15 | 6 e-16 | 6 e-13 | 2 e-24 | 8 e-25 (Chg) | 5 e-15 (Bt) | 2 e-10 (At) |
| Pep12p-like/*YALI0C22275g* | 3 e-15 | 4 e-12 | 1 e-13 | 2 e-7 | 3 e-9 | 2 e-19 | 8 e-24 (Chg) | 2 e-8 (Rn) | 2 e-4 (Os) |
| Ssop/*YALI0D25872g* | 1 e-55 | 2 e-54 | 1 e-55 [1] | 2 e-49 | 1 e-39 | 3 e-36 | 3 e-55 (Ag) | 7 e-19 (Dm) | 3 e-17 (At) |
| Ssop/*YALI0B10780g* | 2 e-57 | 6 e-58 | 2 e-57 | 9 e-60 | 1 e-48 | 3 e-38 | 3 e-57 (Ag) | 1 e-20 (Xt) | 8 e-19 (At) |
| Ssop/*YALI0E23243g* | 3 e-56 | 3 e-57 | 2 e-58 | 1 e-55 | 2 e-45 | 3 e-38 | 1 e-58 (Ag) | 1 e-16 (Dr) | 4 e-17 (At) |
| **Qb** |  |  |  |  |  |  |  |  |  |
| Sec20p/*YALI0C05302g* | 4 e-13 | 3 e-15 | 4 e-11 | 3 e-22 | 7 e-7 | 1 e-7 | 3 e-17 (Ci) | 1 e-4 (Xt) | 4 e-9 (Os) |
| Bos1p/*YALI0F31669g* | 6 e-24 | 1 e-23 | 9 e-22 | 5 e-31 | 6 e-26 | 5 e-26 | 1 e-32 (Ci) | 5 e-11 (Stp) | 1 e-6 (At) |
| Gos1p/*YALI0D23353g* | 7 e-32 | 2 e-30 | 5 e-35 | 3 e-40 | 5 e-9 | 5 e-32 | 2 e-39 (Pn) | 8 e-22 (Cf) | 6 e-17 (At) |
| Vti1p/*YALI0B02244g* | 2 e-31 | 2 e-25 | 6 e-23 | 4 e-39 | 3 e-27 | 2 e-35 | 6 e-36 (Af) | 3 e-20 (Hs) | 2 e-16 (St) |
| Sec9p/*YALI0E18414g* | 2 e-37 | 1 e-39 | 5 e-35 | 1 e-31 | 2 e-24 | 0.59 | 1 e-37 (Pn) | 0.34 (Cf) | 2.9 (Os) |
| **Qc** |  |  |  |  |  |  |  |  |  |
| Slt1p/*YALI0C21626g* | 0.003 | 5 e-5 | 5.8 | 2 e-7 | - | - | 2 e-8 (Ag) | 0.081 (Stp) | 0.021 (Os) |
| Sft1p/*YALI0E05269g* | 1 e-11 | - | 4 e-11 | 0.14 | 9 e-6 | 5 e-14 | 1 e-12 (Mg) | 7.5 (Stp) | 0.009 (At) |
| Bet1p/*YALI0F25223g* | 1 e-13 | 1 e-13 (1er) | 2 e-17 | 5 e-13 | 3 e-9 | 2 e-21 [1] | 2 e-21 (Asn) | 8 e-6 (Ce) | 0.13 (Os) |
| Tlg1p/*YALI0B05786g* | 4 e-18 | 5 e-24 | 3 e-23 | 1 e-22 | 2 e-24 | 5 e-25 | 1 e-26 (Pn) | 1 e-9 (Stp) | 4 e-8 (At) |
| Syn8p/*YALI0E17875g* | 2 e-8 [1] | 2 e-8 | 5 e-11 [1] | 8 e-15 | 9 e-8 | 5 e-11 | 3 e-19 (Nf) | 1 e-7 (Cf) | 2 e-6 (Os) |
| Vam7p/*YALI0C16412g* | 5 e-8 | 6 e-12 | 7 e-11 | 5 e-11 | 7 e-10 | 4 e-10 | 2 e-22 (Ci) | 7 e-7 (Rn) | 3 e-7 (At) |
| **R** |  |  |  |  |  |  |  |  |  |
| Sec22p/*YALI0D21956g* | 1 e-54 | 2 e-54 | 5 e-58 | 1 e-58 | 8 e-64 | 2 e-58 | 3 e-62 (Ao) | 1 e-42 (Xl) | 1 e-44 (St) |
| Ykt6p/*YALI0E21329g* | 9 e-72 | 8 e-69 | 2 e-69 | 7 e-72 | 4 e-63 | 1 e-49 | 2 e-70 (Ag) | 2 e-49 (Mm) | 4 e-46 (At) |
| Nyv1p/*YALI0B04026g* | 2 e-10 [1] | 2 e-12 | 2 e-10 | 1 e-9 | 4 e-7 | 3 e-24 | 2 e-32 (Cn) | 1 e-19 (Dm) | 8 e-23 (Os) |
| Snc1p/*YALI0A03113g* | 1 e-28 | 5 e-30 | 4 e-28 | 1 e-29 | 1 e-28 | 1 e-22 | 1 e-30 (Ci) | 2 e-12 (Dr) | 9 e-12 (Os) |
| Snc2p/*YALI0E00594g* | 8 e-34 | 4 e-30 | 5 e-33 | 2 e-33 | 3 e-31 | 6 e-28 | 1 e-34 (Ci) | 1 e-14 (Dr) | 2 e-12 (Os) |
| **17. SNARE binding proteins** |  |  |  |  |  |  |  |  |  |
| Sly1p/*YALI0D20416g* | 5 e-104 | 7 e-105 | 2 e-105 | 3 e-102 | 5 e-119 | 1 e-110 | 2 e-120 (Ac) | 2 e-93 (Dr) | 4 e-93 (At) |
| Vps33p/*YALI0F04125g* | 5 e-30 | 9 e-37 | 2 e-33 | 1 e-23 | 3 e-54 | 2 e-66 | 2 e-78 (Ci) | 4 e-57 (Rn) | 2 e-48 (At) |
| Vps45p/*YALI0E29337g* | 1 e-113 | 3 e-110 | 3 e-104 | 6 e-83 | 4 e-126 | 4 e-147 | 2 e-155 (Ci) | 8 e-122 (Stp) | 7 e-105 (At) |
| Sec1p/*YALI0E22044g* | 1 e-89 | 4 e-85 | 4 e-90 | 3 e-97 | 4 e-93 | 6 e-88 | 2 e-106 (Ao) | 4 e-63 (Ce) | 8 e-48 (Os) |
| **18.Exocytosis SNARE regulation proteins** |  |  |  |  |  |  |  |  |  |
| Vsm1p/*YALI0B06754g* | 5 e-52 | 6 e-54 | 8 e-51 | 6 e-48 | 7 e-42 | 7 e-57 | 4 e-61 (Gz) | 2 e-58 (Dm) | 5 e-56 (At) |
| Tpd3p/ *YALI0F00836g* | 4 e-154 | 8 e-142 | 4 e-154 [1] | 1 e-144 | 2 e-155 | 1 e-165 | 6 e-177 (Pn) | 3 e-132 (Cf) | 3 e-145 (At) |
| Cdc55p/*YALI0F03223g* | 5 e-164 | 5 e-167 | 2 e-172 | 1 e-178 | 9 e-172 | 0 2e | 0 (Pn) [1] | 3 e-136 (Xt) | 2 e-128 (At) |
| Sit4p/*YALI0F14069g* | 6 e-146 | 9 e-145 | 3 e-144 | 4 e-147 | 3 e-151 | 9 e-142 | 6 e-154 (Hl) | 2 e-149 (Dr) | 2 e-146 (Os) |
| Tpkp/*YALI0C08305g* | 3 e-158 | 8 e-157 | 4 e-159 | 6 e-157 | 8 e-112 | 4 e-139 | 3 e-156 (Ag) | 1 e-92 (Xl) | - |
| **19.SNARE recycling proteins** |  |  |  |  |  |  |  |  |  |
| Sec17p/*YALI0C23947g* | 1 e-56 | 5 e-59 | 5 e-70 | 4 e-61 | 7 e-72 | 8 e-60 | 9 e-66 (Asn) | 2 e-49 (Dr) | 1 e-46 (Os) |
| Sec18p/*YALI0E29249g* | 0 [4] | 0 [3] | 0 [1] | 0 | 0 [5] | 3 e-179 | 0 (Ag) [2] | 6 e-166 (Mm) | 3 e-146 (Nt) |
| Rcy1p/Sls2p/*YALI0B19074g* | 2 e-81 | 2 e-72 | 2 e-74 | 8 e-91 | 3 e-88 | 1 e-103 | 3 e-145 (Gz) | 0.001 (Cf) | 0.003 (Os) |
