# Supplementary material for: Yarrowia lipolytica vesicle-mediated protein transport pathways
Source: BMC Evol Biol. 2007 Nov 12;7:219. doi: 10.1186/1471-2148-7-219 (PMC2241642; doi:10.1186/1471-2148-7-219)
Supplement: Additional file 8 — E-values. E-values found for NCBI BLAST of Saccharomyces cerevisiae proteins against Yarrowia lipolytica and animal proteins (see Additional file 7 legend for list of abbreviations). [file 1471-2148-7-219-S8.doc]

Additional file 8: E-values found for NCBI blast of *Saccharomyces cerevisiae* proteins with *Yarrowia lipolytica* and animal proteins.

| *Sc* Proteins | *Yl* | Animal |
| --- | --- | --- |
| **1. CopII** |  |  |
| Sar1p | 1 e-74 | 9 e-61 (Dm) |
| Sec23p | 0 | 0 (Am) [2] |
| Sec24p | 0 | 5 e-109 (Rn) |
| Sfb3p | 6 e-97 | 1 e-71 (Dr) |
| Sec13p | 2 e-111 | 6 e-82 (Stp) |
| Sec31p | 9 e-146 | 8 e-73 |
| Sec16p | 1 e-46 | 3 e-9 (Hs) |
| Sec12p | 2 e-25 | 0.008 (Dm) |
| **2. CopI** |  |  |
| Arf1p | 2 e-80 | 3 e-80 (Ce) |
| Sec33p | 0 | 0 (Pt) [2] |
| Ret2p | 2 e-38 | 2 e-52 (Stp) |
| Ret3p | 4 e-37 | 7 e-28 (Xl) |
| Sec21p | 0 | 5 e-133 (Mm) |
| Sec26p | 0 | 0 (Pp) [2] |
| Sec27p | 0 | 0 (Pt) [2] |
| Sec28p | 0.005 | 0.057 (Hs) |
| **3. AP complex** |  |  |
| **AP-1** |  |  |
| Apl2p | 4 e-140 | 5 e-124 (Hs) |
| Apl4p | 9 e-99 | 2 e-79 (Xl) |
| Apm1p | 4 e-151 | 2 e-130 (Cf) |
| Aps1p | 3 e-21 | 2 e-42 (Xt) |
| Aps2p | 1 e-31 | 1 e-31 (Dr) [2] |
| Aps3p | 8 e-44 | 3 e-30 (Hs) |
| Apm2p | 4 e-29 | 4 e-28 (Cf) |
| **AP-2** |  |  |
| Apl1p | 5 e-95 | 4 e-74 (Dr) |
| Apl3p | 7 e-88 | 1 e-69 (Bt) |
| Apm4p | 5 e-71 | 8 e-70 (Am) |
| **AP-3** |  |  |
| Apl6p | 3 e-70 | 2 e-63 (Hs) |
| Apl5p | 6 e-133 | 4 e-98 (Am) |
| Apm3p | 2 e-19 | 3 e-11 (Dr) |
| **Clathrin** |  |  |
| Chc1p | 0 | 0 (Cf) [2] |
| Clc1p | 3 e-23 | 0.01 (Stp) |
| **Other adaptors** |  |  |
| Gga2p | 5 e-97 | 1 e-20 (Dr) |
| Inp53p | 0 | 7 e-110 (Cf) |
| **4. Retromer complex** |  |  |
| Vps5p | 3 e-61 | 1 e-33 (Bt) |
| Vps17p | 1 e-55 | 0.001 (Hs) |
| Vps26p | 8 e-69 | 2 e-53 (Am) |
| Vps29p | 4 e-43 | 3 e-27 (Dm) |
| Vps35p | 2 e-141 | 1 e-109 (Dr) |
| **5. Sorting nexin** |  |  |
| Snx4p | 4 e-71 | 8 e-25 (Xt) |
| Snx42p | 1 e-54 | 2 e-6 (Pp) |
| Snx3p | 2 e-34 | 6 e-20 (Hs) |
| **6. Ypt proteins** |  |  |
| Ypt1p | 2 e-79 | 8 e-80 (Hs) |
| Ypt6p | 2 e-61 | 8 e-67 (Aa) |
| Ypt7p | 1 e-70 | 2 e-66 (Dr) |
| Ypt31p | 9 e-62 | 2 e-68 (Dr) |
| Ypt32p | 4 e-64 | 2 e-68 (Xt) |
| Ypt51p | 3 e-61 | 9 e-47 (Rn) |
| Ypt52p | 3 e-46 | 7 e-44 (Aa) |
| Ypt53p | 9 e-48 | 1 e-43 (Gg) |
| Sec4p | 4 e-61 | 4 e-59 (Ce) |
| **7. Ypt regulation** |  |  |
| **Prenylation** |  |  |
| Bet2p | 1 e-77 | 1 e-94 (Hs) |
| Bet4p | 7 e-47 | 3 e-35 (Xl) |
| Mrs6p | 8 e-128 | 2 e-30 (Stp) |
| **GDI** |  |  |
| Gdi1p | 0 | 1 e-142 (Dr) |
| **GDF** |  |  |
| Yif1p | 4 e-24 | 5 e-20 (Ang) |
| Yip1p | 1 e-47 | 4 e-34 (Ce) |
| Yip2p | 5 e-38 | 4 e-24 (Ce) |
| Yip3p | - | 6 e-5 (Xl) |
| Yip4p | 4 e-11 | 1 e-6 (Stp) |
| Yip5p | 1 e-9 | 1 e-8 (Mm) |
| **GEF** |  |  |
| Bet5p | 1 e-11 | 3 e-16 (Xl) |
| Trs20p | 2 e-24 | 4 e-23 (Aa) |
| Bet3p | 1 e-61 | 8 e-52 (Gg) |
| Trs23p | 5 e-9 | 3 e-13 (Xl) |
| Trs31p | 2 e-25 | 2 e-11 (Gg) |
| Trs33p | 3 e-16 | 1 e-14 (Cf) |
| Trs85p | 4 e-17 | 0.53 (Stp) |
| Trs65p | 0.082 | 1.2 (Rn) |
| Trs120p | 9 e-40 | 3 e-4 (Am) |
| Trs130p | 2 e-18 | 2 e-7 (Hs) |
| Sec2p | 7 e-12 | 2 e-4 (Xl) |
| Rgp1p | 4 e-5 | 5.5 (Dr) |
| Ric1p | 0.012 | 4.2 (Dm) |
| Vps9p | 3 e-61 | 2 e-30 (Stp) |
| Vps39p | 8 e-33 | 2 e-31 (Bt) |
| **GAP, GYP-like protein** |  |  |
| Gyp1p | 2 e-77 | 7 e-69 (Bt) |
| Gyp5p | 1 e-88 | 4 e-46 (Bt) |
| Gyp8p | 5 e-22 | 7 e-13 (Stp) |
| Gyp3p | 7 e-96 | 7 e-42 (Dr) |
| Gyp2p | 5 e-178 | 3 e-66 (Rn) |
| Gyp6p | 5 e-9 | 1 e-9 (Dr) |
| Gyp7p | 7 e-119 | 2 e-53 (Am) |
| **9. COG complex** |  |  |
| Cog2p | - | 2 e-4 (Cf) |
| Cog3p | 1 e-73 | 4 e-46 (Dr) |
| Cog4p | 1 e-44 | 8 e-18 (Aa) |
| Cog5p | 2 e-12 | 0.041 (Dm) |
| Cog6p | 1 e-16 | 2 e-14 (Xl) |
| Cog8p | 3 e-17 | 3 e-6 |
| **10.Uso1, Imh1, Rud3, Coy1 and Grh1 proteins** |  |  |
| Uso1p | 0 | 5 e59 (Am) |
| Imh1p | 1 e-44 | 1 e-24 (Am) |
| Rud3p | 1 e-32 | 4 e-12 (Am) |
| Coy1p | 3 e-77 | 4 e-52 (Dr) |
| Grh1p | 7 e-20 | 3 e-11 (Aa) |
| **11. Dsl1p complex** |  |  |
| Dsl1p | 0.09 | 1.3 (Dr) |
| Tip20p | 0.003 | 1e-8 (Stp) |
| Dsl3p/Sec39p | 3 e-15 | 0.017 (Am) |
| **12.Arf, Arf-like proteins and Arl3p localization** |  |  |
| Arf1p | 2 e-80 | 9 e-81 (Ap) |
| Arf3p | 2 e-61 | 7 e-57 (Hs) |
| Sar1p | 1 e-74 | 9 e-61 (Dm) |
| Arl1p | 5 e-66 | 1 e-55 (Dr) |
| Arl2p | 2 e-61 | 7 e-57 (Hs) |
| Arl3p | 1 e-56 | 2 e-47 (Dp) |
| Cin4p | 9 e-18 | 1 e-25 (Gg) |
| Sys1p | 3 e-20 | 2 e-4 (Dm) |
| Mak3p | 1 e-46 | 1 e-35 (Xl) |
| Mak10p | 2 e-22 | 1 e-13 |
| **13. GARP complex** |  |  |
| Vps51p | - | 3 e-4 (Mm) |
| Vps52p | 2 e-45 | 5 e-20 (Ang) |
| Vps53p | 6 e-60 | 3 e-36 (Aa) |
| Vps54p | 8 e-42 | 3 e-6 |
| **14. HOPS complex** |  |  |
| Vps11p | 2 e-114 | 3 e-51 (Dr) |
| Vps18p | 2 e-60 | 6 e-51 (Xl) |
| Vps16p | 5 e-57 | 5 e-43 (Am) |
| Vps41p | 4 e-120 | 1 e-56 (Cf) |
| Vps39p | 8 e-33 | 2 e-31 (Bt) |
| Vps33p | 5 e-30 | 6 e-34 (Gg) |
| **15. Exocyst complex** |  |  |
| Sec3p | 2 e-39 | 8 e-7 |
| Sec8p | 4 e-91 | 1 e-20 (Dr) |
| Sec5p | 4 e-38 | 0.002 (Mm) |
| Sec15p | 7 e-63 | 1 e-26 (Mm) |
| Sec10p | 6 e-82 | 8 e-15 (Gg) |
| Sec6p | 2 e-94 | 7 e-18 (Dr) |
| Exo84p | 4 e-41 | 3 e-9 (Gg) |
| Exo70p | 2 e-56 | 1 e-8 (Rn) |
| **16.Exocyst regulation proteins** |  |  |
| Rho1p | 3 e-82 | 2 e-73 (Aa) |
| Rho3p | 1 e-76 | 2 e-48 (Dr) |
| Cdc42p | 3 e-94 | 4 e-87 (Mm) |
| **17. SNARE proteins** |  |  |
| **Qa** |  |  |
| Ufe1p | 3 e-13 | 9 e-5 (Aa) |
| Sed5p | 1 e-50 | 1 e-36 (Stp) |
| Tlg2p | 1 e-47 | 2 e-23 (Gg) |
| Pep12p | 5 e-18 | 3 e-13 (Cf) |
| Vam3p | 2 e-7 | 1 e-8 (Mm) |
| Sso1p | 2 e-60 | 2 e-22 (Cb) |
| Sso2p | 4 e-61 | 1 e-24 (Cb) |
| **Qb** |  |  |
| Sec20p | 2 e-11 | 0.14 (Xl) |
| Bos1p | 3 e-27 | 0.014 (Stp) |
| Gos1p | 2 e-35 | 2 e-16 (Gg) |
| Vti1p | 5 e-33 | 5 e-15 (Mm) |
| Sec9p | 7 e-48 | 6 e-12 (Rn) |
| Spo20p | 3 e-21 | 3 e-6 (Mam) |
| **Qc** |  |  |
| Slt1p | 0.003 | 0.2 (Mm) |
| Sft1p | 3 e-16 | 4 e-4 (Gg) |
| Bet1p | 2 e-17 | 3 e-6 (Mam) |
| Tlg1p | 3 e-16 | 8 e-4 (Dr) |
| Syn8p | 3 e-8 | 0.007 (Hs) |
| Vam7p | 6 e-11 | 8 e-5 (Xl) |
| **R** |  |  |
| Sec22p | 3 e-44 | 9 e-31 (Xl) |
| Ykt6p | 1 e-71 | 1 e-49 (Mm) |
| Nyv1p | 7 e-10 | 3 e-9 (Dr) |
| Snc1p | 7 e-30 | 7 e-11 (Dr) |
| Snc2p | 2 e-28 | 2 e-11 (Dr) |
| **18. SNARE binding proteins** |  |  |
| Sly1p | 3 e-109 | 1 e-98 (Dr) |
| Vps33p | 5 e-30 | 6 e-34 (Gg) |
| Vps45p | 6 e-118 | 3 e-99 (Dr) |
| Sec1p | 1 e-89 | 2 e-52 (Mm) |
| **19.Exocytosis SNARE regulation proteins** |  |  |
| Vsm1p | 5 e-46 | 1 e-41 (Xl) |
| Tpd3p | 7 e-154 | 1 e-131 (Cf) |
| Cdc55p | - | 8 e-116 (Ang) |
| Sit4p | 1 e-103 | 9 e-115 (Aa) |
| Tpk1p | 5 e-43 | 3 e-94 (Rn) |
| Tpk2p | 3 e-156 | 7 e-91 (Xl) |
| Tpk3p | 3 e-156 | 7 e-91 (Xl) |
| **20.SNARE recycling proteins** |  |  |
| Sec17p | 9 e-58 | 2 e-41 (Am) |
| Sec18p | 0 | 6 e-174 (Mm) |
| Rcy1p | 1 e-73 | 0.002 (Aa) |
