# Supplementary material for: Yarrowia lipolytica vesicle-mediated protein transport pathways
Source: BMC Evol Biol. 2007 Nov 12;7:219. doi: 10.1186/1471-2148-7-219 (PMC2241642; doi:10.1186/1471-2148-7-219)
Supplement: Additional file 2 — List of Yarrowia lipolytica genes coding for the proteins potentially implicated in vesicular transport. They were obtained by comparison against Saccharomyces cerevisiae protein sequences, BLAST results come from Génolevures web site, if Candida glabrata, Kluyveromyces lactis, Debaryomyces hansenii protein was found is indicated (see Additional file 9 for the list of the Candida glabrata, Kluyveromyces lactis, Debaryomyces hansenii genes). [file 1471-2148-7-219-S2.doc]

Additional file 2: List of *Yarrowia lipolytica* proteins potentially implicated in vesicular transport.

| *S. cerevisiae* | Description | *Y. lipolytica* | Positives | Gaps | e-value | *Cg* | *Kl* | *Dh* |
| --- | --- | --- | --- | --- | --- | --- | --- | --- |
|  |  |  |  |  |  |  |  |  |
| **1.COPII vesicle coat proteins** |  |  |  |  |  |  |  |  |
| Sar1p (190 aa) | Small GTP binding protein | *YALI0C21824g* (190 aa, 1 intron) | 82% (156/188) | 1% | 4 e-76 | 1 | 1 | 1 |
| Sec23p (768 aa) | GTPase activator | *YALI0E16995g* (758 aa, 1 intron)  *YALI0D23705g* (750 aa) | 69% (538/774)  57% (447/771) | 2%  4% | 0.0  e-157 | 2 | 1 | 2 |
| Yhr035wp (630 aa) | Sec23p-related | *YALI0E16995g* (758 aa, 1 intron)  *YALI0D23705g* (750 aa) | 41% (200/481)  37% (252/667) | 18%  16% | 6 e-17  1 e-10 | 0 | 1 | 0 |
| Sec24p (926 aa) | Cargo binding subunit | *YALI0F05324g* (934 aa) | 60% (579/961) | 7% | 0.0 | 2 | 1 | 1 |
| Sfb2p (876 aa) | Sec24p-related protein | *YALI0F05324g* (934 aa) | 65% (532/812) | 4% | 0.0 | 0 | 0 | 0 |
| Sfb3p (929 aa) | Sec24p-related protein | *YALI0E14036g* (906 aa) | 50% (410/813) | 6% | e-101 | 1 | 1 | 1 |
| Sec13p (297 aa) | COPII coat component | *YALI0F30151g* (298 aa, 1 intron) | 78% (236/300) | 1% | e-119 | 2 | 1 | 1 |
| Sec31p (1273 aa) | COPII coat component | *YALI0E30635g* (1184 aa) | 47% (630/1318) | 14% | e-157 | 1 | 1 | 1 |
| Sec16p (2195 aa) | Peripheral associated protein, interaction with Sar1p | *YALI0B12694g* (2183 aa, 1 intron) | 42% (214/501) | 14% | 6 e-30 | 1 | 1 | 1 |
| Sec12p (471 aa) | Sar1p-GEF | *YALI0A08646g* (362 aa, 1 intron) | 40% (133/329) | 3% | 3 e-20 | 1 | 1 | 1 |
| Sed4p (1065 aa) | Sec12p homolog putative Sar1p GAP inhibitor | No hits |  |  |  | 1 | 0 | 0 |
|  |  |  |  |  |  |  |  |  |
| **2.COPI vesicle coat proteins** |  |  |  |  |  |  |  |  |
| Arf1p (181aa) | GTP binding protein | *YALI0F02167g* (181 aa) | 90% (162/179) | 0% | 1 e-82 | 1 | 0 | 1 |
| Sec33p (1201 aa) | Ret1, COP1 | *YALI0E19767g* (1244 aa) | 72% (622/863) | 4% | 0.0 | 1 | 1 | 1 |
| Ret2p (546 aa) |  | *YALI0E32542g* (519 aa, 1 intron) | 64% (120/187) | 1% | 1 e-40 | 1 | 1 | 1 |
| Ret3p (189 aa) |  | *YALI0F13255g* (189 aa) | 72% (114/157) | 1% | 2 e-37 | 1 | 1 | 1 |
| Sec21p (935 aa) |  | *YALI0F03454g* (923 aa) | 59% (565/945) | 3% | 0.0 | 1 | 1 | 1 |
| Sec26p (973 aa) |  | *YALI0F19074g* (952 aa, 1 intron) | 65% (641/972) | 3% | 0.0 | 1 | 1 | 1 |
| Sec27p (889 aa) |  | *YALI0C21802g* (879 aa, 1 intron) | 70% (569/809) | 3% | 0.0 | 1 | 1 | 1 |
| Sec28p (296 aa) |  | *YALI0E15994g* (286 aa, 1 intron) | 41% (110/263) | 10% | 2 e-8 | 1 | 1 | 1 |
|  |  |  |  |  |  |  |  |  |
| **3.AP complex** |  |  |  |  |  |  |  |  |
| **AP-1** |  |  |  |  |  |  |  |  |
| Apl2p (726 aa) | β1 | *YALI0F21769g* (782 aa) | 61% (452/739) | 8% | e-145 | 1 | 1 | 1 |
| Apl4p (832 aa) | γ | *YALI0B21340g* (806 aa) | 55% (464/843) | 8% | e-119 | 1 | 1 | 1 |
| Apm1p (475 aa) | μ1 | *YALI0B11682g* (450 aa) | 75% (358/477)  46% (231/496)  43% (160/366)  43% 112/256) | 6%  10%  29%  19% | *S.c.* Apm1p e-154  *S.c.* Apm4p 4 e-45  *S.c.* Apm2p 1 e-31  *S.c.* Apm3p 6 e-9 | 1 | 1 | 1 |
| Aps1p (156 aa) | σ1 | *YALI0B04246g* (144 aa, 1 intron)  *YALI0E29733g* (189 aa) | 68% (97/141)  55% (77/140) | 2%  8% | 4 e-31  3 e-14 | 1 | 1 | 1 |
| Apm2p (605 aa) |  | *YALI0F19976g* (514 aa) | 60% (311/514)  47% (245/519)  43% (168/390)  44% (109/247) | 13%  14%  27%  12% | *S.c.* Apm1p e-106  *S.c.* Apm4p 2 e-43  *S.c.* Apm2p 3 e-30  *S.c.* Apm3p 9 e-8 | 1 | 1 | 1 |
| **AP-2** |  |  |  |  |  |  |  |  |
| Apl1p (700 aa) | β2R | *YALI0E15598g* (717 aa, 1 intron) | 57% (364/634) | 5% | e-102 | 1 | 1 | 1 |
| Apl3p (1025 aa) | αR | *YALI0C18623g* (929 aa) | 47% (476/996) | 10% | 1 e-98 | 1 | 1 | 1 |
| Apm4p (491 aa) | μ2R | *YALI0C12474g* (460 aa, 1 intron) | 57% (287/495)  50% (249/493)  37% (142/374)  38% (165/425) | 7%  10%  32%  17% | *S.c.* Apm4p 8e-85  *S.c.* Apm1p 1 e-68  *S.c.* Apm2p 2 e-16  *S.c.* Apm3p 6 e-5 | 1 | 1 | 1 |
| Aps2p (147 aa) | σ2R | *YALI0B04246g* (144 aa, 1 intron)  *YALI0E29733g* (189 aa) | 76% (111/145)  49% (68/138) | 2%  7% | 5 e-39  8 e-12 | 1 | 1 | 1 |
| **AP-3** |  |  |  |  |  |  |  |  |
| Apl6p (809 aa) | β3 | *YALI0B20680g* (783 aa) | 49% (400/806) | 10% | 3 e-79 | 1 | 1 | 1 |
| Apl5p (932 aa) | δ | *YALI0F00198g* (829 aa, 1 intron) | 52% (446/842) | 11% | e-127 | 1 | 1 | 1 |
| Apm3p (483 aa) | μ3 | *YALI0D17776g* (396 aa) | 43% (212/484)  50% (96/191)  47% (57/119) | 19%  6%  7% | *S.c.* Apm3p 2e-24  *S.c.* Apm1p 2 e-11  *S.c.* Apm4p 5 e-6  *S.c.* Apm2p, no hits | 1 | 1 | 1 |
| Aps3p (194 aa) | σ3 | *YALI0E29733g* (189 aa)  *YALI0B04246g* (144 aa, 1 intron) | 69% (125/179)  58% (94/160) | 5%  10% | 3 e-48  1 e-18 | 1 | 1 | 1 |
|  |  |  |  |  |  |  |  |  |
| **Clathrin** |  |  |  |  |  |  |  |  |
| Chc1p | Heavy chain | *YALI0A17127g* (1571 aa) | 77%(1248/1609) | 1% | 0.0 | 1 | 1 | 1 |
| Clc1p | Light chain | *YALI0E33253g* (217 aa) | 53% (127/236) | 10% | 1 e-27 | 1 | 1 | 1 |
|  |  |  |  |  |  |  |  |  |
| **Other adaptors** |  |  |  |  |  |  |  |  |
| Gga1p (557 aa) | Golgi | *YALI0D27192g* (616 aa) | 54% (326/593) | 11% | 3 e-91 | 0 | 0 | 0 |
| Gga2p (585 aa) | Golgi | *YALI0D27192g* (616 aa) | 57% (345/595) | 10% | e-107 | 1 | 1 | 1 |
| Inp53p (1107 aa) | Golgi | *YALI0D06413g* (1061 aa) | 60% (648/1066) | 9% | 0.0 | 1 | 1 | 1 |
|  |  |  |  |  |  |  |  |  |
| **4.Retromer complex proteins** |  |  |  |  |  |  |  |  |
| Vps5p (312 aa) | Sorting nexin | *YALI0A16797g* (632 aa) | 47% (295/627) | 11% | 1 e-61 | 1 | 1 | 1 |
| Vps17p (551 aa) | Sorting nexin | *YALI0D21362g* (581 aa) | 48% (297/608) | 14% | 5 e-59 | 1 | 1 | 1 |
| Vps26p (379 aa) |  | *YALI0D23793g* (300 aa) | 57% (217/379) | 21% | 2 e-72 | 1 | 1 | 1 |
| Vps29p (282 aa) |  | *YALI0E19987g* (286 aa, 1 intron) | 61% (106/171) | 7% | 3 e-29 | 1 | 1 | 1 |
| Vps35p (944 aa) |  | *YALI0E17413g* (848 aa) | 54% (490/892) | 18% | e-152 | 1 | 1 | 1 |
|  |  |  |  |  |  |  |  |  |
| **5.Sorting nexin proteins** |  |  |  |  |  |  |  |  |
| Snx4p (423 aa) |  | *YALI0E13904g* (436 aa, 1 intron) | 57% (233/407) | 4% | 2 e-70 | 1 | 1 | 1 |
| Snx41p (625 aa) |  | *YALI0D07678g* (570 aa) | 45% (250/549) | 12% | 2 e-47 | 1 | 1 | 1 |
| Snx42p (640 aa) |  | *YALI0D07678g* (570 aa) | 49% (265/532) | 8% | 4 e-55 | 1 | 1 | 1 |
| Snx3p (162 aa) | Grd19p | *YALI0F05456g* (152 aa, 1 intron) | 75% (102/135) | 7% | 5 e-36 | 1 | 1 | 1 |
|  |  |  |  |  |  |  |  |  |
| **6.Ypt proteins** |  |  |  |  |  |  |  |  |
| Ypt1p (206 aa) | ER-Golgi, Intra-Golgi | *YALI0D08162g* (202 aa, 1 intron) | 83% (172/207) | 2% | 5 e-81 | 1 | 1 | 1 |
| Ypt6p (215 aa) | Endosome-Golgi | *YALI0B22154g* (200 aa) | 73% (159/215) | 6% | 1 e-63 | 1 | 1 | 1 |
| Ypt7p (208 aa) | Golgi-Vacuole | *YALI0F19602g* (205 aa, 1 intron) | 77% (162/208) | 1% | 3 e-72 | 1 | 1 | 2 |
| Ypt10p (199 aa) | Endocytosis | No hits |  |  |  | 1 | 0 | 0 |
| Ypt11p (355 aa) | ER inheritance | No hits |  |  |  | 1 | 1 | 0 |
| Ypt31p (223 aa) | Trans-Golgi, Exocystosis | *YALI0D14630g* (216 aa)  *YALI0C14168g* (191 aa, 1 intron) | 76% (159/209)  63% (101/158) | 3%  0% | 4 e-66  6 e-35 | 1 | 1 | 0 |
| Ypt32p (222 aa) | Trans-Golgi, Exocystosis | *YALI0D14630g* (216 aa)  *YALI0C14168g* (191 aa, 1 intron) | 77% (165/214)  63% (103/161) | 3%  0% | 2 e-67  6 e-35 | 1 | 0 | 1 |
| Ypt51p (210 aa)  Ypt52p (234 aa)  Ypt53p (220 aa) | Endocytosis | *YALI0D07128g* (209 aa) | 79% (163/206)  60% (139/230)  70% (139/197) | 3%  13%  2% | 7 e-73  1 e-44  1 e-53 | 1  1  0 | 1  1  1 | 1  1  1 |
| Ypt51p (210 aa)  Ypt52p (234 aa)  Ypt53p (220 aa) | Endocytosis | *YALI0F27181g* (207 aa, 1 intron) | 69% (142/203)  68% (130/191)  59% (123/208) | 5%  14%  8% | 5 e-51  7 e-48  4 e-43 |  |  |  |
| Ypt51p (210 aa)  Ypt52p (234 aa)  Ypt53p (220 aa) | Endocytosis | *YALI0A04367g*/Ryl2p (209 aa) | 61% (113/184)  52% (122/232)  54% (111/204) | 2%  13%  6% | 1 e-32  1 e-31  3 e-28 |  |  |  |
| Sec4p (215 aa) | Exocytosis | *YALI0E23067g*/Ryl1p (203 aa) | 78% (162/207) | 3% | 8 e-66 | 1 | 1 | 1 |
|  | Mammalian Rab2ap-like | *YALI0F23529g* (213 aa, 1 intron) |  |  |  | 0 | 0 | 0 |
|  | Mammalian Rab4bp-like | *YALI0B18788g* (311 aa) |  |  |  | 0 | 0 | 0 |
|  |  |  |  |  |  |  |  |  |
| **7.Ypt regulation** |  |  |  |  |  |  |  |  |
| **Prenylation** |  |  |  |  |  |  |  |  |
| Bet2p (325 aa) | GGTase II | *YALI0E28248g* (277 aa) | 67% (183/272) | 2% | 4 e-78 | 1 | 1 | 1 |
| Bet4p (327 aa) | GGTase II | *YALI0E13662g* (347 aa, 1 intron) | 55% (187/340) | 7% | 2 e-48 | 1 | 1 | 1 |
| Mrs6p (603 aa) | REP | *YALI0F06116g* (566 aa) | 65% (377/578) | 5% | e-143 | 1 | 1 | 1 |
| **GDI** |  |  |  |  |  |  |  |  |
| Gdi1p (451 aa) | Sec19p | *YALI0E33649g* (450 aa) | 86% (384/445) | 0% | 0.0 | 1 | 1 | 1 |
| **GDF** |  |  |  |  |  |  |  |  |
| Yos1p (85 aa) | Yip1p-Yif1p complex | No hits |  |  |  | 0 | 0 | 1 |
| Yif1p (314 aa) | Yip1p-binding | *YALI0E26323g* (340 aa) | 49% (162/325) | 14% | 5 e-29 | 1 | 1 | 1 |
| Yip1p (248 aa) | Golgi, Ypt1p,31p-binding | *YALI0D04829g* (238 aa) | 65% (157/239) | 7% | 4 e-54 | 1 | 1 | 1 |
| Yip2p (180 aa) | Vesicular traffic in stressed cells | *YALI0B19668g* (189 aa, 1 intron) | 55% (99/179) | 0% | 7 e-34 | 1 | 1 | 1 |
| Yip3p (176 aa) | Yptp-binding | *Yl*Yip3p (164 aa, 1 intron)  (S. Casaregola, personal communication) | 79% (38/48) | 0% | 1 e-8 | 1 | 1 | 1 |
| Yip4p (235 aa) | Vesicle transport | *YALI0F15279g* (227 aa) | 49% (108/220) | 14% | 9 e-13 | 1 | 1 | 1 |
| Yip5p (310 aa) | Vesicle transport | *YALI0E24299g* (302 aa) | 40% (131/325) | 16% | 1 e-10 | 1 | 1 | 1 |
| **GEF** |  |  |  |  |  |  |  |  |
| Bet5p (159 aa) | TRAPP I, II ; Ypt1p,31p, 32p-GEF | *YALI0B10318g* (189 aa, 1 intron) | 46% (84/179) | 25% | 4 e-18 | 1 | 1 | 1 |
| Trs20p (175 aa) | TRAPP I, II ; Ypt1p,31p, 32p-GEF | *YALI0E03520g* (141 aa) | 54% (96/177) | 22% | 1 e-26 | 1 | 1 | 1 |
| Bet3p (193 aa) | TRAPP I, II ; Ypt1p,31p, 32p-GEF | *YALI0C08782g* (190 aa, 1 intron) | 77% (146/189) | 1% | 4 e-63 | 1 | 1 | 1 |
| Trs23p (219 aa) | TRAPP I, II ; Ypt1p,31p, 32p-GEF | *YALI0B22396g* (135 aa) | 44% (96/218) | 38% | 3 e-17 | 1 | 1 | 1 |
| Trs31p (283 aa) | TRAPP I, II ; Ypt1p,31p, 32p-GEF | *YALI0B05720g* (281 aa) | 47% (124/259) | 23% | 6 e-26 | 1 | 1 | 1 |
| Trs33p (268 aa) | TRAPP I, II ; Ypt1p,31p, 32p-GEF | *YALI0E22902g* (178 aa) | 60% (75/123) | 6% | 4 e-17 | 1 | 1 | 1 |
| Trs85p (698 aa) | TRAPP I, II ; Ypt1p,31p, 32p-GEF | *YALI0E12177g* (573 aa) | 41% (223/537) | 20% | 7 e-21 | 1 | 1 | 1 |
| Trs65p (560 aa) | TRAPP II ; Ypt1p,31p, 32p-GEF | *YALI0B12144g* (608 aa) | 40% (77/91) | 14% | 0.002 | 1 | 1 | 1 |
| Trs120p (1289 aa) | TRAPP II ; Ypt1p,31p, 32p-GEF | *YALI0D15004g* (1097 aa) | 39% (494/1243) | 18% | 3 e-45 | 1 | 1 | 1 |
| Trs130p (1102 aa) | TRAPP II ; Ypt1p,31p, 32p-GEF | *YALI0B22726g* (1129 aa) | 39% (462/1161) | 14% | 5 e-25 | 1 | 1 | 1 |
| Sec2p (759 aa) | Exocytosis, Sec4p-GEF | *YALI0F27379g* (661 aa) | 49% (85/172) | 24% | 2 e-12 | 1 | 1 | 1 |
| Rgp1p (663 aa) | Endosome-Golgi, Ypt6p-GEF | *YALI0F29513g* (713 aa) | 39% (217/546) | 16% | 9 e-9 | 1 | 1 | 1 |
| Ric1p (1056 aa) | Endosome-Golgi, Ypt6p-GEF | *YALI0A01397g* (939 aa, 1 intron) | 45% (64/141) | 5% | 3 e-4 | 1 | 1 | 1 |
| Vps9p (451 aa) | Vacuolar sorting, Ypt51-GEF | *YALI0E32593g* (548 aa) | 53% (262/489) | 13% | 3 e-67 | 1 | 1 | 1 |
| Vps39p (1049 aa) | Class C Vps, Ypt7-GEF | *YALI0B11550g* (1013 aa) | 43% (216/494) | 12% | 3 e-33 | 1 | 1 | 1 |
| **GAP, Gyp-like protein (Gyl)** |  |  |  |  |  |  |  |  |
| Gyp1p (637 aa) | Cis-Golgi Ypt1p-GAP,  Exocytosis Sec4p-GAP | *YALI0B12100g* (594 aa, 1 intron) | 66% (206/310) | 7% | 4 e-79 | 1 | 1 | 1 |
| Gyp5p (894 aa) | ER-Golgi Ypt1p-GAP,  Exocytosis Sec4p-GAP | *YALI0F18106g* (696 aa, 1 intron) | 53% (304/567) | 9% | 1 e-94 | 1 | 1 | 1 |
| Gyp8p (497 aa) | ER-Golgi, Ypt1p-GAP | *YALI0C24332g* (457 aa) | 43% (148/340) | 15% | 4 e-24 | 1 | 1 | 1 |
| Gyp3p (633 aa) | Exocytosis, Sec4p-GAP | *YALI0B22792g* (660 aa) | 62% (268/432) | 15% | e-103 | 1 | 1 | 1 |
| Gyp4p (492 aa) | Exocytosis, Sec4p-GAP | *YALI0B22792g* (660 aa) | 60% (251/415) | 15% | 5 e-94 | 0 | 0 | 0 |
| Gyp2p (950 aa) | Recycling, Ypt6p, Sec4p-GAP | *YALI0B01628g* (967 aa) | 61% (496/807) | 7% | 0.0 | 1 | 1 | 1 |
| Gyp6p (458 aa) | Vesicle transport, Ypt6-GAP | *YALI0C22968g* (577 aa, 1 intron) | 43% (108/248) | 19% | 4 e-10 | 1 | 1 | 1 |
| Gyp7p (746 aa) | Vesicle transport, Ypt7-GAP | *YALI0F31911g* (730 aa) | 55% (415/749) | 8% | e-121 | 1 | 1 | 1 |
| Gyl1p (720 aa) | Exocytosis, Gyp5p-interacting protein | no hits |  |  |  | 1 | 0 | 0 |
|  |  |  |  |  |  |  |  |  |
| **8.TRAPP complex proteins** |  |  |  |  |  |  |  |  |
| Bet5p (159 aa) | TRAPP I, II | *YALI0B10318g* (189 aa, 1 intron) | 46% (84/179) | 25% | 4 e-18 | 1 | 1 | 1 |
| Trs20p (175 aa) | TRAPP I, II | *YALI0E03520g* (141 aa) | 54% (96/177) | 22% | 1 e-26 | 1 | 1 | 1 |
| Bet3p (193 aa) | TRAPP I, II | *YALI0C08782g* (190 aa, 1 intron) | 77% (146/189) | 1% | 4 e-63 | 1 | 1 | 1 |
| Trs23p (219 aa) | TRAPP I, II | *YALI0B22396g* (135 aa) | 44% (96/218) | 38% | 3 e-17 | 1 | 1 | 1 |
| Trs31p (283 aa) | TRAPP I, II | *YALI0B05720g* (281 aa) | 47% (124/259) | 23% | 6 e-26 | 1 | 1 | 1 |
| Trs33p (268 aa) | TRAPP I, II | *YALI0E22902g* (178 aa) | 60% (75/123) | 6% | 4 e-17 | 1 | 1 | 1 |
| Trs85p (698 aa) | TRAPP I, II | *YALI0E12177g* (573 aa) | 41% (223/537) | 20% | 7 e-21 | 1 | 1 | 1 |
| Trs65p (560 aa) | TRAPP II | *YALI0B12144g* (608 aa) | 40% (77/91) | 14% | 0.002 | 1 | 1 | 1 |
| Trs120p (1289 aa) | TRAPP II | *YALI0D15004g* (1097 aa) | 39% (494/1243) | 18% | 3 e-45 | 1 | 1 | 1 |
| Trs130p (1102 aa) | TRAPP II | *YALI0B22726g* (1129 aa) | 39% (462/1161) | 14% | 5 e-25 | 1 | 1 | 1 |
|  |  |  |  |  |  |  |  |  |
| **9.COG (Conserved Oligomeric Golgi) complex proteins** |  |  |  |  |  |  |  |  |
| Cog1p (417 aa) |  | No hits |  |  |  | 1 | 1 | 0 |
| Cog2p (262 aa) | Sec35p | *YALI0A20592g* (217 aa) | No hits |  |  | 1 | 1 | 1 |
| Cog3p (801 aa) | Sec34p | *YALI0F17666g* (748 aa) | 46% (380/810) | 13% | 9 e-77 | 1 | 1 | 1 |
| Cog4p (861 aa) | Sec38p | *YALI0D03113g* (674 aa) | 41% (345/822) | 19% | 1 e-52 | 1 | 1 | 1 |
| Cog5p (403 aa) |  | *YALI0F05280g* (371 aa) | 43% (154/355) | 6% | 7 e-19 | 1 | 1 | 1 |
| Cog6p (839 aa) |  | *YALI0F16467g* (599 aa) | 41% (274/656) | 17% | 1 e-20 | 1 | 1 | 1 |
| Cog7p (279 aa) |  | No hits |  |  |  | 1 | 1 | 1 |
| Cog8p (607 aa) |  | *YALI0F16203g* (365 aa) | 47% (84/176) | 6% | 1 e-14 | 1 | 1 | 1 |
|  |  |  |  |  |  |  |  |  |
| **10.Uso1, Imh1, Rud3, Coy1 and Grh1 proteins** |  |  |  |  |  |  |  |  |
| Uso1p (1790 aa) | Coiled-coil protein | *YALI0F02387g* (1906 aa, 1 intron) | 45% (828/1806) | 12% | e-174 | 1 | 1 | 1 |
| Imh1p (1178 aa) | Coiled-coil protein | *YALI0F30855g* (1178 aa) | 38% (271/707) | 7% | 7 e-25 | 1 | 1 | 1 |
| Rud3p (489 aa) | Coiled-coil protein | *YALI0B18634g* (577 aa) | 48% (245/501) | 14% | 4 e-47 | 1 | 1 | 1 |
| Coy1p (599 aa) | Coiled-coil protein | *YALI0C18821g* (599 aa) | 52% (331/631) | 7% | 2 e-87 | 1 | 1 | 1 |
| Grh1p (372 aa) |  | *YALI0E03014g* (377 aa) | 44% (154/349) | 14% | 2 e-25 | 1 | 1 | 1 |
|  |  |  |  |  |  |  |  |  |
| **11. Dsl1p complex** |  |  |  |  |  |  |  |  |
| Dsl1p (754 aa) |  | *YALI0A07689g* (768 aa) | 39% (194/490) | 13% | 4 e-9 | 1 | 1 | 1 |
| Tip20p (701 aa) | ER protein | *YALI0C15334g* (709 aa) | 37% (254/669) | 16% | 3 e-4 | 1 | 1 | 1 |
| Dsl3p (709 aa) | Sec39p | *YALI0B12914g* (652 aa) | 39% (257/657) | 17% | 1 e-15 | 1 | 1 | 1 |
|  |  |  |  |  |  |  |  |  |
| **12.Arf, Arf-like proteins and Arl3p localization** |  |  |  |  |  |  |  |  |
| Arf1p (181 aa) |  | *YALI0F02167g* (181 aa) | 90% (163/181) | 0% | 1 e-82 | 1 | 0 | 1 |
| Arf2p (181 aa) |  | *YALI0F02167g* (181 aa) | 90% (162/179) | 0% | 1 e-82 | 1 | 1 | 1 |
| Arf3p (183 aa) | = Arl2p | *YALI0C14586g* (178 aa) | 85% (152/177) | 0% | 4 e-66 | 0 | 1 | 1 |
| Sar1p (190 aa) |  | *YALI0C21824g* (190 aa, 1 intron) | 82% (156/188) | 1% | 4 e-76 | 1 | 1 | 1 |
| Arl1p (183 aa) |  | *YALI0F31009g* (183 aa, 1 intron) | 87% (161/183) | 0% | 5 e-75 | 1 | 1 | 1 |
| Arl2p (183 aa) | = Arf3p | *YALI0C14586g* (178 aa) | 85% (152/177) | 0% | 4 e-66 | 0 | 1 | 1 |
| Arl3p (198 aa) |  | *YALI0D02995g* (190 aa, 2 introns) | 76% (144/188) | 0% | 2 e-63 | 1 | 1 | 1 |
| Cin4p (191 aa) | Arl2p-like | *Yl*Cin4p (189 aa, 1 intron)  (S. Casaregola, personal communication) | 63% (112/177) | 4% | 3 e-34 | 1 | 1 | 1 |
|  | Arl1p-like | *YALI0E11803g* (182 aa) |  |  |  | 0 | 1 | 0 |
| Sys1p (203 aa) |  | *YALI0B18656g* (163 aa) | 66% (98/147) | 4% | 5 e-32 | 1 | 1 | 1 |
| Mak3p (176 aa) | NatC complex | *YALI0C11539g* (194 aa) | 68% (123/179) | 4% | 6 e-47 | 1 | 1 | 1 |
| Mak10p (733 aa) | NatC complex | *YALI0F08591g* (712 aa) | 37% (254/677) | 13% | 3 e-22 | 1 | 1 | 1 |
| Mak31p (88 aa) | NatC complex | No hits |  |  |  | 1 | 1 | 1 |
|  |  |  |  |  |  |  |  |  |
| **13.GARP (Golgi-Associated Retrograde Protein) complex** |  |  |  |  |  |  |  |  |
| Vps51p (164 aa) |  | No hits |  |  |  | 1 | 1 | 0 |
| Vps52 (641 aa) |  | *YALI0F07381g* (757 aa) | 46% (283/615) | 18% | 1 e-49 | 1 | 1 | 1 |
| Vps53p (822 aa) |  | *YALI0D11198g* (735 aa) | 48% (341/704) | 10% | 2 e-63 | 1 | 1 | 1 |
| Vps54p (889 aa) |  | *YALI0B16822g* (1137 aa, 1 intron) | 43% (341/786) | 12% | 4 e-48 | 1 | 1 | 1 |
|  |  |  |  |  |  |  |  |  |
| **14.HOPS (HOmotypic fusion and vacuole Protein Sorting, Class C Vps complex) complex** |  |  |  |  |  |  |  |  |
| Vps11p (1029 aa) |  | *YALI0E23408g* (1019 aa) | 51% (533/1035) | 11% | e-122 | 1 | 1 | 1 |
| Vps18p (918 aa) |  | *YALI0A19008g* (948 aa) | 46% (451/973) | 12% | 9 e-67 | 1 | 1 | 1 |
| Vps16p (798 aa) |  | *YALI0A03553g* (840 aa, 1 intron) | 50% (266/527) | 4% | 2 e-54 | 1 | 1 | 1 |
| Vps41p (992 aa) | Rab-GEF | *YALI0F17710g* (1032 aa) | 51% (503/986) | 8% | e-129 | 1 | 1 | 1 |
| Vps39p (1049 aa) | Rab-GEF | *YALI0B11550g* (1013 aa) | 43% (216/494) | 12% | 3 e-33 | 1 | 1 | 1 |
| Vps33p (691 aa) | SNARE-binding protein ATP binding | *YALI0F04125g* (641 aa) | 44% (260/579) | 12% | 2 e-30 | 1 | 1 | 1 |
|  |  |  |  |  |  |  |  |  |
| **15.Exocyst complex proteins** |  |  |  |  |  |  |  |  |
| Sec3p (1336 aa) |  | *YALI0F21681g* (1502 aa, 1 intron) | 71% (114/160) | 1% | 5 e-41 | 1 | 1 | 1 |
| Sec8p (1065 aa) |  | *YALI0E33759g* (1089 aa) | 46% (512/1096) | 8% | 4 e-93 | 1 | 1 | 1 |
| Sec5p (971 aa) |  | *YALI0A19052g* (897 aa) | 39% (335/854) | 18% | 1 e-46 | 1 | 1 | 1 |
| Sec15p (910 aa) |  | *YALI0F12969g* (829 aa) | 44% (368/826) | 11% | 7 e-64 | 1 | 1 | 1 |
| Sec10p (871 aa) |  | *YALI0C01595g* (789 aa, 1 intron) | 48% (246/504) | 8% | 8 e-51 | 1 | 1 | 1 |
| Sec6p (805 aa) |  | *YALI0D08492g* (807 aa) | 51% (407/790) | 5% | e-103 | 1 | 1 | 1 |
| Exo84p (753 aa) |  | *YALI0F11143g* (657 aa) | 45% (265/588) | 13% | 4 e-44 | 1 | 1 | 0 |
| Exo70p (623 aa) |  | *YALI0C11946g* (603 aa) | 47% (299/630) | 7% | 3 e-62 | 1 | 1 | 1 |
|  |  |  |  |  |  | 1 | 1 | 1 |
| **16.Exocyst regulation proteins** |  |  |  |  |  |  |  |  |
| Rho1p (209 aa) |  | *YALI0E23001g* (203 aa) | 89% (179/201) | 2% | 7 e-87 | 1 | 1 | 1 |
| Rho3p (231 aa) |  | *YALI0F17270g* (204 aa, 1 intron) | 69% (160/229) | 12% | 2 e-76 | 1 | 1 | 0 |
| Cdc42p (191 aa) |  | *YALI0B15752g* (191 aa, 2 introns) | 94% (181/191) | 0% | 1 e-96 | 1 | 1 | 1 |
|  |  |  |  |  |  |  |  |  |
| **17.SNARE proteins** |  |  |  |  |  |  |  |  |
| **Qa** |  |  |  |  |  |  |  |  |
| Ufe1p (346 aa) | Endoplasmic reticulum | *YALI0C10846g* (339 aa) | 42% (154/360) | 10% | 8 e-20 | 1 | 1 | 1 |
| Sed5p (340 aa) | Golgi | *YALI0C16819g* (306 aa) | 57% (198/344) | 8% | 4 e-57 | 1 | 1 | 1 |
| Tlg2p (397 aa) | Golgi | *YALI0E33165g* (370 aa) | 52% (208/399) | 16% | 8 e-50 | 1 | 1 | 1 |
| Pep12p (288 aa) | Golgi-Vacuole | *YALI0E11825g* (259 aa)  *YALI0C22275g* (273 aa, 1 intron) | 52% (136/261)  43% (105/241) | 5%  8% | 1 e-24  2 e-17 | 1  0 | 1  0 | 1  1 |
| Vam3p (283 aa) | Vacuole | No hits |  |  |  | 1 | 1 | 0 |
| Sso1p (290 aa) | Plasma membrane | *YALI0D25872g* (292 aa)  *YALI0B10780g* (324 aa, 1 intron)  *YALI0E23243g* (278 aa) | 68% (189/276)  62% (173/278)  69% (171/245) | 1%  1%  0% | 3 e-63  8 e-57  3 e-60 | 1 | 0 | 0 |
| Sso2p (295 aa) | Plasma membrane | *YALI0D25872g* (292 aa)  *YALI0B10780g* (324 aa, 1 intron)  *YALI0E23243g* (278 aa) | 66% (182/273)  60% (173/284)  67% (168/248) | 1%  3%  0% | 2 e-62  3 e-59  3 e-59 | 1 | 1 | 1 |
| **Qb** |  |  |  |  |  |  |  |  |
| Sec20p (383 aa) | Endoplasmic reticulum | *YALI0C05302g* (321 aa) | 60% (72/119) | 0% | 5 e-14 | 1 | 1 | 1 |
| Bos1p (244 aa) | Endoplasmic reticulum-Golgi | *YALI0F31669g* (226 aa) | 47% (117/245) | 9% | 7 e-25 | 1 | 1 | 1 |
| Gos1p (223 aa) | Endoplasmic reticulum-Golgi | *YALI0D23353g* (219 aa) | 61% (127/205) | 0% | 8 e-36 | 1 | 1 | 1 |
| Vti1p (217 aa) | Golgi-Vacuole | *YALI0B02244g* (219 aa, 2 introns) | 59% (116/196) | 3% | 2 e-33 | 1 | 1 | 1 |
| Sec9p (651 aa) | Plasma membrane | *YALI0E18414g* (608 aa) | 46% (307/662) | 10% | 3 e-71 | 1 | 1 | 1 |
| Spo20p (397 aa) | Plasma membrane | No hits |  |  |  | 0 | 0 | 0 |
| **Qc** |  |  |  |  |  |  |  |  |
| Slt1p (245 aa) | Endoplasmic reticulum | *YALI0C21626g* (266 aa) | 46% (81/173) | 9% | 4 e-7 | 1 | 1 | 1 |
| Sft1p (97 aa) | Golgi | *YALI0E05269g* (93 aa, 1 intron) | 71% (56/78) | 2% | 2 e-13 | 1 | 1 | 1 |
| Bet1p (142 aa) | Golgi | *YALI0F25223g* (159 aa, 1 intron) | 57% (55/96) | 0% | 5 e-15 | 1 | 1 | 1 |
| Tlg1p (224 aa) | Golgi | *YALI0B05786g* (219 aa) | 50% (112/222) | 6% | 3 e-23 | 1 | 1 | 1 |
| Syn8p (255 aa) | Golgi-Vacuole | *YALI0E17875g* (254 aa) | 46% (94/204) | 20% | 4 e-7 | 1 | 1 | 1 |
| Vam7p (316 aa) | Vacuole | *YALI0C16412g* (345 aa) | 42% (127/300) | 12% | 2 e-11 | 1 | 1 | 1 |
| Sec9p (651 aa) | Plasma membrane | *YALI0E18414g* (608 aa) | 46% (307/662) | 10% | 3 e-71 | 1 | 1 | 1 |
| Spo20p (397 aa) | Plasma membrane | No hits |  |  |  | 0 | 0 | 0 |
| **R** |  |  |  |  |  |  |  |  |
| Sec22p (214 aa) | Endoplasmic reticulum-Golgi | *YALI0D21956g* (213 aa) | 70% (150/212) | 0% | 6 e-55 | 1 | 1 | 1 |
| Ykt6p (200 aa) | Golgi-Vacuole | *YALI0E21329g* (200 aa) | 80% (161/200) | 0% | 4 e-72 | 1 | 1 | 1 |
| Nyv1p (253 aa) | Golgi-Vacuole | *YALI0B04026g* (226 aa, 1 intron) | 47% (87/182) | 9% | 4 e-12 | 1 | 1 | 1 |
| Snc1p (117 aa) | Plasma membrane-Vesicles | *YALI0A03113g* (115 aa)  *YALI0E00594g* (112 aa, 1 intron) | 81% (94/116)  76% (71/93) | 2%  4% | 3 e-39  5 e-29 | 1 | 0 | 0 |
| Snc2p (115 aa) | Plasma membrane-Vesicles | *YALI0A03113g* (115 aa)  *YALI0E00594g* (112 aa, 1 intron) | 83% (96/115)  86% (80/92) | 1%  3% | 9 e-39  1 e-35 | 1 | 1 | 1 |
|  |  |  |  |  |  |  |  |  |
| **18.SNARE binding proteins** |  |  |  |  |  |  |  |  |
| Sly1p (666 aa) | Golgi Sed5p-binding  ER Ufe1p-binding | *YALI0D20416g* (590 aa, 1 intron) | 53% (347/649) | 10% | e-106 | 1 | 1 | 1 |
| Vps33p (691 aa) | Vacuole Vam3p-binding  Late endosome Pep12p-binding | *YALI0F04125g* (641 aa) | 44% (260/579) | 12% | 2 e-30 | 1 | 1 | 1 |
| Vps45p (577 aa) | Tlg2p/Pep12p-binding | *YALI0E29337g* (579 aa) | 52% (355/680) | 7% | 7 e-91 | 1 | 1 | 1 |
| Sec1p (724 aa) | Ssop-binding | *YALI0E22044g* (713 aa, 1 intron) | 52% (355/680) | 10% | 2 e-93 | 1 | 1 | 1 |
|  |  |  |  |  |  |  |  |  |
| **19.Exocytosis SNARE regulation proteins** |  |  |  |  |  |  |  |  |
| Vsm1p (428 aa) |  | *YALI0B06754g* (397 aa) | 49% (210/426) | 16% | 2 e-50 | 1 | 1 | 1 |
| Tpd3p (635 aa) | Ceramide activated protein phosphatase (CAPP) regulatory subunit | *YALI0F00836g* (622 aa) | 66% (426/638) | 3% | e-155 | 2 | 1 | 1 |
| Cdc55p (526 aa) | CAPP regulatory subunit | *YALI0F03223g* (474 aa, 1 intron) | 63% (184/292) | 2% | 1 e-75 | 1 | 1 | 1 |
| Sit4p (311 aa) | CAPP catalytic subunit | *YALI0F14069g* (319 aa) | 73% (223/305) | 1% | e-106 | 1 | 1 | 1 |
| Tpk1p (397 aa)  Tpk2p (380 aa)  Tpk3p (398 aa) | cAMP-dependent protein kinase (PKA) | *YALI0C08305g* (330 aa) | 86% (279/324)  89% (296/329)  85% (274/320) | 0%  0%  0% | e-147  e-165  e-147 | 2 | 2 | 2 |
|  |  |  |  |  |  |  |  |  |
| **20.SNARE recycling proteins** |  |  |  |  |  |  |  |  |
| Sec17p (292 aa) | NSF attachment protein, mammalian α- SNAP-homologue | *YALI0C23947g* (292 aa) | 61% (177/289) | 0% | 2 e-60 | 1 | 1 | 1 |
| Sec18p (758 aa) | ATPase, mammalian NSF-homologue | *YALI0E29249g* (809 aa) | 68% (498/726) | 1% | 0.0 | 2 | 1 | 1 |
| Rcy1p (840 aa) |  | *YALI0B19074g*/Sls2p (912 aa) | 41% (305/734) | 16% | 5 e-33 | 1 | 1 | 1 |
