# Supplementary material for: Yarrowia lipolytica vesicle-mediated protein transport pathways
Source: BMC Evol Biol. 2007 Nov 12;7:219. doi: 10.1186/1471-2148-7-219 (PMC2241642; doi:10.1186/1471-2148-7-219)
Supplement: Additional file 9 — List of Candida. glabrata, Kluyveromyces lactis, Debaryomyces hansenii genes coding for the proteins potentially implicated in vesicular transport. [file 1471-2148-7-219-S9.doc]

Additional file 9: List of *Candida glabrata*, *Kluyveromyces lactis* and *Debaryomyces hansenii* genes potentially implicated in vesicular secretion.

| *Sc* | *Cg* | *Kl* | *Dh* |
| --- | --- | --- | --- |
|  |  |  |  |
| **1.COPII vesicle coat proteins** |  |  |  |
| Sar1p (190 aa) | *CAGL0E05896g* | *KLLA0B02046g* | *DEHA0C04686g* |
| Sec23p (768 aa) | *CAGL0H00242g*  *CAGL0G09911g* | *KLLA0E04807g* | *DEHA0E03025g*  *DEHA0A05456g* |
| Yhr035wp (630 aa) |  | *KLLA0C10461g* |  |
| Sec24p (926 aa) | *CAGL0D01078g*  *CAGL0C01353g* | *KLLA0F03729g* | *DEHA0D03542g* |
| Sfb2p (876 aa) |  |  |  |
| Sfb3p (929 aa) | *CAGL0A01848g* | *KLLA0F22110g* | *DEHA0E21956g* |
| Sec13p (297 aa) | *CAGL0J08778g*  *CAGL0I03454g* | *KLLA0C16643g* | *DEHA0G09053g* |
| Sec31p (1273 aa) | *CAGL0J08998g* | *KLLA0F05159g* | *DEHA0D15752g* |
| Sec16p (2195 aa) | *CAGL0H05577g* | *KLLA0E23958g* | *DEHA0B02871g* |
| Sec12p (471 aa) | *CAGL0M10703g* | *KLLA0C08723g* | *DEHA0F13409g* |
| Sed4p (1065 aa) | *CAGL0J03564g* |  |  |
|  |  |  |  |
| **2.COPI vesicle coat proteins** |  |  |  |
| Arf1p (181aa) | *CAGL0I03916g* |  | *DEHA0B14399g* |
| Sec33p (1201 aa) | *CAGL0I03718g* | *KLLA0F04884g* | *DEHA0D16929g* |
| Ret2p (546 aa) | *CAGL0A04741g* | *KLLA0D11396g* | *DEHA0F15543g* |
| Ret3p (189 aa) | *CAGL0L08800g* | *KLLA0F27313g* | *DEHA0F21450g* |
| Sec21p (935 aa) | *CAGL0M03531g* | *KLLA0E13299g* | *DEHA0A03795g* |
| Sec26p (973 aa) | *CAGL0K11088g* | *KLLA0C15279g* | *DEHA0F20548g* |
| Sec27p (889 aa) | *CAGL0H08932g* | *KLLA0B01958g* | *DEHA0C04598g* |
| Sec28p (296 aa) | *CAGL0M12903g* | *KLLA0C09174g* | *DEHA0B10428g* |
|  |  |  |  |
| **3.AP complex** |  |  |  |
| **AP-1** |  |  |  |
| Apl2p (726 aa) | *CAGL0D02068g* | *KLLA0E12265g* | *DEHA0F19041g* |
| Apl4p (832 aa) | *CAGL0K05225g* | *KLLA0E06556g* | *DEHA0B11484g* |
| Apm1p (475 aa) | *CAGL0K00539g* | *KLLA0D14311g* | *DEHA0D15356g* |
| Aps1p (156 aa) | *CAGL0B04983g* | *KLLA0F22814g* | *DEHA0F18183g* |
| Apm2p (605 aa) | *CAGL0K03223g* | *KLLA0F25432g* | *DEHA0G09361g* |
| **AP-2** |  |  |  |
| Apl1p (700 aa) | *CAGL0G04103g* | *KLLA0E09504g* | *DEHA0F26334g* |
| Apl3p (1025 aa) | *CAGL0F04389g* | *KLLA0B12243g* | *DEHA0F27126g* |
| Apm4p (491 aa) | *CAGL0C05203g* | *KLLA0C03894g* | *DEHA0C11000g* |
| Aps2p (147 aa) | *CAGL0H01859g* | *KLLA0B06545g* | *DEHA0F11187g* |
| **AP-3** |  |  |  |
| Apl6p (809 aa) | *CAGL0F08393g* | *KLLA0A09559g* | *DEHA0G22814g* |
| Apl5p (932 aa) | *CAGL0G09174g* | *KLLA0E19503g* | *DEHA0G01870g* |
| Apm3p (483 aa) | *CAGL0L02145g* | *KLLA0E18876g* | *DEHA0G05698g* |
| Aps3p (194 aa) | *CAGL0D03388g* | *KLLA0F15268g* | *DEHA0G14971g* |
|  |  |  |  |
| **Clathrin** |  |  |  |
| Chc1p | *CAGL0A03718g* | *KLLA0F09911g* | *DEHA0E05676g* |
| Clc1p | *CAGL0I08833g* | *KLLA0F14619g* | *DEHA0B03234g* |
|  |  |  |  |
| **Other adaptors** |  |  |  |
| Gga1p (557 aa) |  |  |  |
| Gga2p (585 aa) | *CAGL0A02629g* | *KLLA0E21560g* | *DEHA0G16786g* |
| Inp53p (1107 aa) | *CAGL0B04631g* | KLLA0F23551g | DEHA0A05610g |
|  |  |  |  |
| **4.Retromer complex proteins** |  |  |  |
| Vps5p (312 aa) | *CAGL0L10472g* | *KLLA0F16643g* | *DEHA0E09361g* |
| Vps17p (551 aa) | *CAGL0I07271g* | *KLLA0E02904g* | *DEHA0G05434g* |
| Vps26p (379 aa) | *CAGL0J04994g* | *KLLA0F01419g* | *DEHA0A12463g* |
| Vps29p (282 aa) | *CAGL0E04422g* | *KLLA0E03311g* | *DEHA0G07953g* |
| Vps35p (944 aa) | *CAGL0I06072g* | *KLLA0B07535g* | DhVps35p (S. Casaregola, personal communication) |
|  |  |  |  |
| **5.Sorting nexin proteins** |  |  |  |
| Snx4p (423 aa) | *CAGL0J01001g* | *KLLA0C10967g* | *DEHA0A02299g* |
| Snx41p (625 aa) | *CAGL0F03113g* | *KLLA0B00803g* | *DEHA0A02475g* |
| Snx42p (640 aa) | *CAGL0H10428g* | *KLLA0E09141g* | *DEHA0G18205g* |
| Snx3p (162 aa) | *CAGL0G06424g* | *KLLA0A03718g* | *DEHA0F04312g* |
|  |  |  |  |
| **6.Ypt proteins** |  |  |  |
| Ypt1p (206 aa) | *CAGL0K12672g* | *KLLA0D05313g* | *DEHA0B07095g* |
| Ypt6p (215 aa) | *CAGL0K06017g* | *KLLA0F20471g* | *DEHA0E06699g* |
| Ypt7p (208 aa) | *CAGL0E02607g* | *KLLA0D01265g* | *DEHA0D03113g*  *DEHA0D08844g* |
| Ypt10p (199 aa) | *CAGL0I09306g* |  |  |
| Ypt11p (355 aa) | *CAGL0M03817g* | *KLLA0C12881g* |  |
| Ypt31p (223 aa) | *CAGL0C02453g* | *KLLA0B00671g* |  |
| Ypt32p (222 aa) | *CAGL0K09394g* |  | *DEHA0B03608g* |
| Ypt51p (210 aa) | *CAGL0J08635g* | *KLLA0C13728g* | *DEHA0A11451g* |
| Ypt52p (234 aa) | *CAGL0G07689g* | *KLLA0D02376g* | *DEHA0B01122g*  *DEHA0A02585g* |
| Ypt53p (220 aa) |  | *KLLA0F01232g* |  |
| Sec4p (215 aa) | *CAGL0F02123g* | *KLLA0E12111g* | *DEHA0F08382g* |
|  |  |  |  |
| **7.Ypt regulation** |  |  |  |
| **Prenylation** |  |  |  |
| Bet2p (325 aa) | *CAGL0G09823g* | *KLLA0E04719g* | *DEHA0E03575g* |
| Bet4p (327 aa) | *CAGL0J01067g* | *KLLA0D09460g* | *DEHA0D17787g* |
| Mrs6p (603 aa) | *CAGL0J07260g* | *KLLA0D06237g* | *DEHA0F03025g* |
| **GDI** |  |  |  |
| Gdi1p (451 aa) | *CAGL0K01925g* | *KLLA0F09185g* | *DEHA0F26620g* |
| **GDF** |  |  |  |
| Yos1p (85 aa) |  |  | *DEHA0G04653g* |
| Yif1p (314 aa) | *CAGL0J08052g* | *KLLA0C02563g* | *DEHA0E23232g* |
| Yip1p (248 aa) | *CAGL0I08701g* | *KLLA0F15015g* | *DEHA0G21417g* |
| Yip2p (180 aa) | *CAGL0K05203g* | *KLLA0E06578g* | *DEHA0B11462g* |
| Yip3p (176 aa) | *CAGL0G01452g* | *KLLA0F03905g* | *DEHA0G16478g* |
| Yip4p (235 aa) | *CAGL0D01760g* | *KLLA0F12474g* | *DEHA0F03388g* |
| Yip5p (310 aa) | *CAGL0I04268g* | *KLLA0A03113g* | *DEHA0E14982g* |
| **GEF** |  |  |  |
| Bet5p (159 aa) | *CAGL0H04345g* | *KLLA0B04609g* | *DEHA0D12562g* |
| Trs20p (175 aa) | *CAGL0D05346g* | *KLLA0D15763g* | *DEHA0D16148g* |
| Bet3p (193 aa) | *CAGL0K08140g* | *KLLA0F16126g* | *DEHA0E07513g* |
| Trs23p (219 aa) | *CAGL0K11253g* | *KLLA0D12144g* | *DEHA0C02706g* |
| Trs31p (283 aa) | *CAGL0K06589g* | *KLLA0E03267g* | *DEHA0G05214g* |
| Trs33p (268 aa) | *CAGL0I02772g* | *KLLA0F23826g* | *DEHA0F20812g* |
| Trs85p (698 aa) | *CAGL0B01705g* | *KLLA0F18953g* | *DEHA0E17578g* |
| Trs65p (560 aa) | *CAGL0I08855g* | *KLLA0F14641g* | *DEHA0E08173g* |
| Trs120p (1289 aa) | *CAGL0F02739g* | *KLLA0A02255g* | *DEHA0G06545g* |
| Trs130p (1102 aa) | *CAGL0F03949g* | *KLLA0C07128g* | *DEHA0E03773g* |
| Sec2p (759 aa) | *CAGL0J08228g* | *KLLA0C02299g* | *DEHA0E14916g* |
| Rgp1p (663 aa) | *CAGL0B01639g* | *KLLA0F11572g* | *DEHA0E22088g* |
| Ric1p (1056 aa) | *CAGL0A03564g* | *KLLA0F17259g* | *DEHA0D09680g* |
| Vps9p (451 aa) | *CAGL0H06941g* | *KLLA0D10263g* | *DEHA0A06479g* |
| Vps39p (1049 aa) | *CAGL0D00572g* | *KLLA0B02849g* | *DEHA0D12320g* |
| **GAP, Gyp-like protein (Gyl)** |  |  |  |
| Gyp1p (637 aa) | *CAGL0L10494g* | *KLLA0A07975g* | *DEHA0E04103g* |
| Gyp5p (894 aa) | *CAGL0H00737g* | *KLLA0D12584* | *DEHA0F03696g* |
| Gyp8p (497 aa) | *CAGL0K12452g* | *KLLA0F08789g* | *DEHA0F14157g* |
| Gyp3p (633 aa) | *CAGL0M03641g* | *KLLA0A07491g* | *DEHA0E12012g* |
| Gyp4p (492 aa) |  |  |  |
| Gyp2p (950 aa) | *CAGL0G07777g* | *KLLA0D15202g* | DhGyp2p (S. Casaregola, personal communication) |
| Gyp6p (458 aa) | *CAGL0J00825g* | *KLLA0E11869g* | *DEHA0E20449g* |
| Gyp7p (746 aa) | *CAGL0C05489g* | *KLLA0D19272g* | *DEHA0C14003g* |
| Gyl1p (720 aa) | *CAGL0K10934g* |  |  |
|  |  |  |  |
| **8.TRAPP complex proteins** |  |  |  |
| Bet5p (159 aa) | *CAGL0H04345g* | *KLLA0B04609g* | *DEHA0D12562g* |
| Trs20p (175 aa) | *CAGL0D05346g* | *KLLA0D15763g* | *DEHA0D16148g* |
| Bet3p (193 aa) | *CAGL0K08140g* | *KLLA0F16126g* | *DEHA0E07513g* |
| Trs23p (219 aa) | *CAGL0K11253g* | *KLLA0D12144g* | *DEHA0C02706g* |
| Trs31p (283 aa) | *CAGL0K06589g* | *KLLA0E03267g* | *DEHA0G05214g* |
| Trs33p (268 aa) | *CAGL0I02772g* | *KLLA0F23826g* | *DEHA0F20812g* |
| Trs85p (698 aa) | *CAGL0B01705g* | *KLLA0F18953g* | *DEHA0E17578g* |
| Trs65p (560 aa) | *CAGL0I08855g* | *KLLA0F14641g* | *DEHA0E08173g* |
| Trs120p (1289 aa) | *CAGL0F02739g* | *KLLA0A02255g* | *DEHA0G06545g* |
| Trs130p (1102 aa) | *CAGL0F03949g* | *KLLA0C07128g* | *DEHA0E03773g* |
|  |  |  |  |
| **9.COG (Conserved Oligomeric Golgi) complex proteins** |  |  |  |
| Cog1p (417 aa) | *CAGL0I06919g* | *KLLA0A01430g* |  |
| Cog2p (262 aa) | *CAGL0L04884g* | *KLLA0B11121g* | *DEHA0E09955g* |
| Cog3p (801 aa) | *CAGL0I08569g* | *KLLA0C11715g* | *DEHA0C03025g* |
| Cog4p (861 aa) | *CAGL0G01760g* | *KLLA0F08184g* | *DEHA0C17380g* |
| Cog5p (403 aa) | *CAGL0G01298g* | *KLLA0F03685g* | *DEHA0D03564g* |
| Cog6p (839 aa) | *CAGL0G01496g* | *KLLA0F03993g* | *DEHA0E11330g* |
| Cog7p (279 aa) | *CAGL0A00539g* | *KLLA0A08888g* | *DEHA0A09317g* |
| Cog8p (607 aa) | *CAGL0L11418g* | *KLLA0B04730g* | *DEHA0F06160g* |
|  |  |  |  |
| **10.Uso1, Imh1, Rud3, Coy1 and Grh1 proteins** |  |  |  |
| Uso1p (1790 aa) | *CAGL0D00924g* | *KLLA0E05225g* | *DEHA0C09658g* |
| Imh1p (1178 aa) | *CAGL0E03454g* | *KLLA0C17204g* | *DEHA0A12507g* |
| Rud3p (489 aa) | *CAGL0L02695g* | *KLLA0D06875g* | *DEHA0C17248g* |
| Coy1p (599 aa) | *CAGL0M08162g* | *KLLA0A06402g* | *DEHA0B04829g* |
| Grh1p (372 aa) | *CAGL0F00583g* | *KLLA0C01133g* | *DEHA0B06710g* |
|  |  |  |  |
| **11. Dsl1p complex** |  |  |  |
| Dsl1p (754 aa) | *CAGL0J07964g* | *KLLA0C02695g* | *DEHA0E23441g* |
| Tip20p (701 aa) | *CAGL0E04972g* | *KLLA0E14894g* | *DEHA0G22462g* |
| Dsl3p (709 aa) | *CAGL0M00836g* | *KLLA0B05115g* | *DEHA0E05192g* |
|  |  |  |  |
| **12.Arf, Arf-like proteins and Arl3p localization** |  |  |  |
| Arf1p (181 aa) | *CAGL0I03916g* |  | *DEHA0B14399g* |
| Arf2p (181 aa) | *CAGL0J09064g* | *KLLA0F05225g* | *DEHA0E02651g* |
| Arf3p (183 aa) |  | *KLLA0C13563g* | *DEHA0A09559g* |
| Sar1p (190 aa) | *CAGL0E05896g* | *KLLA0B02046g* | *DEHA0C04686g* |
| Arl1p (183 aa) | *CAGL0I10835g* | *KLLA0E24805g* | *DEHA0G19613g* |
| Arl2p (183 aa) | *CAGL0L12826g* | *KLLA0C13563g* | *DEHA0A09559g* |
| Arl3p (198 aa) |  | *KLLA0E12837g* | *DEHA0B15521g* |
| Cin4p (191 aa) | *CAGL0I00858g* | *KLLA0F17072g* | *DEHA0C02200g* |
| Arlp-like |  | *KLLA0F02662g* |  |
| Sys1p (203 aa) | *CAGL0D03146g* | *KLLA0F15730g* | *DEHA0C18843g* |
| Mak3p (176 aa) | *CAGL0H08547g* | *KLLA0B12100g* | *DEHA0D19349g* |
| Mak10p (733 aa) | *CAGL0E04136g* | *KLLA0A01925g* | *DEHA0D19217g* |
| Mak31p (88 aa) | *CAGL0L07942g* | *KLLA0A11220g* | *DEHA0F25773g* |
|  |  |  |  |
| **13.GARP (Golgi-Associated Retrograde Protein) complex** |  |  |  |
| Vps51p (164 aa) | *CAGL0H06809g* | *KLLA0D02200g* |  |
| Vps52 (641 aa) | *CAGL0F05181g* | *KLLA0C03696g* | *DEHA0A13970g* |
| Vps53p (822 aa) | *CAGL0J01111g* | *KLLA0D09394g* | *DEHA0A13673g* |
| Vps54p (889 aa) | *CAGL0K11792g* | *KLLA0F04367g* | *DEHA0A01639g* |
|  |  |  |  |
| **14.HOPS (HOmotypic fusion and vacuole Protein Sorting, Class C Vps complex) complex** |  |  |  |
| Vps11p (1029 aa) | *CAGL0M04895g* | *KLLA0B13090g* | *DEHA0B05071g* |
| Vps18p (918 aa) | *CAGL0M04653g* | *KLLA0D17182g* | *DEHA0E20823g* |
| Vps16p (798 aa) | *CAGL0L12694g* | *KLLA0D11748g* | *DEHA0D14344g* |
| Vps41p (992 aa) | *CAGL0K10076g* | *KLLA0C11759g* | *DEHA0C03069g* |
| Vps39p (1049 aa) | *CAGL0D00572g* | *KLLA0B02849g* | *DEHA0D12320g* |
| Vps33p (691 aa) | *CAGL0C02607g* | *KLLA0B03267g* | *DEHA0E11198g* |
|  |  |  |  |
| **15.Exocyst complex proteins** |  |  |  |
| Sec3p (1336 aa) | *CAGL0H09680g* | *KLLA0D13530g* | *DEHA0D18117g* |
| Sec8p (1065 aa) | *CAGL0D01672g* | *KLLA0E11385g* | *DEHA0F16412g* |
| Sec5p (971 aa) | *CAGL0E00957g* | *KLLA0D17138g* | *DEHA0E13233g* |
| Sec15p (910 aa) | *CAGL0H07909g* | *KLLA0A01023g* | *DEHA0G17754g* |
| Sec10p (871 aa) | *CAGL0K08404g* | *KLLA0D18260g* | *DEHA0G17138g* |
| Sec6p (805 aa) | *CAGL0J03168g* | *KLLA0C07843g* | *DEHA0G04279g* |
| Exo84p (753 aa) | *CAGL0K12166g* | *KLLA0E11319g* |  |
| Exo70p (623 aa) | *CAGL0M03113g* | *KLLA0F14421g* | *DEHA0D04191g* |
|  |  |  |  |
| **16.Exocyst regulation proteins** |  |  |  |
| Rho1p (209 aa) | *CAGL0I08459g* | *KLLA0B10626g* | *DEHA0B11792g* |
| Rho3p (231 aa) | *CAGL0G08558g* | *KLLA0F03443g* |  |
| Cdc42p (191 aa) | *CAGL0F05269g* | *KLLA0A04213g* | *DEHA0G15037g* |
|  |  |  |  |
| **17.SNARE proteins** |  |  |  |
| **Qa** |  |  |  |
| Ufe1p (346 aa) | *CAGL0K05489g* | *KLLA0A07821g* | *DEHA0C03938g* |
| Sed5p (340 aa) | *CAGL0D01914g* | *KLLA0F17798g* | *DEHA0D09746g* |
| Tlg2p (397 aa) | *CAGL0G08932g* | *KLLA0A10681g* | *DEHA0G09724g* |
| Pep12p (288 aa) | *CAGL0I01012g* | *KLLA0E21857g* | *DEHA0G08767g*  *DEHA0F12540g* |
| Vam3p (283 aa) | *CAGL0G04807g* | *KLLA0C13233g* |  |
| Sso1p (290 aa) | *CAGL0E06160g* |  |  |
| Sso2p (295 aa) | *CAGL0L00561g* | *KLLA0C15961g* | *DEHA0B09790g* |
| **Qb** |  |  |  |
| Sec20p (383 aa) | *CAGL0I02948g* | *KLLA0C12300g* | *DEHA0A06611g* |
| Bos1p (244 aa) | *CAGL0L13200* | *KLLA0D05775g* | *DEHA0C01573g* |
| Gos1p (223 aa) | *CAGL0E03872g* | *KLLA0E00572g* | *DEHA0G25344g* |
| Vti1p (217 aa) | *CAGL0L10604g* | *KLLA0B10285g* | *DEHA0D12760g* |
| Sec9p (651 aa) | *CAGL0A01672g* | *KLLA0D02024g* | *DEHA0F20416g* |
| Spo20p (397 aa) |  |  |  |
| **Qc** |  |  |  |
| Slt1p (245 aa) | *CAGL0J05368g* | *KLLA0F06644g* | *DEHA0C05522g* |
| Sft1p (97 aa) | *CAGL0M10235g* | *KLLA0B13431g* | *DEHA0E21131g* |
| Bet1p (142 aa) | *CAGL0J02090g* | *KLLA0D13992g* | *DEHA0B09658g* |
| Tlg1p (224 aa) | *CAGL0K06501g* | *KLLA0E03443g* | *DEHA0G05368g* |
| Syn8p (255 aa) | *CAGL0H06325g* | *KLLA0F07777g* | *DEHA0A12881g* |
| Vam7p (316 aa) | *CAGL0I07095g* | *KLLA0A01672g* | *DEHA0A08250g* |
| Sec9p (651 aa) | *CAGL0A01672g* | *KLLA0D02024g* | *DEHA0F20416g* |
| Spo20p (397 aa) |  |  |  |
| **R** |  |  |  |
| Sec22p (214 aa) | *CAGL0C03179g* | *KLLA0F20251g* | *DEHA0F15961g* |
| Ykt6p (200 aa) | *CAGL0D03498g* | *KLLA0D02684g* | *DEHA0D08822g* |
| Nyv1p (253 aa) | *CAGL0C00429g* | *KLLA0F19448g* | *DEHA0G09474g* |
| Snc1p (117 aa) | *CAGL0G06358g* |  |  |
| Snc2p (115 aa) | *CAGL0E05258g* | *KLLA0A03883g* | *DEHA0D06160g* |
|  |  |  |  |
| **18.SNARE binding proteins** |  |  |  |
| Sly1p (666 aa) | *CAGL0F05665g* | *KLLA0D18480g* | *DEHA0G14047g* |
| Vps33p (691 aa) | *CAGL0C02607g* | *KLLA0B03267g* | *DEHA0E11198g* |
| Vps45p (577 aa) | *CAGL0H03003g* | *KLLA0E18920g* | *DEHA0A05192g* |
| Sec1p (724 aa) | *CAGL0E01001g* | *KLLA0D17028g* | *DEHA0E18370g* |
|  |  |  |  |
| **19.Exocytosis SNARE regulation proteins** |  |  |  |
| Vsm1p (428 aa) | *CAGL0I06787g* | *KLLA0E10318g* | *DEHA0F26576g* |
| Tpd3p (635 aa) | *CAGL0H06281g*  *CAGL0F00957g* | *KLLA0F07689g* | *DEHA0F05346g* |
| Cdc55p (526 aa) | *CAGL0L06182g* | *KLLA0F09053g* | *DEHA0B04587g* |
| Sit4p (311 aa) | *CAGL0K01331g* | *KLLA0D03652g* | *DEHA0D14410g* |
| Tpk1p (397 aa) |  |  |  |
| Tpk2p (380 aa) | *CAGL0G09020g* | *KLLA0D03190g* | *DEHA0F25212g*  *DEHA0B12628g* |
| Tpk3p (398 aa) | *CAGL0M08404g* | *KLLA0B07205g* |  |
|  |  |  |  |
| **20.SNARE recycling proteins** |  |  |  |
| Sec17p (292 aa) | *CAGL0F04653g* | *KLLA0E05874g* | *DEHA0C06061g* |
| Sec18p (758 aa) | *CAGL0E04642g*  *CAGL0M01782g* | *KLLA0C14520g* | DhSec18p (S. Casaregola, personal communication) |
| Rcy1p (840 aa) | *CAGL0F02497g* | *KLLA0C03300g* | *DEHA0E20075g* |
